# Molecular population genetics of the Polycomb genes in *Drosophila subobscura*

**DOI:** 10.1371/journal.pone.0185005

**Published:** 2017-09-14

**Authors:** Juan M. Calvo-Martín, Montserrat Papaceit, Carmen Segarra

**Affiliations:** Departament de Genètica, Microbiologia i Estadística, Facultat de Biologia, and Institut de Recerca de la Biodiversitat (IRBio), Universitat de Barcelona, Barcelona, Spain; University of Iceland, ICELAND

## Abstract

Polycomb group (PcG) proteins are important regulatory factors that modulate the chromatin state. They form protein complexes that repress gene expression by the introduction of posttranslational histone modifications. The study of PcG proteins divergence in *Drosophila* revealed signals of coevolution among them and an acceleration of the nonsynonymous evolutionary rate in the lineage ancestral to the *obscura* group species, mainly in subunits of the Pcl-PRC2 complex. Herein, we have studied the nucleotide polymorphism of PcG genes in a natural population of *D*. *subobscura* to detect whether natural selection has also modulated the evolution of these important regulatory genes in a more recent time scale. Results show that most genes are under the action of purifying selection and present a level and pattern of polymorphism consistent with predictions of the neutral model, the exceptions being *Su(z)12* and *Pho*. MK tests indicate an accumulation of adaptive changes in the SU(Z)12 protein during the divergence of *D*. *subobscura* and *D*. *guanche*. In contrast, the HKA test shows a deficit of polymorphism at *Pho*. The most likely explanation for this reduced variation is the location of this gene in the dot-like chromosome and would indicate that this chromosome also has null or very low recombination in *D*. *subobscura*, as reported in *D*. *melanogaster*.

## Introduction

The identification of genes under adaptive selection is a major goal of evolutionary genetics. Several methods and statistical tests have been developed to detect the footprint left by the action of positive selection at the molecular level (reviewed in [1]). These approaches were first applied to single candidate genes with a clear adaptive function. In *Drosophila*, these studies consistently corroborated that adaptive selection has shaped the evolution of genes involved in processes such as immunity [2], reproduction [3] and stem cell maintenance [4]. However, molecular evolution of proteins and thus of their encoding genes is not solely determined by selection on protein function [5]. In fact, proteins do not act in isolation, but they are members of complex metabolic, regulatory or interaction networks that control biological processes. The evolution of a protein can be affected by its location in the network, the connectivity with network partners and the evolution of the network as a whole [6]. The strength of purifying selection on genes coding for enzymes depends on the protein position either in the metabolic pathway [7] or in the signal transduction pathway [8,9]. In addition, physically interacting proteins tend to evolve co-ordinately. Accordingly, pervasive adaptive evolution has been detected among genes coding for subunits of nuclear pore complexes [10] and centromeric interacting proteins [11]. This concordant evolution also results in coincident increases or decreases of the evolutionary rate over particular branches of a phylogeny, as has been detected among genes encoding either interacting proteins [12] or members of protein networks [13].

Polycomb group (PcG) genes are involved in the epigenetic silencing of chromatin by the introduction of posttranslational histone modifications [14]. They code for proteins that form diverse protein repressive complexes. These interacting proteins are expected to evolve co-ordinately to maintain the integrity and functionality of the complexes, which can cause a correlation in their evolutionary rates. Evidence of coevolution among the subunits of these complexes has been detected in a previous study of interspecific nucleotide divergence in the *Drosophila* genus [15]. Moreover, this study revealed an acceleration in the nonsynonymous substitution rate in the lineage ancestral to the *obscura* group species, mainly in genes encoding subunits of the Pcl-PRC2 complex. Therefore, signatures of adaptive selection have been detected in some PcG genes prior diversification of the *obscura* species, i.e. in a rather distant time scale. Accordingly, it can be expected that positive selection have also modulated PcG genes evolution in a more recent time scale. The action of selection in the recent past can be detected by the study of intraspecific nucleotide polymorphism. The fixation of an adaptive mutation results in a strong reduction of linked neutral variation around the selected site (selective sweep). In contrast, the maintenance of different variants by balancing selection causes an increase of linked neutral variation [1].

The main aim of this study was to ascertain whether the action of positive selection detected in some Polycomb group genes in the lineage leading to the *obscura* group species is also detected by the analysis of nucleotide polymorphism in a species of this group. With this aim, we have analyzed by high-quality Sanger sequencing the nucleotide polymorphism of 16 PcG genes that code for the subunits of four Polycomb repressive complexes (PhoRC, Pcl-PRC2, PRC1 and dRAF) in *D*. *subobscura* as representative of the *obscura* group.

Nucleotide polymorphism in *D*. *subobscura* has been previously studied in multiple gene regions. Several of these studies have focused on analyzing how the level and pattern of nucleotide variation is affected by the presence of chromosomal inversions. Indeed, natural populations of *D*. *subobscura* have a rich chromosomal inversion polymorphism. More than 60 inversions that form about 90 complex chromosomal arrangements of overlapping inversions have been described in the five major chromosomes of the species: A, J, U, E and O [16]. The average number of inversions in heterozygosis per individual ranges from 2.4 to 5.1 in European populations, being 3.8 the mean value [17]. This rich chromosomal polymorphism causes nucleotide variation in *D*. *subobscura* to be strongly structured in regions affected by the inversions. This effect is due to the reduced recombination along the inverted region in heterokaryotypes, which prevents gene flow and results in a strong genetic differentiation between inverted and non-inverted (standard) chromosomes, mainly in gene regions located near the inversion breakpoints [18] but also along the whole inversion [19]. Accordingly, nucleotide variation at a particular gene in order to detect adaptive selection has to be studied in a random sample of arrangements that are homosequential for the region where the gene is located. This sampling strategy prevents any effect of inversion polymorphism on nucleotide variation.

In addition, one of the PcG genes studied (*Pho*) is located in the dot-like chromosome in *D*. *melanogaster* and other species of the *melanogaster* group [20]. This chromosome has in these species a wide footprint of reduced variation relative to the other autosomes [21–23]. This low variation is consistent with a virtual lack of recombination in this chromosome that was previously identified by genetic analysis in *D*. *melanogaster* [24]. No data on nucleotide variation at genes located in the dot-like chromosome of *D*. *subobscura* are available. As the gene content of chromosomal elements is highly conserved in the Drosophila genus [25], *Pho* is also expected to be located in the dot-like chromosome of *D*. *subobscura*. In this case, the level and pattern of nucleotide variation at *Pho* would enable to contrast whether the dot-like chromosome of this species also has a reduced recombination rate.

The results obtained indicate that nucleotide variation of the PcG genes has been mainly modelled by purifying selection in *D*. *subobscura*. In fact, evidence of positive selection was only detected at *Su(z)12*, which encodes a subunit of the Pcl-PRC2 complex. In addition, an extremely low level of variation was detected at *Pho* relative to the other PcG genes, which would support that the dot-like chromosome of *D*. *subobscura* also has null or very low recombination rate.

## Material and methods

### Genes and fly stocks

The sixteen Polycomb group (PcG) genes studied code for subunits of four Polycomb repressive complexes: PhoRC, Pcl-PRC2, PRC1 and dRAF. These genes are: *Pho*, its paralog *Phol* and *Sfmbt* (PhoRC complex); *Caf1-55*, *E(z)*, *Esc*, its paralog *Escl*, *Su(z)12* and *Pcl* (Pcl-PRC2 complex); *Psc*, *Sce*, *Pc*, *Ph-p*, its paralog *Ph-d* and *Scm* (PRC1 complex), and *Kdm2* (dRAF complex that also contains PSC and SCE). The sequences of these genes (except *Ph-d)* in *D*. *subobscura*, *D*. *madeirensis* and *D*. *guanche* have been previously reported [15] and are available in EMBL/GenBank Data Libraries. The location of these genes in the polytene chromosomes of the *chcu* strain of *D*. *subobscura* that is homokaryotypic for known chromosomal arrangements was determined by *in situ* hybridization using biotinylated probes, as described in [26]. The exact chromosomal section where the different probes hybridized was determined according to the Kunze-Mühl and Müller cytological map [27] that has the standard (or reference) arrangement for each chromosome.

Isofemale lines were established after sampling a natural population of *D*. *subobscura* in the Observatori Fabra (outskirts of Barcelona, Catalonia, Spain). Thereafter, individuals from these lines were used to obtain highly inbred lines by at least 12 generations of sib mating. After inbreeding, it was confirmed that lines were homokaryotypic and the gene arrangement of the different chromosomes was determined as described in [28].

The location of the genes in the chromosomes was considered to select the lines studied. This selection was made to ensure that the lines studied for each gene were homosoquential for the chromosomal region where the gene is located. This selection is important to prevent any effect of the inversion polymorphism on the level and pattern of nucleotide variation when a gene maps near the breakpoints of an inversion. For instance, *Ph-p* and *Ph-d* are located in the distal half of the A (= X) chromosome near one of the breakpoints of inversion 2, which is the single inversion present in the A_2_ arrangement. According to this location, lines A_2_ were excluded from the analysis of variation in these genes. Consequently, variation at *Ph-p* and *Ph-d* was studied in a random sample including only lines with the A_st_ and A_1_ arrangements that are homosequential for the chromosomal region were the genes map. S1 Table indicates the chromosome and chromosomal section where each gene is located and the arrangement for this chromosome of the selected lines. On average 15 lines were sequenced for each gene.

### DNA sequencing and analysis

Genomic DNA of the selected *D*. *subobscura* lines was purified with the Puregen^®^Core Kit B (Qiagen). The polymerase chain reaction was used to amplify the complete coding region of the PcG genes here studied and the obtained PCR products were purified with Multiscreen plates (Millipore). The ABI Prism BigDye Terminators 3.0 Cycle kit (Applied Biosystems) and internal primers were used to sequence completely both strands of the amplicons. The sequences of the primers used in the PCR amplification and sequencing are available on request to the authors. Sequencing reactions were run on an ABI PRISM 3700 sequencer at Serveis Científico-Tècnics de la Universitat de Barcelona. Partial sequences of each gene were assembled with the SEQMAN program of the DNASTAR v6.0 Lasergene package [29]. Complete sequences have been deposited in EMBL/GenBank Data Libraries under accession numbers LT856235 to LT856459. Finally, sequences were multiply aligned with the MEGA v6.0 software [30]. Nucleotide variation at *Caf1-55* in *D*. *subobscura* has been previously reported [31] and sequences are also available in EMBL/GenBank Data Libraries.

The DnaSP v5 program [32] was used to estimate the basic summary statistics that describe nucleotide variation, to measure the extent of codon bias and to perform most neutrality tests. The level and pattern of variation was estimated by the number of polymorphic sites (*S*), nucleotide diversity (*π*), haplotype diversity (*Hd*), Tajima’s *D* statistic [33], the minimum number of recombination events (*R*_*m*_) inferred by the four-gamete test [34] and the linkage disequilibrium between parsimony informative sites measured by the |*D’*| parameter [35]. Codon bias was estimated by the effective number of codons (ENC) as proposed in [36]. ENC ranges from 20 when a single codon is used for each amino acid (maximum codon bias) to 64 when all synonymous codons are used equally (no codon bias). Nucleotide diversity and divergence with *D*. *guanche* along each gene studied were also analyzed by the sliding window method using 50-nucleotide windows at five-nucleotide intervals with DnaSP v5.

Different neutrality tests were applied to the data to detect signatures of selection. The Tajima’s test [33] based only on polymorphism data and the different Fu and Li’s tests [37] were used to infer whether the observed pattern of variation conforms to that expected under the neutral model in a population at mutation-drift equilibrium. Fu and Li’s tests based on the D* and F* statistics can be performed when only polymorphism data are available, in contrast to those based on the *D* and *F* statistics that also need data on an outgroup species. *D*. *guanche* was used as outgroup to perform the neutrality tests that require interspecific data. Significance levels of Tajima’s *D* and Fu and Li’s *D* and *F* tests statistics were estimated according to the standard neutral model as implemented in DnaSP v5 program [32]. In addition, as *D*. *subobscura* has a genome wide pattern of variation consistent with an expansion process, significance levels of these tests statistics were estimated after 10000 computer simulations using the software mlcoalsim v1.42 [38] with the parameters of the expansion model inferred in [28]. In addition, the HKA test [39] and the MK test [40] were applied to detect a putative decoupling of polymorphism and divergence among genes (HKA test) and between synonymous and nonsynonymous sites within a gene (MK test). The multilocus HKA test was performed by the HKA program developed by J. Hey and available as a software resource at https://bio.cst.temple.edu/~hey. This program conducts coalescence simulations to infer the significance of the observed χ^2^-like test statistic and to perform the maximum cell value test that was proposed to detect significant outliers in the multilocus HKA test [41]. The test is based on the maximum absolute cell value among genes for the standardized discrepancies between observations and expectations detected for polymorphism when applying the multilocus HKA test. The null distribution of maximum cell values is inferred by computer simulations to assess the probability of the observed value.

## Results

**Fig 1:**
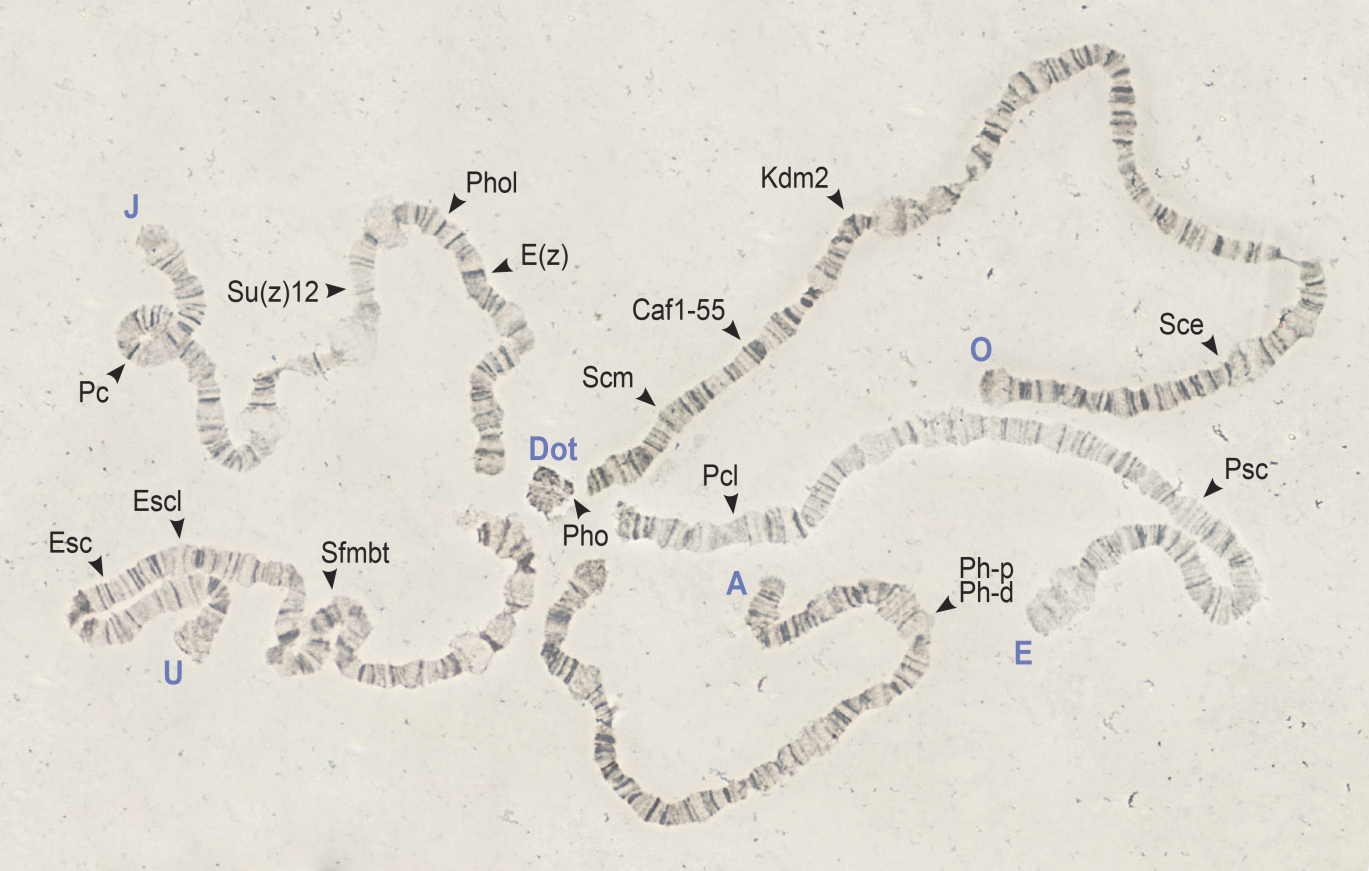
Location of the PcG genes on the *D*. *subobscura* chromosomes. The location of the sixteen studied PcG genes is indicated on the polytene chromosomes of *D*. *subobscura* (*ch cu* strain) by arrowheads. All chromosomes have the standard arrangement shown in the cytological map of the species [27] except the O chromosome that has the O_3+4_ arrangement that includes the overlapping inversions O_3_ and O_4_. Chromosomes are identified by capital letters (A, J, U, E and O) at the distal end. The dot-like chromosome is also indicated (Dot).

The chromosomal location in *D*. *subobscura* of the sixteen studied Polycomb group (PcG) genes is shown in Fig 1. These genes map at the expected chromosome according to their cytological location in *D*. *melanogaster* and the well-established chromosomal homologies between this species and *D*. *subobscura* [42]. Only the two tandem paralogs *Ph-p* and *Ph-d* are X-linked, all other genes are located in autosomes and *Pho* maps in the dot-like chromosome (Muller’s element F). Chromosomal locations were taken into account when selecting the chromosomal arrangement of the lines sequenced for each gene to prevent any effect of inversions on nucleotide variation (S1 Table).

Polymorphic sites and indels present in the multiple alignment of each gene are shown in S1 Fig. Summary statistics of nucleotide variation are shown in Table 1. Nucleotide diversity (*π*) ranges from 0.0009 at *Pho* to 0.0117 at *Escl*. Therefore, variation at *Pho* is about one order of magnitude lower than at *Escl*. The low level of nucleotide variation at *Pho* relative to the other PcG gene regions is also evident when nucleotide diversity is normalized by nucleotide divergence (*K*) with *D*. *guanche* to correct for differences in the mutation rate across gene regions. The *π/K* ratio at *Pho* is 0.0263 that is also the lowest estimate and about 3.2 times lower than the next value present at *Pcl* (0.0833). In addition to nucleotide diversity, *Pho* also has the lowest number of haplotypes (10 in 17 lines) and haplotype diversity (*Hd* = 0.9). No recombination events were inferred in the *Pho* region (*R*_*m*_ = 0) by the four-gamete test. *Pho* also present a rather low codon bias (ENC = 54.98). This estimate is slightly higher (indicating a lower codon bias) than the median ENC value (53.92) for the *D*. *melanogaster* genes located in the dot-like element [43]. Tajima’s *D* statistic was negative in all regions (Table 1), indicating an excess of low frequency variants at polymorphic sites in all genes. Negative Tajima’s *D* estimates are expected after the fixation of an advantageous mutation by positive selection (selective sweep). However, demographic factors as bottlenecks or population expansions can also cause the same pattern of variation. *D*. *subobscura* has a genome wide pattern of variation consistent with an expansion process [28] and, therefore, present results on the Polycomb genes are more likely explained by demographic than by selective factors. None of the performed Tajima’s *D* test was significant (*P* > 0.05) assuming either a constant population size or an expansion process. Fu and Li’s *D* and *F* tests using *D*. *guanche* as outgroup were significant at *Psc* (*P* = 0.0121 and *P* = 0.0212, respectively) and *Ph-d* (*P* = 0.0184 and *P* = 0.0187, respectively) under the unrealistic assumption of a constant population size but not significant under the expansion model (S2 Table), which indicates the importance of using an accurate demographic model to calculate the significance of the neutrality tests.

**Table 1:** Summary estimates of the level and pattern of polymorphism.

Complex *Gene*	n° lines	n° sites	*S*	*h*	*Hd*	*π*	*π/K*	Tajima's *D*	*R*_*m*_
PhoRC									
*Pho*	17	2842	11	10	0.90	0.0009	0.0263	-0.8763	0
*Sfmbt*	15	5617	76	14	0.99	0.0033	0.1055	-0.9055	9
*Phol*	16	2293	34	15	0.99	0.0030	0.1130	-1.3772	5
Pcl-PRC2									
*Caf1-55*	14	2621	85	13	0.99	0.0083	0.1857	-0.9447	9
*E(z)*	16	3652	143	15	0.99	0.0113	0.2162	-0.3766	16
*Esc*	15	2105	78	15	1.00	0.0098	0.3617	-0.7133	10
*Su(z)12*	16	3763	58	15	0.99	0.0039	0.1788	-0.7093	15
*Pcl*	16	3491	39	16	1.00	0.0027	0.0833	-0.8365	5
*Escl*	15	1756	66	14	0.99	0.0117	0.3400	-0.1027	10
PRC1									
*Psc*	15	11839	325	15	1.00	0.0060	0.1799	-1.3784	47
*Sce*	9	2714	50	9	1.00	0.0064	0.1455	-0.4042	6
*Pc*	17	3749	161	16	0.99	0.0085	0.2312	-1.4846	20
*Ph-p*	17	9203	176	16	0.99	0.0038	0.1348	-1.4433	20
*Scm*	16	4161	123	15	0.99	0.0060	0.1716	-1.4928	18
*Ph-d*	16	5752	63	15	0.99	0.0022	0.1007	-1.4728	6
dRAF*									
*Kdm2*	16	8507	186	15	0.99	0.0046	0.1403	-1.3780	20

*S*: number of segregating sites, *h*: number of haplotypes, *Hd*: haplotype diversity, *π*: nucleotide diversity per site, *π/K*: nucleotide diversity per site normalized by nucleotide divergence with *D*. *guanche*, *R*_*m*_: minimum number of recombination events.

*dRAF complex also contains PSC and SCE.

**Fig 2:**
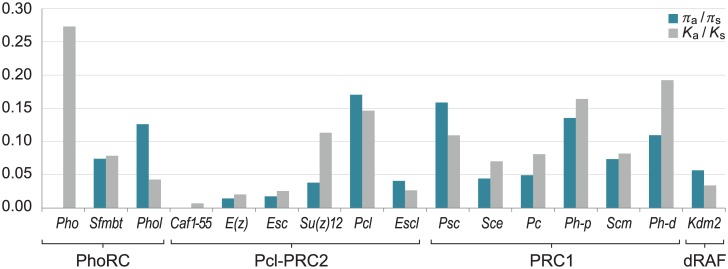
Polymorphism and divergence in the coding region of the PcG genes. Ratio of nonsynonymous to synonymous polymorphism in *D*. *subobscura* (*π*_*a*_/*π*_*s*_) and of nonsynonymous to synonymous divergence between *D*. *subobscura* and *D*. *guanche* (*K*_*a*_*/K*_*s*_) for each of the Polycomb genes studied. The *x*-axis indicates gene identity and the *y*-axis indicates *π*_*a*_/*π*_*s*_ and *K*_*a*_*/K*_*s*_ values. *Pho* lacks synonymous polymorphism and *Caf1-55* nonsynonymous polymorphism. Genes on the *x*-axis are grouped according to Polycomb complex. dRAF also contains PSC and SCE.

Nucleotide variation in the coding region was also analyzed independently for synonymous (*π*_*s*_) and nonsynonymous (*π*_*a*_) sites (Table 2). *Caf1-55* without nonsynonymous polymorphism and the lowest nonsynonymous divergence (*K*_*a*_) is the most conserved PcG gene. On the other hand, a lack of synonymous polymorphism is present at *Pho*. In fact, all polymorphic sites in the *Pho* coding region present nonsynonymous variants (S1 Fig). However, the overall nonsynonymous diversity at *Pho* is not high (*π*_*a*_ = 0.0006) given the low number of polymorphic sites (only 4). The highest nonsynonymous diversity is detected at *Psc* (*π*_*a*_ = 0.0019), which also has a high nonsynonymous divergence (*K*_*a*_ = 0.0097) only overcome by *Pho* (*K*_*a*_ = 0.0181) and *Pcl* (*K*_*a*_ = 0.0129).The ratios *π*_*a*_/*π*_*s*_ and *K*_*a*_*/K*_*s*_ in the different PcG genes are represented in Fig 2. These ratios are low when purifying selection against nonsynonymous mutations is strong. The genes encoding the core subunits of the PRC2 complex (*Caf1-55*, *E(z)*, *Esc* and *Su(z)12*) have the lowest *π*_*a*_/*π*_*s*_ ratios. In addition, *Caf1-55*, *E(z)* and *Esc* also have low *K*_*a*_*/K*_*s*_ ratios, which indicates a strong purifying selection acting on these genes according to both polymorphism and divergence. By contrast, *Pcl* and *Psc* have the two highest *π*_*a*_/*π*_*s*_ ratios and also a high *K*_*a*_*/K*_*s*_ ratio. PSC is a structurally disordered protein mainly in its C-terminal region [44], in which the distribution of the charged amino acids and not the sequence itself is important for protein function.

**Table 2:** Summary of synonymous and nonsynonymous polymorphism and divergence with *D*. *guanche* in the PcG genes.

Complex *Gene*	*π*_*s*_	*π*_*a*_	*π*_*a*_/*π*_*s*_	*K*_*s*_	*K*_*a*_	*K*_*a*_/*K*_*s*_
PhoRC						
*Pho*	0.0000	0.0006	-	0.0663	0.0181	0.2732
*Sfmbt*	0.0115	0.0009	0.0742	0.0890	0.0070	0.0786
*Phol*	0.0094	0.0012	0.1261	0.1051	0.0045	0.0428
Pcl-PRC2						
*Caf1-55*	0.0211	0.0000	0.0000	0.1417	0.0010	0.0070
*E(z)*	0.0242	0.0004	0.0145	0.1743	0.0036	0.0206
*Esc*	0.0234	0.0004	0.0176	0.0876	0.0022	0.0256
*Su(z)12*	0.0149	0.0006	0.0382	0.0621	0.0070	0.1133
*Pcl*	0.0071	0.0012	0.1704	0.0881	0.0129	0.1464
*Escl*	0.0334	0.0014	0.0407	0.1292	0.0034	0.0266
PRC1						
*Psc*	0.0120	0.0019	0.1586	0.0890	0.0097	0.1093
*Sce*	0.0149	0.0007	0.0443	0.0470	0.0033	0.0704
*Pc*	0.0128	0.0006	0.0494	0.0757	0.0061	0.0811
*Ph-p*	0.0070	0.0010	0.1355	0.0425	0.0070	0.1642
*Scm*	0.0137	0.0010	0.0736	0.0774	0.0063	0.0820
*Ph-d*	0.0056	0.0006	0.1097	0.0478	0.0092	0.1923
dRAF*						
*Kdm2*	0.0120	0.0006	0.0507	0.0901	0.0030	0.0334

*π*_*s*_: synonymous nucleotide diversity, *π*_*a*_: nonsynonymous nucleotide diversity, *π*_*a*_*/π*_*s*_: nonsynonymous/synonymous diversity ratio, *K*_*s*_: synonymous divergence, *K*_*a*_: nonsynonymous divergence, *K*_*a*_*/K*_*s*_: nonsynonymous/synonymous divergence ratio.

*dRAF complex also contains PSC and SCE.

Under strict neutrality, the ratio between intraspecific polymorphism and interspecific divergence is expected to be homogeneous across loci in a constant size population at mutation-drift equilibrium. Based on this expectation, the HKA neutrality test [39] was developed to detect a putative decoupling in the polymorphism to divergence ratio between two gene regions. Assuming that a region evolves under neutrality, a significant excess of polymorphism at the other region would indicate balancing selection. Conversely, a recent selective sweep would result in a deficit of polymorphism. The multilocus HKA test performed considering silent variation in the sixteen PcG genes was marginally significant using *D*. *guanche* as outgroup (χ^2^ = 22.09, df = 15, *P* = 0.0812). The HKA multilocus test is conservative when a single gene from a large sample present a ratio influenced by selection. A close inspection to the data indicates that this is the case for the PcG genes studied since *Pho* deviates considerably from the other genes (S2 Fig). In fact, *Pho* has the largest contribution (54%) to the overall χ^2^-like test statistic and the contribution of the other PcG genes is quite uniform and small. In addition, the absolute maximum discrepancies between expected and observed polymorphism in the χ^2^-like test statistic was detected at *Pho* (3.667) and is significant (*P* = 0.0129) using the maximum cell value test [41]. According to this result, the HKA test was applied in pairwise comparisons between *Pho* and each one of the other PcG genes. The 15 performed HKA tests were statistically significant (*P* < 0.05). This result clearly supports that *Pho* has a significant deficit of nucleotide polymorphism. Finally, the multilocus HKA test was not significant when excluding *Pho* from the analysis (χ^2^ = 9.96, df = 14, *P* = 0.7402).

The McDonald and Kreitman test [40] of neutrality (MK test) was also performed for each gene independently. This test is based on the comparison of the ratio of nonsynonymous to synonymous polymorphisms (*P*_*a*_/*P*_*s*_) and the ratio of nonsynonymous to synonymous fixed differences between species (*F*_*a*_/*F*_*s*_). Both ratios are expected to be equal under strict neutrality and a *G*-test of independence can be used to detect putative deviations from this expectation. A significant difference in both ratios was inferred only at *Su(z)12* (*G* = 4.909; 1 df, *P* = 0.0267). The direction of departure from neutrality can be inferred by the neutrality index (*NI*) estimated as the ratio between *P*_*a*_/*F*_*a*_ and *P*_*s*_/*F*_*s*_ [45]. Assuming that synonymous mutations are neutral, *NI* > 1 indicates an excess of nonsynonymous polymorphism and *NI* < 1 an excess of nonsynonymous divergence. The neutrality index at *Su(z)12* is 0.29, which indicates adaptive selection favouring nonsynonymous changes during the divergence of *D*. *subobscura* and *D*. *guanche* (see also Fig 2). The proportion of nonsynonymous substitutions fixed by natural selection at *Su(z)12* in these lineages is 0.71 according to the *α*-parameter [46]. The statistical power of the MK test when applied to *Pho* is affected by the low number of polymorphic sites, which might explain why the test is not significant despite the strong decoupling between polymorphism and divergence (Fig 2).

**Fig 3:**
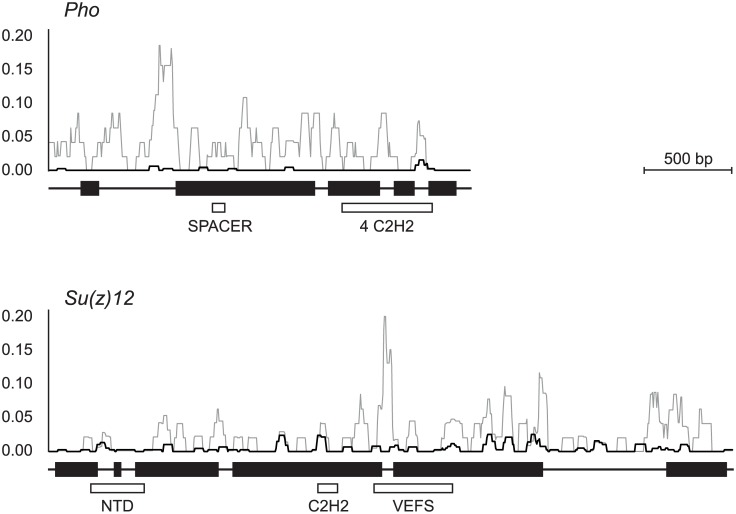
Nucleotide diversity and divergence along *Pho* and *Su(z)12*. Sliding window plots of the distribution of nucleotide diversity in *D*. *subobscura* (*π*, black line) and of nucleotide divergence between *D*. *subobscura* and *D*. *guanche* (*K*, gray line). Windows include 50 sites with successive displacements of 5 sites. The *x*-axis indicates nucleotide sites across the gene region and the *y*-axis indicates nucleotide diversity or divergence. Solid boxes in the lower part of the figure indicate the coding exons and thin lines show flanking regions and introns. The protein domains of the encoded proteins are indicated below the gene structure with empty boxes.

The distribution of nucleotide diversity and divergence along the sixteen Polycomb group genes was also analyzed by the sliding window approach. Fig 3 shows the results of this analysis in the two genes with a nonneutral variation: *Pho* and *Su(z)12*. The results for the other genes are shown in S3 Fig. Peaks of polymorphism at *Pho* are very low and mainly located in noncoding regions. The highest divergence peak at *Pho* is detected at the end of the first intron. In *Su(z)12* there is a rather good correspondence between peaks of polymorphism and divergence. The highest divergence peak is present in the short fourth intron. Divergence is also rather high at the end of exon 5 and in exon 6. The gene regions coding for protein domains are highly conserved in the two genes.

## Discussion

### Selection at the Polycomb group genes

Polycomb group (PcG) proteins form different complexes that maintain the repressive state of chromatin. The most relevant Polycomb complexes are Pho-RC, PRC1, Pcl-PRC2 and dRAF. The genes encoding the subunits of these complexes are mainly under the action of purifying selection as reflected by the *π*_*a*_/*π*_*s*_ and *K*_*a*_*/K*_*s*_ ratios (Fig 2). However, the strength of purifying selection differs both among the genes encoding the subunits of the same complex and among the genes encoding subunits of the different complexes. *Caf1-55*, *E(z)*, *Esc* and *Su(z)12* that code for the core subunits of the PRC2 complex are under a strong purifying selection and therefore highly conserved, except *Su(z)12* with a rather high *K*_*a*_*/K*_*s*_ ratio caused by positive selection (see below). PRC2 is a catalytic complex responsible for the histone H3 methylation at lysine 27, which is a chromatin repressive mark. The methyltransferase activity of PRC2 requires the E(Z) subunit with the active site for histone methylation and the noncatalytic subunits ESC and SU(Z)12 [47]. The critical role of the three subunits for the PRC2 function would explain their high conservation. PRC1 and dRAF are also catalytic complexes. The catalytic subunit of PRC1 is SCE that monoubiquitylates lysine 118 of histone H2A introducing a second chromatin silencing mark. *Sce* is also under strong purifying selection and it is the most conserved gene encoding subunits of the PRC1 complex. SCE is also a subunit of the dRAF complex that has an additional catalytic subunit that is KDM2. The gene encoding KDM2 (*Kdm2*) is also under strong purifying selection further confirming that the genes encoding the catalytic subunits of the Polycomb complexes are highly conserved.

Although most genes coding for the subunits of the Polycomb complexes are under puryfying selection, the footprint of adaptive selection was detected by the MK test in *Su(z)12* during the divergence of *D*. *subobscura* and *D*. *guanche*. SU(Z)12 is a noncatalytic subunit of the PRC2 complex that potentiates the enzymatic activity of its partner E(Z) and contributes to nucleosome binding [48,49]. The protein has three domains: an N-terminal domain, a zinc finger C2H2 and a VEFS box. The N-terminal domain is responsible for the interaction with CAF1-55 [50]. The VEFS domain enhances the methyltransferase activity of E(Z) and is critical for the SU(Z)12/E(Z) interaction [51]. Most of the fourteen amino acid replacements detected in the SU(Z)12 protein of *D*. *subobscura* and *D*. *guanche* are located at the poorly conserved C-terminal region of the protein (Fig 3) and none of them affect the described protein domains. Therefore, it is not expected that these replacements affect the interaction of SU(Z)12 with the other subunits of PRC2.

Polycomb proteins showed evidence of coevolution in a divergence study in the Drosophila genus [15]. In addition, a concordant acceleration of the evolutionary rate was detected in different PcG proteins mainly of the Pcl-PRC2 complex in the branch ancestral to the species of the *obscura* group. In contrast to this previous study, the present analysis of nucleotide variation in *D*. *subobscura* (as representative of the obscura group) in sixteen Polycomb groups genes failed to detect signs of coevolution and positive selection could only be inferred in *Su(z)12*. Coevolution between proteins is expected when amino replacements in the interacting interface of one protein are compensated by additional replacements in the interacting protein to maintain the integrity and function of the protein complex. The adaptive selection detected at *Su(z)12* during the divergence of *D*. *subobscura* and *D*. *guanche* is not evident in *Caf1-55* and *Esc*, which are two genes that code for subunits known to interact with SU(Z)12. The location of the amino acid replacement fixed between the two species outside the interacting interface of SU(Z)12 would explain the lack of coevolution with the partners of this protein.

In addition to *Su(z)12*, other genes involved in chromatin metabolism seem to have evolved under positive selection in *Drosophila*, such as *rhino* that belongs to the heterochromatin protein 1 (HP1) family [52]. In addition, genome wide analysis performed to detect gene regions with a strong genetic differentiation between tropical and temperate populations of *D*. *melanogaster* also identified chromatin organization genes such as *crm* [53], *Ph-p* [54], *chd1*, *ssrp*, *chm* and *glu* [55].

### Nucleotide variation at the dot-like chromosome of *D*. *subobscura*

The Polycomb gene *Pho* is located at the dot-like chromosome in *D*. *subobscura* near the distal or telomeric end of the chromosome. This position is shared by *D*. *melanogaster*, *D*. *yakuba*, *D*. *teissieri* and *D*. *erecta*, which supports that this is the ancestral location in the *Sophophora* subgenus. Therefore, the proximal location of *Pho* detected in other species of the *melanogaster* group such as *D*. *orena*, *D*. *mauritiana*, *D*. *simulans* and *D*. *sechellia* would be the result of chromosomal inversions as suggested in [20].

Nucleotide variation at *Pho* in *D*. *subobscura* when compared to other Polycomb genes is peculiar in several aspects: low nucleotide diversity, low number of haplotypes, rather low haplotype diversity and only nonsynonymous polymorphism in the coding region. In addition, no recombination events are detected by the four gamete test and thus linkage disequilibrium estimated by |*D’*| is equal to 1 in all pairwise comparisons between parsimony informative sites. Therefore, variation at *Pho* would support that the dot-like chromosome of *D*. *subobscura* also has an exceedingly low recombination rate as previously reported in other *Drosophila* species such as *D*. *melanogaster* [24]. This lack of recombination has a drastic effect on nucleotide variation. In fact, several multilocus studies have confirmed that the level and pattern of nucleotide variation differ between the dot-like chromosome and the other autosomes in *D*. *melanogaster* [21] and other *Drosophila* species not only of the *melanogaster* group such as *D*. *simulans* [22] and *D*. *yakuba* [23] but also of the *virilis* group as *D*. *americana* [56]. These studies show a low level of nucleotide polymorphism and relatively inefficient selection in the genes located in the dot-like chromosome. In addition, evidence of recombination at the molecular level has been detected in these multilocus studies of the dot-like chromosome although with a recombination rate much lower than in the other autosomes.

Mean nucleotide diversity in genes located in the dot-like chromosome is *π* = 0.0006 in *D*. *melanogaster* and *π* = 0.0009 in *D*. *simulans* [23]. These estimates are consistent with the level of variation at *Pho* in *D*. *subobscura* (*π* = 0.0009). The low level of variation in the dot-like chromosome is explained by both the hitchhiking effect [57] and the background selection model [58]. In fact, in regions with low recombination the fixation of positively selected mutations and the elimination of deleterious mutations cause a reduction of neutral linked variants. This reduction of variation can also be explained by the Hill-Robertson effect [59] that proposes that a locus linked to a second locus under directional selection experiences a reduction in effective population size (*N*_*e*_). The decrease in *N*_*e*_, apart from reducing neutral variation, causes a relaxation of selection due to an increase in the effects of genetic drift. The presence of only nonsynonymous polymorphism at the *Pho* gene of *D*. *subobscura* is consistent with a relaxation of selection against slightly deleterious nonsynonymous mutations. In addition, the low codon bias at *Pho* would indicate inefficient selection acting at synonymous sites to maintain the prevalent use of particular synonymous codons [60]. The relaxed selection might represent a challenge for the genes located in the dot-like chromosome, mainly for housekeeping genes under strong purifying selection. *Pho* and its paralog *Phol* are the only Polycomb group genes that bind directly to DNA. The PHO protein (or alternatively PHOL) binds to SFMBT and forms the PhoRC complex that is crucial for anchoring the other Polycomb complexes at the regulated target sites [47]. The high divergence of PHO in the Drosophila genus [15] and when comparing *D*. *subobscura* and *D*. *guanche* (Fig 3) suggests that this protein can accumulate amino acid replacements maintaining function as long as they do not affect the protein domains.

In summary, the level and pattern of nucleotide variation at the PcG genes in *D*. *subobscura* support the action of positive selection only at *Su(z)12*. The comparison of the ratio between polymorphic and fixed sites differs significantly for synonymous and nonsynonymous variants, suggesting adaptive evolution in this gene during the divergence of *D*. *subobscura* and *D*. *guanche*. In addition, the characteristics of the nucleotide polymorphism at *Pho* are consistent with the location of this gene in the dot-like chromosome and would indicate that this chromosome exhibits little or no recombination in *D*. *subobscura*.

## Supporting information

S1 Table Chromosomal location of the PcG genes. Location on the chromosomes of *D*. *subobscura* (A, J, U, E, O and Dot-like), chromosomal sections and chromosomal arrangements of the sequenced lines. The dot-like chromosome has not chromosomal polymorphism.
(PDF)

S2 Table Significance levels of the Tajima’s *D* and Fu and Li’s *D* and *F* tests statistics according to the expansion model. Each *P*-value was calculated after 10000 computer simulations using the software mlcoalsim v1.42 and the parameters obtained in Pratdesaba et al. (2015) indicated in the footnote.
(PDF)

S1 Fig Nucleotide polymorphic sites of the 16 PcG genes in the *D*. *subobscura* sequenced lines. The blue bar above each alignment illustrates gene structure: 5’ flanking region (5’), coding exons numbered by order (E), introns also numbered by order (I) and 3’ flanking region (3’). The identification of each line is given on the left by the letters OF (Observatori Fabra) and a number. Sites are numbered according to the multiple alignment. Asterisks under site numbers indicate nonsynonymous polymorphic sites. Dots indicate nucleotides identical to the first sequence that is used as reference. Gaps of a single nucleotide are shown by dashes. Insertions and deletions are indicated by the letters *i* and *d*, respectively, with a number according to its length. Polymorphic microsatellites are shown in brackets followed by a number that indicates the number of repeats. The last row of the multiple alignment shows the information for the polymorphic sites in the sequences of *D*. *guanche* (Dgua) used as outgroup. This information is also shown for the sequences of *D*. *madeirensi*s (Dmad), a closely relative to *D*. *subobscura*. A) *Pho*, B) *Sfmbt*, C) *Phol*, D) *Caf1-55*, E) *E(z)*, F) *Esc*, G) *Su(z)12*, H) *Pcl*, I) *Escl*, J) *Psc*, K) *Sce*, L) *Pc*, M) *Ph-p*, N) *Scm*, O) *Ph-d*, P) *Kdm2*.
(PDF)

S2 Fig Contribution of each PcG gene to the multilocus HKA test. Gray bars indicate the contribution to the overall χ^2^-like test statistic due to silent divergence between *D*. *subobscura* and *D*. *guanche*. Blue bars indicate the corresponding contribution due to silent polymorphism. The highest contribution of *Pho* both to divergence and polymorphism is significant by the maximum cell value test (see text).
(PDF)

S3 Fig Nucleotide diversity and divergence along PcG genes. Sliding window plots of the distribution of nucleotide diversity in *D*. *subobscura* (*π*, black line) and of nucleotide divergence between *D*. *subobscura* and *D*. *guanche* (*K*, gray line). Windows include 50 sites with successive displacements of 5 sites. The *x*-axis indicates nucleotide sites across the gene region and the *y*-axis indicates nucleotide diversity or divergence. Solid boxes in the lower part of the figure indicate the coding exons and thin lines show flanking regions and introns. *Pho* and *Su(z)12* plots are shown in Fig 3.
(PDF)
