# Supplementary material for: Molecular population genetics of the Polycomb genes in Drosophila subobscura
Source: PLoS One. 2017 Sep 14;12(9):e0185005. doi: 10.1371/journal.pone.0185005 (PMC5599051; doi:10.1371/journal.pone.0185005)
Supplement: S1 Table — Location on the chromosomes of D. subobscura (A, J, U, E, O and Dot-like), chromosomal sections and chromosomal arrangements of the sequenced lines. The dot-like chromosome has not chromosomal polymorphism. (PDF) [file pone.0185005.s001.pdf]

S1 Table. Chromosomal location of the PcG genes. Location on the chromosomes of *D. subobscura* (A, J, U, E, O and Dot-like), chromosomal sections and chromosomal arrangements of the sequenced lines. The dot-like chromosome has not chromosomal polymorphism.

Complex<br>*Gene*	Chromosome	Chromosomal section	Arrangements [a]
PhoRC			
Pho	Dot-like	100 D	-
Sfmbt	U	41 C	U_{1+2}
Phol	J	22 D	J_1
Pcl-PRC2			
Caf1-55	O	79 C	O_{st}, O_{3+4}, O_{3+4+8}
E(z)	J	21 C	J_{st}, J_1
Esc	U	49 A	U_{1+2}
Su(z)12	J	25 C	J_{st}, J_1
Pcl	E	56 B	E_{st}, E_{1+2}, $E_{1+2+9+12}$
Escl	U	47 B	U_{1+2}
PRC1			
Psc	E	66 C	E_{st}, E_{1+2}
Sce	O	96 A	O_{st}
Pc	J	33 A	J_{st}, J_1
Ph-p	A	13 A	A_{st}, A_1
Scm	O	77 D	O_{st}, O_{3+4}, O_{3+4+8}
Ph-d	A	13 A	A_{st}, A_1
dRAF			
Kdm2	O	82 B	O_{st}, O_{3+4}, O_{3+4+8}

[a] Standard arrangements are indicated by the letter of each chromosome and the subindex st. The subindex of a single arrangement indicates the inversion present in that arrangement. The different inversions present in a complex arrangement are linked by a + symbol.
