# Supplementary material for: Molecular population genetics of the Polycomb genes in Drosophila subobscura
Source: PLoS One. 2017 Sep 14;12(9):e0185005. doi: 10.1371/journal.pone.0185005 (PMC5599051; doi:10.1371/journal.pone.0185005)
Supplement: S2 Table — Each P-value was calculated after 10000 computer simulations using the software mlcoalsim v1.42 and the parameters obtained in Pratdesaba et al. (2015) indicated in the footnote. (PDF) [file pone.0185005.s002.pdf]

S2 Table. Significance levels of the Tajima's *D* and Fu and Li's *D* and *F* tests statistics according to the expansion model. Each *P*-value was calculated after 10000 computer simulations using the software mlcoalsim v1.42 and the parameters obtained in Pratdesaba et al. (2015) indicated in the footnote.

Complex<br>*Gene*	Tajima's *D*	Fu & Li's *D*	Fu & Li's *F*
PhoRC			
Pho	0.6376	0.0588	0.1228
Sfmbt	0.6860	0.2265	0.4706
Phol	0.5372	0.6368	0.9104
Pcl-PRC2			
Caf1-55	0.9967	0.8642	0.6513
E(z)	0.0770	0.2708	0.2406
Esc	0.4384	0.6767	0.8092
Su(z)12	0.4504	0.5766	0.7650
Pcl	0.6186	0.3117	0.5537
Escl	0.0722	0.1518	0.1270
PRC1			
Psc	0.3354	0.1700	0.0648
Sce	0.4126	0.3768	0.4304
Pc	0.4176	0.7619	0.4216
Ph-p	0.7238	0.7588	0.7130
Scm	0.4330	0.4928	0.2112
Ph-d	0.6078	0.5088	0.2066
dRAF			
Kdm2	0.4072	0.6039	0.2470

Parameters of the expansion model: time of occurrence of the population expansion ~128000 years ago (87415–180633, 95% HPD interval), current effective population size of *D. subobscura* ~1159107 individuals (7409106–1499107, 95% HPD interval) and ancestral population size ~7189105 individuals (2729105–1559106, 95% HPD interval).
