# Supplementary material for: Molecular population genetics of the Polycomb genes in Drosophila subobscura
Source: PLoS One. 2017 Sep 14;12(9):e0185005. doi: 10.1371/journal.pone.0185005 (PMC5599051; doi:10.1371/journal.pone.0185005)
Supplement: S1 Fig — The blue bar above each alignment illustrates gene structure: 5’ flanking region (5’), coding exons numbered by order (E), introns also numbered by order (I) and 3’ flanking region (3’). The identification of each line is given on the left by the letters OF (Observatori Fabra) and a number. Sites are numbered according to the multiple alignment. Asterisks under site numbers indicate nonsynonymous polymorphic sites. Dots indicate nucleotides identical to the first sequence that is used as reference. Gaps of a single nucleotide are shown by dashes. Insertions and deletions are indicated by the letters i and d, respectively, with a number according to its length. Polymorphic microsatellites are shown in brackets followed by a number that indicates the number of repeats. The last row of the multiple alignment shows the information for the polymorphic sites in the sequences of D. guanche (Dgua) used as outgroup. This information is also shown for the sequences of D. madeirensis (Dmad), a closely relative to D. subobscura. A) Pho, B) Sfmbt, C) Phol, D) Caf1-55, E) E(z), F) Esc, G) Su(z)12, H) Pcl, I) Escl, J) Psc, K) Sce, L) Pc, M) Ph-p, N) Scm, O) Ph-d, P) Kdm2. (PDF) [file pone.0185005.s003.pdf]

A) *Pho*

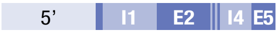

	127	252	303	369	537	838	1064	1140	1348	1509	1835	2581	2600	2640
									*	*	*			*
Chcu	A	T	T	-	C	G	A	A	A	G	G	G	A	A
OF01	.	.	.	-	.	.	C	.	G	.	.	.	.	.
OF07	.	.	.	-	.	.	C	G	.	.	.	.	.	.
OF14	.	.	.	-	.	.	C	.	G	.	.	.	.	.
OF18	.	.	.	-	.	.	C	.	.	.	.	.	.	.
OF19	.	.	.	-	.	.	.	.	.	.	A	.	.	.
OF21	.	.	.	-	.	.	C	.	.	.	.	.	.	.
OF23	G	.	C	-	.	.	C	.	.	.	.	T	T	.
OF28	G	.	.	-	.	.	C	.	.	.	.	T	T	.
OF31	.	.	.	-	.	.	C	.	.	.	.	.	.	.
OF40	G	.	.	-	T	.	C	.	.	.	.	T	T	.
OF47	G	.	.	-	.	d_2	C	.	.	.	.	T	T	.
OF49	.	.	.	-	.	.	C	.	.	T	.	.	.	.
OF54	.	.	.	-	.	.	C	.	.	.	.	.	.	.
OF58	.	.	.	-	.	.	C	.	.	.	.	.	.	T
OF60	.	.	.	-	.	.	.	.	.	.	A	.	.	.
OF78	.	d_4	.	i_4	.	.	C	.	.	.	.	.	.	.
Dmad	?	?	?	?	.	.	C	.	.	.	.	.	.	.
Dgua	?	?	?	?	.	.	C	-	.	.	.	.	.	.

S1 Fig. Nucleotide polymorphic sites of the 16 PcG genes in the *D. subobscura* sequenced lines. The blue bar above the alignment illustrates gene structure: 5’ flanking region (5’), coding exons numbered by order (E), introns also numbered by order (I) and 3’ flanking region (3’). The identification of each line is given on the left by the letters OF (Observatori Fabra) and a number. Sites are numbered according to the multiple alignment. Asterisks under site numbers indicate nonsynonymous polymorphic sites. Dots indicate nucleotides identical to the first sequence that is used as reference. Gaps of a single nucleotide are shown by dashes. Insertions and deletions are indicated by the letters *i* and *d*, respectively, with a number according to its length. Polymorphic microsatellites are shown in brackets followed by a number that indicates the number of repeats. The last row of the multiple alignment shows the information for the polymorphic sites in the sequences of *D. guanche* (Dgua) used as outgroup. This information is also shown for the sequences of *D. madeirensis* (Dmad), a closely relative to *D. subobscura*. A) *Pho*, B) *Sfmbt*, C) *Phol*, D) *Caf1-55*, E) *E(z)*, F) *Esc*, G) *Su(z)12*, H) *Pcl*, I) *Escl*, J) *Psc*, K) *Sce*, L) *Pc*, M) *Ph-p*, N) *Scm*, O) *Ph-d*, P) *Kdm2*.

B) *Sfmbt*

5' | E1 | I1 | E2 | I2 | E3 | E4 | I4 | E5 | I5 | E6 | I6 | E7 | 3'

	115	117	153	181	183	184	395	459	471	546	618	699	712	728	940	949	953	976	1065	1329	1339	1488	1516
OF01	T	G	A	G	G	A	A	A	A	A	C	A	G	A	-	C	C	C	G	A	-	G	$(GGC)_3$
OF07	.	.	T	.	.	.	.	C	.	G	.	G	.	.	-	.	.	.	.	C	i_3	C	$(GGC)_5$
OF13	.	.	T	.	.	.	.	C	.	G	.	.	.	.	-	.	.	.	.	.	-	C	$(GGC)_3$
OF14	.	.	T	.	.	.	C	C	.	.	.	G	.	.	-	.	.	.	.	.	-	C	$(GGC)_3$
OF15	.	.	T	.	.	.	.	C	.	G	.	G	.	.	-	.	.	.	.	C	i_3	C	$(GGC)_5$
OF18	.	.	T	.	.	.	.	C	.	G	.	G	.	.	-	.	.	.	.	C	i_3	C	$(GGC)_5$
OF19	.	T	T	.	.	.	.	C	.	.	.	.	.	.	-	.	.	.	.	.	-	C	$(GGC)_5$
OF21	.	.	.	.	.	.	.	C	.	.	.	.	.	.	-	.	A	T	.	C	-	C	$(GGC)_5$
OF23	G	.	T	.	.	.	.	C	.	.	A	G	.	.	A	.	.	.	.	.	-	C	$(GGC)_5$
OF31	.	.	T	.	.	.	.	C	.	G	.	.	A	.	-	.	.	.	.	.	-	C	$(GGC)_{10}$
OF42	.	.	T	.	.	.	.	C	.	G	.	.	.	.	-	.	A	.	.	C	-	C	$(GGC)_5$
OF47	.	.	T	.	.	.	.	C	T	.	.	.	.	C	-	G	.	.	C	.	-	C	$(GGC)_4$
OF49	.	.	T	T	T	T	.	C	.	G	.	G	.	.	-	.	.	.	.	.	-	C	$(GGC)_5$
OF60	.	T	T	.	.	.	.	C	.	.	.	.	.	.	-	.	.	.	.	.	-	C	$(GGC)_5$
OF74	.	.	T	.	.	.	.	C	.	.	.	G	.	.	-	.	.	.	.	.	-	C	$(GGC)_5$
Dmad	.	.	T	.	.	.	.	C	.	G	.	.	.	.	-	.	.	.	.	C	-	C	$(GGC)_4$
Dgua	.	.	T	.	.	.	.	G	.	G	A	.	.	.	-	.	.	.	.	C	-	C	$(GGC)_4$

	1568	1585	1687	1754	2077	2127	2128	2163	2331	2380	2496	2511	2529	2568	2589	2592	2613	2639	2644	2799	2844	2877	2886	3045
	*		*	*						*								*	*					
OF01	G	C	A	C	C	A	-	A	G	T	C	T	T	G	G	C	C	C	A	G	A	C	G	C
OF07	.	.	G	.	.	C	-	T	A	.	T	A	A	.	.	.	.	.	.	.	C	.	.	.
OF13	.	.	.	.	.	C	-	.	.	.	T	A	A	.	.	.	T	.	G	.	C	.	.	.
OF14	.	.	.	.	.	.	-	.	.	.	.	.	.	C	.	.	.	.	.	.	C	.	.	G
OF15	.	.	G	.	A	C	i_2	T	A	.	T	A	A	.	.	.	.	.	.	.	C	.	.	.
OF18	.	.	G	.	.	C	-	T	.	.	T	A	A	.	.	.	.	.	.	.	C	.	.	.
OF19	.	T	.	T	.	.	-	.	.	.	T	A	A	C	T	T	.	.	.	A	C	.	.	.
OF21	.	.	.	.	.	C	-	.	.	C	T	A	A	.	.	.	.	T	.	.	.	.	C	G
OF23	C	.	.	.	.	C	i_2	T	.	.	T	A	A	.	.	.	.	.	.	.	C	T	.	.
OF31	.	.	.	.	.	.	-	.	.	.	.	.	.	.	.	.	.	.	.	.	.	.	.	.
OF42	.	.	.	.	.	C	-	.	.	.	T	A	A	.	.	.	.	.	.	.	C	.	.	.
OF47	.	.	.	.	.	C	-	.	.	.	T	A	A	.	.	.	.	.	.	.	C	.	.	.
OF49	.	.	.	.	.	.	-	.	.	.	.	.	.	.	.	.	.	.	.	.	.	.	.	.
OF60	.	T	.	T	.	.	-	.	.	.	T	A	A	C	T	T	.	.	.	A	C	.	.	.
OF74	.	.	.	.	A	C	T	T	.	.	.	.	.	.	.	.	.	.	.	.	.	.	.	.
Dmad	.	.	.	.	A	C	-	T	.	.	T	.	.	C	.	T	.	.	.	.	C	.	.	G
Dgua	.	.	.	.	.	C	-	T	.	.	T	A	.	.	.	.	.	.	.	.	C	.	.	G

	3282	3423	3477	3570	3612	3633	3720	3747	3851	3902	3975	3980	4073	4151	4187	4323	4356	4359	4403
											*					*	*	*	
OF01	C	C	A	G	A	T	T	G	A	T	C	C	G	G	G	G	C	G	T
OF07	.	.	.	.	.	C	.	.	.	.	.	.	.	C	.	.	.	.	.
OF13	.	.	.	.	T	C	C	.	.	.	.	T	T	C	.	.	.	.	.
OF14	.	.	.	A	.	C	C	.	.	.	.	.	.	C	.	.	.	.	.
OF15	.	.	.	.	.	C	.	.	.	.	.	.	.	C	.	.	.	.	.
OF18	.	.	.	.	.	C	.	.	.	.	.	.	.	C	.	.	.	.	.
OF19	.	.	.	.	T	C	.	A	.	.	.	.	.	C	.	.	.	.	.
OF21	.	.	.	A	.	C	C	.	.	.	.	.	.	C	A	C	A	.	C
OF23	.	T	.	.	.	C	C	.	G	.	T	.	.	C	.	.	.	.	.
OF31	.	.	.	.	.	C	.	.	.	.	.	.	.	.	.	.	.	.	.
OF42	.	.	T	.	.	C	.	.	.	.	.	.	.	C	.	.	.	.	.
OF47	.	.	.	.	.	C	C	.	.	G	.	.	.	.	.	.	.	.	.
OF49	G	.	.	.	.	C	C	.	.	G	.	.	T	C	.	.	.	T	.
OF60	.	.	.	.	T	C	.	A	.	.	.	.	.	C	.	.	.	.	.
OF74	.	.	.	A	.	C	C	.	.	.	.	.	.	C	A	C	A	.	C
Dmad	.	.	G	.	.	C	.	.	.	.	.	.	.	C	.	C	.	.	.
Dgua	.	.	.	.	.	C	.	.	.	.	.	.	.	C	.	C	.	T	.

	4419	4452	4462	4560	4605	4721	4801	4860	4986	5043	5201	5327	5436	5483	5605	5632	5661
						*	*										
OF01	A	T	C	A	G	A	G	A	G	G	T	G	T	A	A	$(AC)_6$	C
OF07	G	.	A	G	.	.	A	.	.	.	.	.	.	.	C	$(AC)_{10}$	T
OF13	G	.	.	G	.	.	.	.	.	.	.	.	.	.	.	$(AC)_7$	T
OF14	G	.	A	G	.	.	A	.	.	.	.	.	C	.	T	$(AC)_7$	T
OF15	G	.	.	G	.	.	.	.	.	.	.	.	.	.	C	$(AC)_9$	.
OF18	G	.	A	G	.	.	A	.	.	.	.	.	C	.	T	$(AC)_7$	T
OF19	G	d_2	.	G	.	.	A	.	.	-	.	.	.	.	T	$(AC)_{10}$	T
OF21	G	.	.	G	.	.	A	.	.	.	.	.	.	.	.	$(AC)_{10}$	T
OF23	G	.	A	G	.	.	A	.	.	.	.	.	.	.	T	$(AC)_7$	T
OF31	.	.	.	.	.	.	.	.	.	.	.	.	.	.	.	$(AC)_{11}$	.
OF42	G	.	.	G	.	.	.	.	.	.	G	.	.	.	T	$(AC)_{11}$	.
OF47	G	.	.	G	T	.	.	T	A	.	.	T	.	.	.	$(AC)_9$	T
OF49	G	.	.	G	.	T	.	.	.	.	.	.	.	.	T	$(AC)_{11}$	.
OF60	G	d_2	.	G	.	.	A	.	.	-	.	.	.	.	T	$(AC)_{10}$	T
OF74	G	.	.	G	.	.	A	.	.	.	.	.	.	T	.	$(AC)_7$	T
Dmad	G	.	.	G	.	.	.	.	.	.	.	.	.	.	.	$(AC)_8$	?
Dgua	G	.	.	G	.	.	.	.	.	.	.	.	.	.	.	$(AC)_7$	?

C) *PhoI*

	5’	E1																																	
	10	123	151	265	293	427	616	625	637	842	913	934	1060	1065	1087	1195	1198	1237	1376	1385	1393	1569	1661	1663	1685	1689	1705	1715	1831	1898	2023	2110	2134	2203	2233
					*					*				*					*	*		*	*		*	*									
OF07	C	G	C	C	C	G	C	A	T	C	C	C	T	C	A	C	G	T	G	G	G	A	A	C	T	C	A	-	A	T	C	C	G	T	G
OF14	.	.	.	.	.	.	.	.	.	.	.	T	A	.	.	.	.	.	.	.	.	.	.	.	.	.	.	-	G	C	.	.	.	.	.
OF15	A	.	.	.	.	.	.	.	.	.	.	.	.	.	.	.	.	.	.	.	.	.	.	.	.	.	.	-	.	.	.	.	.	.	.
OF19	.	.	.	.	A	.	T	G	.	.	.	.	.	.	.	.	.	.	.	.	A	G	.	.	.	T	.	-	G	C	.	.	.	.	.
OF23	.	A	.	.	.	.	.	.	.	.	.	T	.	.	.	.	.	.	.	.	.	.	.	T	.	.	.	-	.	.	.	.	.	.	.
OF26	.	.	.	.	.	.	.	.	.	.	.	T	.	.	.	.	.	.	.	.	.	.	.	.	.	.	.	i_{12}	G	C	.	.	.	.	.
OF28	.	.	.	.	.	.	.	.	.	.	.	.	.	.	.	.	.	.	.	C	.	.	.	.	A	.	C	-	.	C	.	.	.	.	.
OF31	.	.	.	T	.	.	.	.	G	.	.	.	A	T	.	.	.	.	.	.	A	.	.	.	A	.	C	-	G	C	.	.	.	.	.
OF40	.	.	T	.	.	.	.	.	.	.	.	T	.	.	.	.	.	.	.	.	.	.	.	.	.	.	.	-	G	C	.	.	.	.	.
OF42	.	.	.	.	.	.	.	.	G	.	.	T	.	.	.	.	.	.	.	.	.	.	.	.	.	.	.	-	G	C	.	.	.	.	.
OF44	.	.	.	.	.	.	.	.	.	.	.	.	.	.	.	.	.	.	A	.	.	.	.	.	.	.	.	-	.	C	.	.	.	.	.
OF58	.	.	.	.	.	.	.	.	.	.	T	.	.	.	G	A	A	.	.	C	.	.	C	.	.	.	.	-	G	C	.	.	A	C	A
OF60	.	.	.	.	A	.	T	G	.	.	.	.	.	.	.	.	.	.	.	.	A	G	.	.	.	T	.	-	G	C	.	.	.	.	.
OF74	.	.	.	.	.	.	.	.	.	.	.	.	.	.	.	.	.	.	.	.	.	.	.	.	.	.	.	-	G	C	.	.	.	.	.
OF77	.	.	.	.	.	A	.	.	.	.	.	.	.	.	G	.	.	.	.	C	.	.	.	.	.	T	.	-	G	C	.	.	.	.	.
OF78	.	.	.	.	.	.	.	.	G	A	.	.	.	.	.	.	.	C	.	C	.	.	.	.	.	.	.	-	G	C	T	T	.	.	.
Dmad	.	.	.	.	.	.	T	G	.	.	.	.	.	.	G	A	A	.	.	C	.	.	.	.	A	.	C	-	G	C	.	.	.	.	.
Dgua	.	.	.	.	.	.	.	.	.	.	.	.	.	.	.	A	A	.	.	C	.	.	.	.	A	.	.	-	G	C	.	.	.	.	.

D) *Caf1-55*

5’ | I1 | E2 | I2 | E3 | 3’

	34	55	75	88	123	139	142	145	155	172	180	195	220	244	247	273	284	304	314	329	346	348	361
OF01	C	G	C	G	T	A	C	A	G	T	A	A	A	C	C	C	G	A	T	C	C	-	A
OF07	.	.	.	.	.	.	A	d_8	.	G	.	.	C	.	.	T	.	T	.	.	.	i_3	.
OF14	.	.	.	.	.	.	A	d_8	.	G	.	.	C	.	.	T	.	T	.	.	.	i_3	.
OF16	T	A	.	.	.	.	.	.	.	G	.	T	.	.	.	.	.	.	.	.	.	-	.
OF18	.	.	T	.	.	G	A	.	.	G	.	.	C	.	.	T	.	T	.	.	.	i_3	.
OF19	.	.	.	T	.	.	A	.	.	G	.	.	C	.	.	T	.	T	.	A	.	i_3	.
OF28	T	A	.	.	.	.	A	.	.	G	.	T	.	.	.	.	.	.	.	.	.	-	.
OF31	.	.	.	.	A	.	A	.	A	G	T	.	.	A	T	T	T	T	C	.	T	-	T
OF44	T	A	.	.	.	.	A	.	.	G	.	T	.	.	.	.	.	.	.	.	.	-	.
OF47	.	.	.	.	.	.	A	d_8	.	G	.	.	C	.	.	T	.	T	.	.	.	i_3	.
OF54	.	.	.	.	.	.	A	d_8	.	G	.	.	C	.	.	T	.	T	.	.	.	i_3	.
OF58	.	.	.	.	.	.	A	d_8	.	G	.	.	C	.	.	T	.	T	.	.	.	i_3	.
OF60	.	.	.	T	.	.	A	.	.	G	.	.	C	.	.	T	.	T	.	A	.	i_3	.
OF78	.	.	.	.	.	.	A	.	.	G	.	.	C	.	.	T	.	T	.	.	.	i_3	.
Dmad	.	A	.	.	.	.	A	.	.	.	.	.	.	.	A	T	.	T	.	.	.	-	T
Dgua	?	?	?	?	?	?	?	?	?	?	?	?	?	?	?	?	?	?	?	?	?	?	?

	372	374	409	411	414	434	435	438	446	475	478	479	483	494	535	537	571	579	602
OF01	A	C	C	C	A	T	C	C	C	G	A	T	T	G	C	T	G	C	$(A)_{12}$
OF07	.	.	A	A	d_3	A	.	.	.	.	.	A	.	.	.	.	.	A	$(A)_{12}$
OF14	.	.	A	A	d_3	A	.	.	.	.	.	A	.	.	.	.	.	A	$(A)_{12}$
OF16	.	.	.	.	.	G	.	.	.	A	G	A	G	.	G	C	.	A	$(A)_{15}$
OF18	.	A	A	A	.	A	.	.	G	.	.	A	.	.	.	.	A	.	$(A)_{12}$
OF19	.	.	A	A	d_3	A	.	.	.	.	.	A	.	.	.	.	.	A	$(A)_{12}$
OF28	.	.	.	.	.	G	T	-	.	.	G	A	C	.	G	C	.	A	$(A)_{16}$
OF31	.	.	A	A	.	A	.	T	.	.	.	A	.	A	.	.	.	A	$(A)_{15}$
OF44	.	.	.	.	.	G	.	.	.	.	G	A	G	.	G	C	.	A	$(A)_{14}$
OF47	.	.	A	A	d_3	A	.	.	.	.	.	A	.	.	.	.	.	A	$(A)_{12}$
OF54	.	.	A	A	d_3	A	.	.	.	.	.	A	.	.	.	.	.	A	$(A)_{12}$
OF58	.	.	A	A	d_3	A	.	.	.	.	.	A	.	.	.	.	.	A	$(A)_{12}$
OF60	.	.	A	A	d_3	A	.	.	.	.	.	A	.	.	.	.	.	A	$(A)_{12}$
OF78	T	.	A	A	d_3	A	.	.	.	.	.	A	.	.	.	.	.	A	$(A)_{12}$
Dmad	.	.	A	A	.	A	.	.	.	.	G	A	.	.	G	C	.	A	$(A)_{12}$
Dgua	?	?	?	?	?	?	?	?	?	?	?	?	?	?	?	?	?	?	$(A)_{10}$

	640	641	660	687	739	767	812	818	832	919	960	968	1150	1294	1375	1456	1535	1543	1546	1572	1614	1623	1629
OF01	T	-	A	A	A	A	G	A	C	G	T	C	G	G	C	A	G	G	G	T	C	T	T
OF07	.	-	.	C	.	.	.	.	.	.	.	.	.	.	T	C	.	T	.	.	T	C	C
OF14	.	-	.	C	.	.	.	.	.	.	.	.	.	.	.	.	.	.	.	.	.	.	.
OF16	C	A	G	.	G	T	.	G	.	.	.	.	T	.	T	C	.	.	.	.	T	.	C
OF18	.	-	.	.	.	.	.	.	.	.	.	.	.	A	.	C	.	.	.	C	T	.	C
OF19	.	-	.	C	.	.	.	.	.	.	.	.	.	.	T	C	A	.	C	.	.	.	.
OF28	C	A	G	.	G	T	.	G	.	.	.	A	.	.	T	C	.	.	.	.	T	C	C
OF31	.	-	.	.	.	.	C	.	.	A	.	A	.	.	T	C	.	.	.	.	T	.	C
OF44	C	A	G	.	G	T	.	G	d_9	.	.	.	.	.	T	C	.	.	.	.	T	.	C
OF47	.	-	.	C	.	.	.	.	.	.	C	.	.	.	.	.	.	.	.	.	.	.	.
OF54	.	-	.	C	.	.	.	.	.	.	.	.	.	.	.	.	.	.	.	.	T	.	C
OF58	.	-	.	C	.	.	.	.	.	.	.	.	.	.	.	.	.	.	.	.	.	.	.
OF60	.	-	.	C	.	.	.	.	.	.	.	.	.	.	T	C	A	.	C	.	.	.	.
OF78	.	-	.	C	.	.	.	.	.	.	.	.	.	.	.	C	.	.	.	.	T	.	C
Dmad	C	A	G	.	G	.	C	.	.	A	.	.	.	.	.	T	.	.	.	A	.	.	.
Dgua	.	A	G	.	G	.	.	.	.	.	.	G	T	.	.	C	A	.	T	.	.	.	.

	1686	1752	1896	1951	1980	1986	2004	2013	2016	2028	2067	2082	2271	2298	2350	2358	2413	2453	2461	2465	2490	2497	2509	2510	2551	2557	2611	2618
OF01	C	C	A	T	G	C	C	G	C	C	G	A	C	C	T	T	T	G	A	A	T	-	C	C	C	G	C	A
OF07	.	T	G	.	.	.	.	.	.	T	.	.	.	.	.	C	.	.	.	.	.	-	.	.	.	C	T	T
OF14	.	.	.	.	.	.	.	.	.	.	.	.	.	.	.	C	A	.	.	.	C	-	.	.	T	C	.	.
OF16	.	.	G	.	.	T	.	.	.	.	.	G	.	.	.	C	A	.	.	.	.	-	.	.	T	C	.	.
OF18	.	.	G	.	.	T	.	.	.	.	.	G	.	.	.	.	.	.	.	.	.	-	G	.	T	C	.	.
OF19	.	.	.	.	.	.	.	.	.	.	.	.	A	.	.	C	A	.	.	.	.	-	.	.	.	C	.	.
OF28	T	T	G	C	A	.	.	.	.	.	.	G	.	.	.	C	A	.	.	.	.	-	.	.	.	C	.	.
OF31	.	.	G	.	.	.	.	.	A	.	.	.	.	T	.	C	A	.	.	.	.	-	.	.	.	C	.	.
OF44	.	.	.	.	.	.	.	A	.	.	.	G	.	.	.	C	A	.	.	.	.	-	.	.	T	C	.	.
OF47	.	.	.	.	.	.	.	.	.	.	.	.	A	.	.	C	A	.	.	.	.	-	.	.	.	C	.	.
OF54	.	.	G	.	.	.	T	.	.	.	.	.	.	.	.	C	A	A	.	.	.	A	.	G	T	C	.	.
OF58	.	.	.	.	.	T	.	.	.	.	.	.	.	.	.	C	.	.	G	T	.	-	.	.	.	C	.	.
OF60	.	.	.	.	.	.	.	.	.	.	.	.	A	.	.	C	A	.	.	.	.	-	.	.	.	C	.	.
OF78	.	.	.	.	.	.	.	.	.	.	A	.	.	.	G	C	A	.	.	.	.	-	.	.	.	C	.	.
Dmad	.	T	G	C	.	.	.	.	.	.	.	.	A	.	.	C	.	.	.	.	.	-	.	.	.	C	.	.
Dgua	.	T	G	.	.	.	G	.	.	.	.	.	A	.	.	C	.	.	.	.	C	-	.	.	.	C	.	.

E) *E(z)*

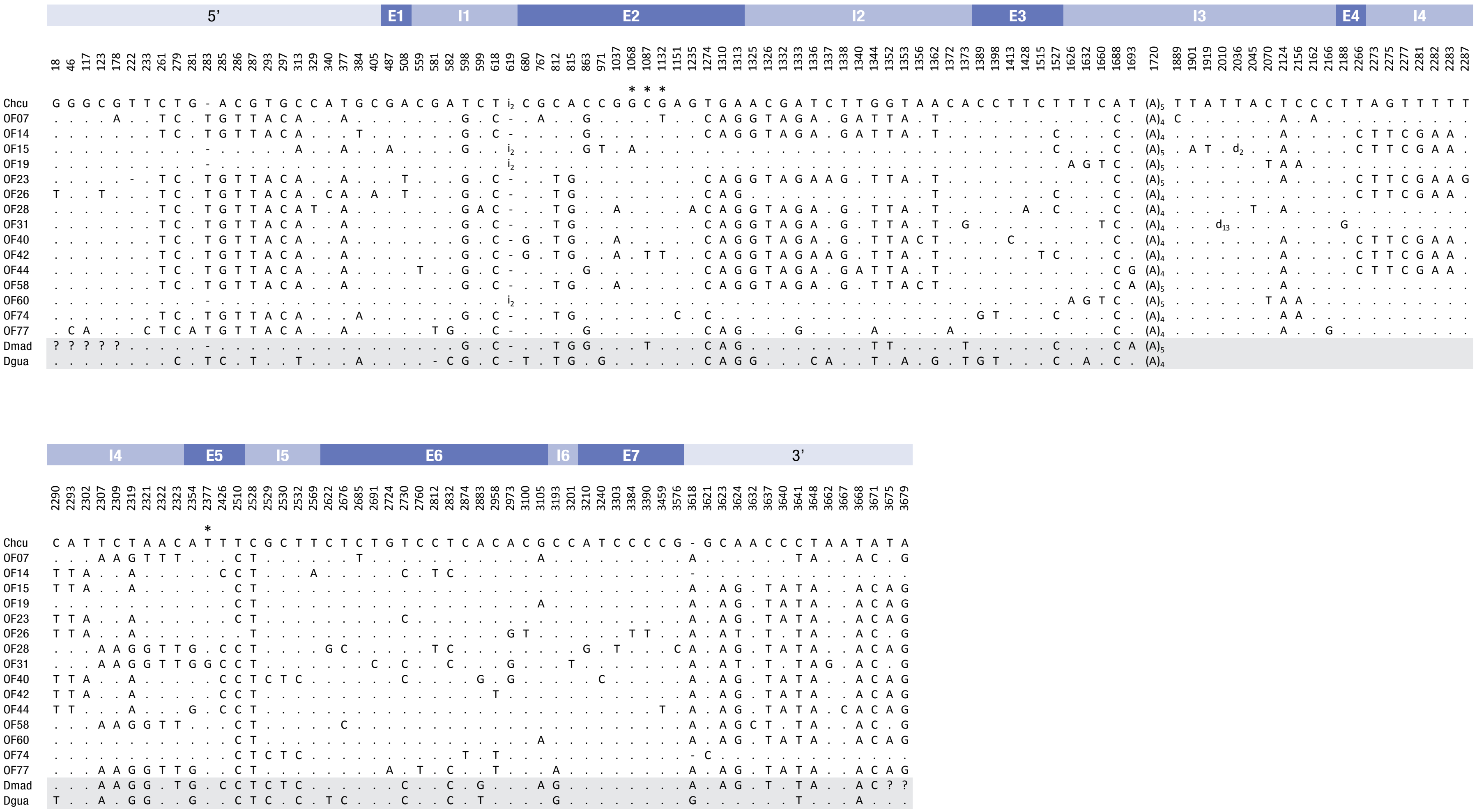

5' E1 I1 E2 I2 E3 I3 E4 I4
18 46 117 123 178 222 233 261 279 281 283 285 286 287 293 297 313 329 340 377 384 405 487 508 559 581 582 598 599 618 619 680 767 812 815 863 971 1037 1068 1087 1132 1151 1235 1274 1310 1313 1325 1326 1332 1333 1336 1337 1338 1340 1344 1352 1353 1356 1362 1372 1373 1389 1398 1413 1428 1515 1527 1626 1632 1660 1688 1693 1720 1889 1901 1919 2010 2036 2045 2070 2124 2156 2162 2166 2188 2266 2273 2275 2277 2281 2282 2283 2287
* * *
Chcu G G G C G T T C T G - A C G T G C C A T G C G A C G A T C T i_2 C G C A C C G G C G A G T G A A C G A T C T T G G T A A C A C C T T C T T T C A T $(A)_5$ T T A T T A C T C C C T T A G T T T T T
OF07 . . . . A . . T C . T G T T A C A . . A . . . . G . C - . A . . G . . . . T . . C A G G T A G A . G A T T A . T . . . . C . $(A)_4$ C . . . . . . A . A . . . .
OF14 . . . . T C . T G T T A C A . . . T . . . . . . G . C - . . . . G . . . . C A G G T A G A . G A T T A . T . . . . C . . . C . $(A)_4$ . . . . A . . . . C T T C G A A .
OF15 . . . . - . . . . . A . . A . . A . . . . G . . i_2 . . . . G T . A . . . . C . . . C . $(A)_5$. A T . d_2 . . A . . . . C T T C G A A .
OF19 . . . . - . . . . i_2 . . . . A G T C . $(A)_5$ . . . . . . T A A . . . .
OF23 . . . . . - . T C . T G T T A C A . . A . . . T . . . G . C - . . T G . . . . C A G G T A G A A G . T T A . T . . . . C . $(A)_5$ . . . . A . . . . C T T C G A A G
OF26 T . . T . . . T C . T G T T A C A . C A . A . T . . . G . C - . . T G . . . . C A G . . . . T . . . . C . . . C . $(A)_4$ . . . . C T T C G A A .
OF28 . . . . T C . T G T T A C A T . A . . . . G A C - . . T G . . A . . . . A C A G G T A G A . G . T T A . T . . . . . A . C . . . C . $(A)_4$ . . . . . T . A . . . .
OF31 . . . . T C . T G T T A C A . . A . . . . G . C - . . T G . . . . C A G G T A G A . G . T T A . T . G . . . . T C . $(A)_4$ . . . d_{13} . . . . G . . . .
OF40 . . . . T C . T G T T A C A . . A . . . . G . C - G . T G . . A . . . . C A G G T A G A . G . T T A C T . . . C . . . . C . $(A)_4$ . . . . A . . . . C T T C G A A .
OF42 . . . . T C . T G T T A C A . . A . . . . G . C - G . T G . . A . T T . C A G G T A G A A G . T T A . T . . . . . . T C . . . C . $(A)_4$ . . . . A . . . . C T T C G A A .
OF44 . . . . T C . T G T T A C A . . A . . . . T . . G . C - . . . . G . . . . C A G G T A G A . G A T T A . T . . . . C G $(A)_4$ . . . . A . . . . C T T C G A A .
OF58 . . . . T C . T G T T A C A . . A . . . . G . C - . . T G . . A . . . . C A G G T A G A . G . T T A C T . . . . C A $(A)_5$ . . . . A . . . .
OF60 . . . . - . . . . i_2 . . . . A G T C . $(A)_5$ . . . . . . T A A . . . .
OF74 . . . . T C . T G T T A C A . . . A . . . . . . G . C - . . T G . . . . . C . C . . . . G T . . C . . . C . $(A)_4$ . . . . A A . . . .
OF77 . C A . . . C T C A T G T T A C A . . A . . . . . T G . C - . . . . G . . . . C A G . . . G . . . . A . . . . A . . . . C . $(A)_4$ . . . . A . . G . . . .
Dmad ? ? ? ? ? . . . . . - . . . . G . C - . . T G G . . . T . . . C A G . . . . T T . . . T . . . . . C . . . C A $(A)_5$
Dgua . . . . C . T C . T . . . T . . . A . . . . - C G . C - T . T G . G . . . . . . C A G G . . . C A . . T . A . G . T G T . . . C . A . C . $(A)_4$
I4 E5 I5 E6 I6 E7 3'
2290 2293 2302 2307 2309 2319 2321 2322 2323 2354 2377 2426 2510 2528 2529 2530 2532 2569 2622 2676 2685 2691 2724 2730 2760 2812 2832 2874 2883 2958 2973 3100 3105 3193 3201 3210 3240 3303 3384 3390 3459 3576 3618 3621 3623 3624 3632 3637 3640 3641 3648 3662 3667 3668 3671 3675 3679
*
Chcu C A T T C T A A C A T T T C G C T T C T C T G T C C T C A C A C G C C A T C C C C G - G C A A C C C T A A T A T A
OF07 . . . A A G T T T . . . C T . . . . . . T . . . . A . . . . A . . . . . . T A . . A C . G
OF14 T T A . . A . . . . . C C T . . . A . . . . . C . T C . . . . - . . . .
OF15 T T A . . A . . . . . . C T . . . . A . A G . T A T A . . A C A G
OF19 . . . . C T . . . . A . . . . A . A G . T A T A . . A C A G
OF23 T T A . . A . . . . . . C T . . . . C . . . . A . A G . T A T A . . A C A G
OF26 T T A . . A . . . . T . . . . G T . . . . T T . A . A T . T . T A . . A C . G
OF28 . . . A A G G T T G . C C T . . . . G C . . . . . T C . . . . G . T . . . C A . A G . T A T A . . A C A G
OF31 . . . A A G G T T G G C C T . . . . C . C . . C . . . G . . . T . . . . A . A T . T . T A G . A C . G
OF40 T T A . . A . . . . . C C T C T C . . . . . . C . . . . G . G . . . . . . C . . . . A . A G . T A T A . . A C A G
OF42 T T A . . A . . . . . C C T . . . . T . . . . A . A G . T A T A . . A C A G
OF44 T T . . . A . . . G . C C T . . . . T . A . A G . T A T A . C A C A G
OF58 . . . A A G G T T . . . C T . . . . . . C . . . . A . A G C T . T A . . A C . G
OF60 . . . . C T . . . . A . . . . A . A G . T A T A . . A C A G
OF74 . . . . C T C T C . . . . T . T . . . . - C . . . .
OF77 . . . A A G G T T G . . C T . . . . A . T . C . . T . . . A . . . . A . A G . T A T A . . A C A G
Dmad . . . A A G G . T G . C C T C T C . . . . . . C . . C . G . . . A G . . . . A . A G . T . T A . . A C ? ?
Dgua T . . A . G G . . G . . C T C . C . T C . . . C . . C . T . . . . G . . . . G . . . . . . T . . . A . . .

F) *Esc*

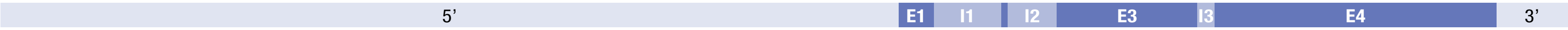

	5	10	25	56	62	63	67	71	72	75	79	80	84	115	165	166	185	191	195	205	210	214	216	227	243	250	262	266
OF01	C	A	T	T	T	C	$(C)_4$	T	C	C	T	C	T	G	T	T	C	A	G	T	T	T	T	-	A	C	-	T
OF07	.	T	.	C	.	.	$(C)_3$	.	.	T	.	A	.	.	.	A	T	.	.	G	A	A	.	-	C	.	-	A
OF13	.	.	.	C	.	.	$(C)_3$	.	.	.	C	.	.	.	.	A	T	.	.	G	.	.	C	A	C	T	-	G
OF14	.	T	.	C	.	.	$(C)_3$	.	.	.	C	.	.	.	.	A	T	.	T	G	A	A	.	-	C	.	T	G
OF15	T	T	.	C	.	.	$(C)_1$	C	.	T	C	.	-	.	.	A	T	.	.	G	.	.	C	-	C	.	T	G
OF18	.	.	.	.	.	.	$(C)_4$	.	.	.	.	.	.	.	.	.	.	.	.	.	.	.	.	-	C	.	-	.
OF19	.	T	.	C	.	A	$(C)_1$	C	.	T	C	.	-	.	.	A	T	.	.	G	A	A	.	-	C	.	-	A
OF21	T	T	.	C	.	.	$(C)_1$	C	T	T	C	.	-	.	C	A	T	C	.	G	.	.	C	-	C	T	-	.
OF23	.	.	d_{13}	C	.	.	$(C)_4$	.	.	.	.	.	.	.	.	.	.	.	.	G	.	A	.	-	C	T	-	A
OF31	T	T	.	C	.	.	$(C)_1$	C	T	T	C	.	-	.	.	A	T	C	.	G	.	.	C	-	C	.	T	G
OF42	.	T	.	C	.	.	$(C)_1$	C	.	T	C	.	-	A	.	A	T	.	.	G	.	.	C	-	C	T	-	G
OF47	.	T	.	C	A	.	$(C)_1$	C	.	T	C	.	-	.	.	A	T	.	.	G	.	.	C	-	C	T	-	G
OF49	.	.	.	.	.	.	$(C)_4$	.	.	.	.	.	.	.	.	.	.	.	.	.	.	.	.	-	C	.	-	.
OF60	.	T	.	C	.	.	$(C)_1$	C	.	T	C	.	-	.	.	A	T	.	.	G	.	.	C	-	C	T	-	G
OF74	.	T	.	C	.	.	$(C)_3$	.	.	T	.	.	.	.	.	A	T	.	.	.	.	.	.	-	C	T	-	G
Dmad	.	T	.	C	.	T	$(C)_3$	.	T	.	C	.	-	.	.	A	T	.	.	G	.	-	.	-	C	.	-	G
Dgua	.	T	.	-	.	.	$(C)_3$	.	.	.	A	.	-	.	G	A	T	.	.	G	.	.	.	-	C	.	-	G

	270	272	279	284	291	297	298	301	318	351	378	430	445	446	447	479	482	490	492	536	542	568	588	621	638	657	661
																						*	*				
OF01	A	A	A	C	T	C	G	T	C	T	A	A	T	A	$(A)_9$	C	G	T	G	T	A	A	T	T	G	T	C
OF07	.	.	.	.	.	T	.	.	.	.	G	T	.	.	$(A)_9$	.	.	.	.	.	.	.	.	.	.	.	.
OF13	T	C	C	.	.	T	.	.	.	C	G	T	.	.	$(A)_9$	A	A	.	.	.	.	.	.	.	.	.	.
OF14	.	C	.	.	d_2	T	.	.	.	C	G	T	.	.	$(A)_8$	A	.	.	.	.	.	.	.	G	.	.	.
OF15	.	C	.	.	d_2	T	.	.	.	C	G	T	.	T	$(A)_8$	A	.	.	.	.	.	.	.	.	.	.	.
OF18	.	.	.	G	.	.	.	.	.	.	G	T	.	.	$(A)_9$	.	.	.	.	.	.	.	.	.	.	.	.
OF19	.	.	.	.	.	T	.	.	A	.	G	T	.	.	$(A)_9$	.	.	.	.	.	.	.	.	.	.	.	.
OF21	T	C	.	.	.	T	.	.	.	.	G	T	.	.	$(A)_9$	A	.	.	.	.	.	.	.	.	.	.	.
OF23	.	.	.	.	.	T	.	.	.	.	G	T	.	.	$(A)_9$	A	.	.	.	.	.	.	.	.	.	.	.
OF31	.	C	.	.	d_2	T	.	.	.	C	G	T	.	.	$(A)_9$	A	.	.	.	.	.	.	.	.	.	.	.
OF42	T	C	.	.	.	T	.	.	.	C	G	T	.	.	$(A)_6$	A	.	.	.	C	T	T	.	.	.	C	T
OF47	T	C	.	.	.	T	A	A	.	C	G	T	.	.	$(A)_9$	A	.	.	.	.	.	.	.	.	.	.	.
OF49	.	.	.	.	.	T	.	.	.	.	G	T	.	.	$(A)_9$	.	.	.	.	.	.	.	.	.	.	.	.
OF60	T	C	.	.	.	T	A	.	.	C	G	T	A	.	$(A)_9$	A	.	.	.	.	.	.	A	.	A	.	.
OF74	.	C	.	.	d_2	T	.	.	.	C	G	T	.	.	$(A)_9$	A	.	A	C	.	.	.	.	.	.	.	.
Dmad	T	C	.	.	.	T	.	.	.	C	G	T	.	.	$(A)_8$	A	.	.	.	.	.	.	.	.	.	.	T
Dgua	T	C	.	.	.	T	.	.	.	C	G	T	.	.	$(A)_{11}$	A	.	.	.	C	.	.	.	.	.	.	T

	828	844	856	891	915	921	951	1005	1020	1026	1038	1095	1163	1166	1187	1304	1394	1433	1595	1685	1745	1802	1847	1895	1931	1947	1967	1970	2024	2026	2065	2117
																										*						
OF01	T	A	A	C	A	C	A	G	T	C	T	A	G	G	T	C	G	T	G	G	T	A	G	C	T	A	T	T	A	A	A	-
OF07	C	.	.	.	G	.	G	.	G	T	C	.	.	.	A	.	.	.	.	.	.	.	.	.	C	.	.	.	-	C	.	-
OF13	.	.	.	.	G	.	.	.	G	T	C	.	.	.	A	.	.	.	.	T	C	.	.	.	C	.	C	.	.	.	.	T
OF14	.	.	C	.	G	.	G	.	G	.	.	.	.	T	A	.	.	A	.	.	.	.	.	T	C	.	.	.	.	.	.	T
OF15	.	G	.	.	G	.	G	.	G	.	.	.	.	T	A	.	.	.	C	.	.	.	.	.	C	G	.	.	.	.	.	T
OF18	.	.	.	.	.	.	.	.	.	.	.	.	.	.	.	.	.	.	.	T	.	G	.	.	C	.	C	.	.	.	.	-
OF19	.	.	.	.	G	.	G	.	G	T	C	.	.	.	.	.	.	.	.	.	C	.	.	.	C	.	C	C	.	.	.	T
OF21	.	.	.	.	G	.	G	.	G	.	.	.	.	.	.	.	.	.	C	.	.	G	.	.	C	.	C	.	.	.	.	-
OF23	.	.	.	.	G	.	.	A	G	.	.	.	T	.	A	.	C	.	.	.	.	G	.	.	C	.	.	.	.	.	.	T
OF31	.	.	.	.	G	.	G	.	G	T	C	.	.	.	A	.	.	.	.	.	.	G	.	.	C	.	C	.	.	.	.	T
OF42	.	.	.	T	G	.	G	.	G	.	.	.	.	T	A	.	.	.	.	.	C	.	.	.	C	.	.	.	.	.	.	T
OF47	.	.	.	.	G	T	G	.	G	.	.	G	.	T	A	.	.	.	.	T	C	.	.	.	C	.	C	.	.	.	.	-
OF49	.	.	.	.	.	.	.	.	.	.	.	.	.	.	.	.	.	.	.	.	C	.	.	.	C	.	C	.	.	.	T	T
OF60	.	.	.	.	G	.	G	.	G	.	.	.	.	.	A	T	A	.	.	.	.	.	.	.	C	.	C	.	.	.	.	-
OF74	.	.	.	.	G	T	G	.	G	.	.	.	.	.	.	.	.	.	.	T	.	.	A	.	C	.	.	.	.	.	.	T
Dmad	.	.	C	.	G	.	G	.	G	.	.	.	.	.	.	.	.	.	.	.	.	.	.	.	C	.	C	.	.	.	.	T
Dgua	.	.	C	.	G	.	G	.	G	.	.	.	.	.	.	.	.	.	.	.	.	.	A	T	C	.	C	.	.	.	.	T

G) *Su(z)12*

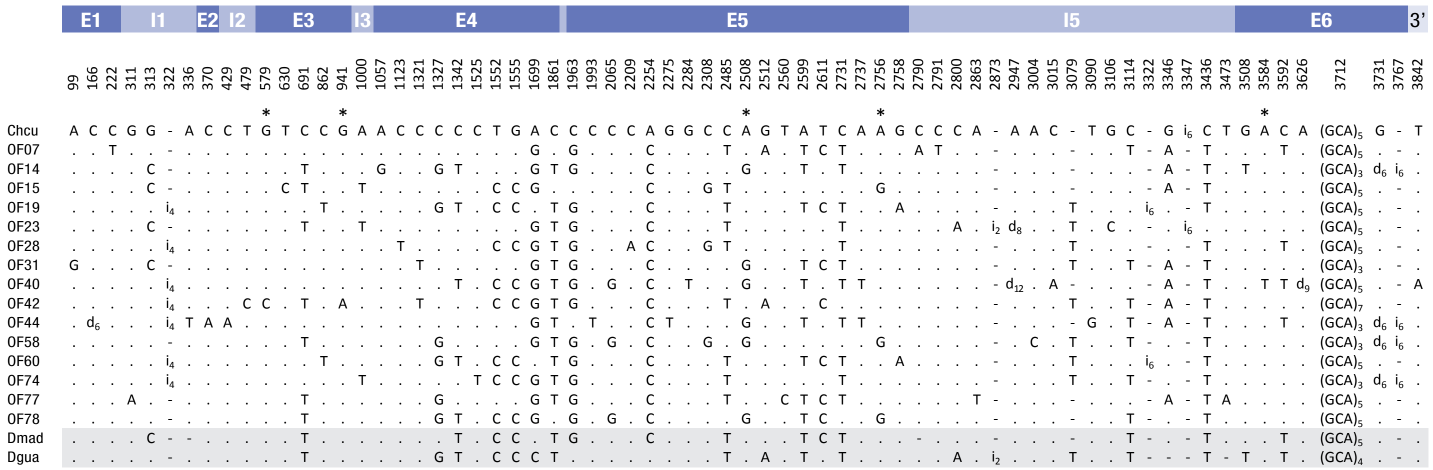

E1
I1
E2
I2
E3
I3
E4
E5
I5
E6
3'
Chcu
OF07
OF14
OF15
OF19
OF23
OF28
OF31
OF40
OF42
OF44
OF58
OF60
OF74
OF77
OF78
Dmad
Dgua

H) *Pcl*

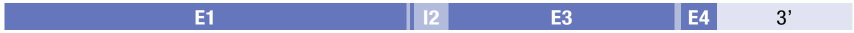

	100	115	259	428	433	455	461	554	561	592	604	605	706	820	928	968	995	1123	1289	1315	1501	1811	1820	1909	2131	2415	2417	2668	2722	2728	2745	2770	2771	2791	2798	3203	3206	3391	3402	3415	3617	3622	3687
				*			*	*	*			*							*	*						*					*		*			*	*						
Chcu	A	A	T	G	T	T	T	G	G	C	C	G	C	G	C	-	C	T	A	T	A	T	C	A	C	A	C	A	G	A	T	C	G	T	T	G	C	T	T	-	A	T	G
OF01	G	.	.	.	C	.	C	.	A	.	A	.	.	.	G	-	.	C	.	.	.	.	.	.	G	.	T	.	.	.	.	.	.	.	.	.	.	.	.	-	C	.	C
OF07	G	.	.	.	.	.	.	.	.	.	.	.	.	.	.	-	.	.	.	.	.	.	.	.	G	.	.	.	.	G	.	.	.	A	C	.	.	.	.	-	.	.	C
OF14	G	.	.	.	C	d_3	C	.	.	.	.	.	.	.	G	-	.	C	.	.	.	.	.	.	G	.	T	.	.	.	.	.	.	.	.	.	.	.	C	-	.	.	C
OF15	G	.	.	.	.	.	.	T	.	.	.	.	.	.	.	-	.	.	.	.	.	.	.	.	.	.	.	.	.	G	.	.	.	A	C	.	.	.	.	-	.	.	C
OF18	G	.	.	.	C	.	C	.	.	.	.	.	.	A	G	-	.	C	.	.	.	C	.	T	.	.	.	.	.	G	A	.	.	.	.	.	.	.	.	-	.	.	C
OF21	G	.	.	.	C	.	C	.	.	.	.	.	.	.	G	-	.	C	.	.	.	C	.	.	G	.	.	.	.	G	A	.	.	.	.	.	.	.	.	-	.	.	C
OF23	G	.	.	.	.	.	.	.	.	.	.	.	A	.	.	-	.	.	.	.	.	.	.	.	.	.	.	.	.	.	.	.	.	.	.	.	.	.	.	-	.	.	C
OF28	G	C	.	.	C	.	C	.	.	.	.	.	.	.	.	-	.	.	.	.	.	.	A	.	.	.	.	.	A	.	.	T	T	.	.	.	.	.	.	-	.	.	C
OF31	G	.	.	.	C	.	.	.	.	.	.	T	.	.	G	-	.	C	.	.	.	.	.	.	.	.	.	.	.	G	A	.	.	.	.	.	.	.	.	-	.	.	C
OF40	G	.	.	.	.	.	.	.	.	.	.	.	.	.	.	-	.	.	.	.	.	.	.	.	.	.	.	.	.	.	.	T	T	.	.	.	.	.	.	-	.	.	C
Of42	G	.	.	.	C	.	C	.	.	.	.	.	.	.	G	-	.	C	.	.	.	C	.	T	.	.	T	.	.	.	.	.	.	.	.	.	.	.	C	i_{180}	.	.	C
OF49	G	.	.	.	C	.	C	.	.	.	.	.	.	.	G	-	d_6	C	.	G	T	.	.	.	.	G	.	.	.	.	.	.	.	.	.	A	T	.	.	-	.	.	C
OF60	G	.	C	A	C	.	C	.	A	.	.	.	.	.	G	i_6	.	C	T	.	.	C	.	T	G	G	.	C	.	G	A	.	.	.	.	A	T	A	.	-	.	.	C
OF74	G	.	.	.	C	.	C	.	A	.	.	.	.	.	G	-	.	C	.	.	.	.	.	.	G	.	T	.	.	.	.	.	.	.	.	.	.	.	.	-	.	.	C
OF78	G	.	.	.	C	.	.	.	.	G	.	.	.	.	G	-	.	C	.	.	.	.	.	.	G	.	.	.	.	.	.	T	T	.	.	.	.	.	.	-	.	G	C
Dmad	G	.	.	.	C	.	.	.	.	.	.	.	.	.	G	i_6	d_6	C	.	G	.	C	.	.	G	.	.	.	.	G	.	.	.	.	C	.	.	.	.	-	.	.	C
Dgua	G	G	.	.	C	.	.	.	.	.	.	.	.	.	G	-	.	C	.	G	.	C	.	.	A	.	.	C	.	G	.	.	.	.	C	.	.	.	.	-	.	.	C

I) *EscI*

5’ — E1 — I1 — E2 — I2 — E3 — 3’

	5	11	16	17	18	45	50	55	59	66	99	101	130	160	169	172	187	194	210	214	220	233	248
																		*	**			*	*
OF01	T	T	G	A	$(A)_1$	T	A	G	T	T	C	A	G	C	G	G	G	A	T	C	G	G	C
OF07	.	C	C	.	$(A)_4$	.	T	.	.	.	.	.	.	.	C	.	A	G	C	.	A	.	T
OF13	.	C	C	.	$(A)_4$	.	T	.	.	G	.	.	A	.	C	.	A	G	C	.	A	.	T
OF14	.	.	C	T	$(A)_5$	G	.	.	.	.	.	.	.	.	.	.	.	.	.	.	.	.	T
OF15	.	.	C	T	$(A)_4$	.	.	A	.	.	A	T	.	.	C	.	A	G	.	.	.	.	.
OF18	.	.	C	T	$(A)_4$	.	.	A	.	.	A	T	.	.	C	A	A	G	C	.	A	.	T
OF19	.	.	C	T	$(A)_4$	.	.	A	.	.	A	T	.	.	C	.	A	G	C	.	A	.	T
OF21	A	.	C	T	$(A)_4$	.	.	A	.	.	A	T	.	.	C	.	A	G	C	.	A	.	T
OF23	.	.	C	T	$(A)_4$	.	.	A	.	.	A	T	.	.	.	.	.	.	.	.	.	.	T
OF31	.	.	C	T	$(A)_4$	.	.	A	C	.	.	.	.	.	C	.	A	G	C	.	A	.	T
OF42	.	.	.	.	$(A)_1$	.	.	.	.	.	.	.	.	A	.	.	.	.	.	.	.	.	.
OF47	.	.	C	T	$(A)_4$	.	.	A	.	.	A	T	.	.	C	.	A	G	C	.	A	.	T
OF49	.	.	.	.	$(A)_1$	.	.	.	.	.	.	.	.	.	C	.	A	G	C	.	A	.	T
OF60	.	.	C	T	$(A)_4$	.	.	A	.	.	A	T	.	.	C	.	A	G	C	.	A	.	T
OF74	.	.	C	T	$(A)_4$	.	.	A	.	.	A	T	.	.	C	.	A	G	A	A	A	T	T
Dmad	.	.	C	.	$(A)_4$	.	T	.	.	.	A	.	.	.	C	.	A	G	.	.	A	.	T
Dgua	.	.	A	.	$AG(A)_2$	.	.	A	.	.	A	.	.	.	.	.	.	.	.	T	A	.	T

	334	412	418	430	462	464	615	642	713	730	740	823	847	859	892	910	928	940	956	988	1024	1051	1060	1084	1150	1246	1252	1264	1372	1378	1402	1441	1462	1486	1663	1681	1686	1692	1706	1712	1751	1753	1758	1759
OF01	C	A	A	A	T	C	T	G	G	C	G	G	C	C	A	G	C	G	C	C	C	C	C	T	C	T	C	C	G	G	T	C	G	G	T	T	A	T	G	A	C	C	G	C
OF07	T	.	G	G	C	.	.	.	A	T	A	A	.	.	.	.	.	.	.	.	.	T	T	G	.	.	.	.	.	.	.	T	.	.	.	.	T	.	A	.	G	.	T	.
OF13	.	.	.	G	.	T	.	T	.	.	A	A	T	.	.	.	.	.	.	.	.	T	T	G	A	.	.	.	.	.	.	.	.	.	.	.	T	.	A	.	G	.	T	.
OF14	T	.	G	G	C	.	G	.	A	.	A	A	T	.	.	.	.	.	.	.	.	.	.	.	.	.	.	.	.	.	.	T	.	.	.	A	T	.	A	.	G	.	T	G
OF15	.	.	.	G	.	.	.	.	A	T	A	A	.	.	.	.	.	.	T	.	.	T	T	G	.	.	.	.	.	.	.	.	.	.	C	A	C	A	A	.	G	.	T	.
OF18	T	C	G	G	.	.	G	.	.	.	.	A	.	T	G	C	.	A	T	.	.	T	T	G	.	C	T	T	.	.	C	T	.	.	.	A	T	.	A	.	.	.	.	.
OF19	T	C	G	G	.	.	G	.	A	.	A	A	.	.	.	.	.	.	.	.	.	T	T	G	.	.	.	.	.	.	.	T	.	.	.	A	T	.	A	.	G	.	T	.
OF21	.	.	G	G	C	.	G	.	A	T	A	A	.	.	G	C	G	.	.	T	.	T	T	G	.	.	.	.	.	.	.	T	.	.	.	.	T	.	A	.	.	T	.	.
OF23	T	.	.	.	.	.	.	.	.	.	A	A	.	.	G	C	.	A	T	.	.	T	T	G	.	.	.	.	.	A	.	T	.	A	C	A	C	A	A	.	.	.	.	.
OF31	.	.	.	G	.	.	.	.	.	.	A	A	.	.	.	.	.	.	.	.	T	.	.	.	.	.	.	.	.	.	.	T	A	.	.	A	T	.	A	.	.	T	.	.
OF42	.	.	.	G	.	.	.	.	.	.	A	A	T	.	.	.	.	.	.	.	.	.	.	.	.	.	.	.	.	.	.	.	.	.	C	A	C	.	A	.	.	.	.	.
OF47	T	.	G	G	.	.	.	T	.	.	.	A	T	.	.	.	.	.	.	.	.	.	.	G	.	.	.	.	.	.	.	T	.	.	.	A	T	.	A	T	G	.	T	.
OF49	T	.	.	G	.	.	.	T	.	.	A	A	.	.	G	C	.	A	T	.	.	T	T	G	.	.	.	.	.	.	.	.	.	.	.	.	T	.	A	.	.	.	.	.
OF60	T	C	G	G	.	.	G	.	A	.	A	A	.	.	.	.	.	.	.	.	.	T	T	G	.	.	.	.	.	.	.	T	.	.	.	A	T	.	A	.	G	.	T	.
OF74	T	.	G	G	C	.	G	.	A	T	A	A	.	.	G	C	.	.	.	T	.	T	T	G	.	.	.	.	A	.	.	.	.	.	.	A	T	.	.	.	.	.	.	.
Dmad	T	.	.	G	C	.	G	.	.	.	A	A	.	.	G	.	.	.	.	T	.	.	T	G	.	.	.	T	.	.	.	T	.	.	C	A	C	.	A	.	.	.	.	.
Dgua	T	.	.	.	.	.	.	.	.	.	A	A	.	.	G	.	.	.	.	.	.	T	.	.	.	C	.	T	.	.	.	T	.	.	.	A	T	.	A	.	.	.	.	.

J) *Psc*

	18	60	91	121	145	158	188	234	274	310	373	466	505	516	569	570	595	603	612	621	640	641	646	657	660	662	671	682	685	694	703	706	707	708	739	759	765	805	833	839	845	872	874	892	907	914	958	960	981	1068	1141	1147	1198	1219	1225	1292	1310	1315	1327	1391	1398	1399	1400	1411	1414	1530	1532	1541	1553	1559	1569	1621	1702	1765	1787	1789	1804	1820	1946	1948	1975	1989	2011	2015	2043	2067	2140	2227	2286	2308
	5’																																																			E1																									I1												
																																																						*				*			*		*		*	*		*			*	*															
Chcu	T	C	G	(ACTGGC)$_2$	A	G	C	G	C	A	A	d$_5$	C	C	G	C	C	C	C	G	A	-	A	A	C	T	T	G	C	A	T	A	T	A	T	C	G	G	T	A	G	A	A	A	d$_7$	i$_{26}$	G	C	C	G	T	T	C	G	A	A	A	A	T	i$_6$	C	A	i$_9$	G	A	C	-	G	A	-	A	T	G	T	C	G	G	C	A	C	T	T	C	-	T	C	A	G	G	G
OF01	G	.	.	(ACTGGC)$_2$	.	.	.	.	A	.	.	C	.	.	.	.	.	A	.	.	.	-	G	.	.	G	.	.	T	.	.	.	.	.	.	.	.	.	C	T	T	.	.	.	G	-	.	T	.	.	.	.	.	.	.	.	G	G	.	i$_6$	A	G	i$_9$	.	.	A	i$_3$	.	.	-	C	.	A	.	.	.	.	T	C	.	.	A	T	-	.	T	G	.	A	A
OF07	.	T	.	-	.	.	.	.	.	.	.	C	.	.	A	A	.	A	.	.	.	-	G	.	.	G	.	.	T	.	.	.	.	.	.	.	.	.	.	.	.	.	.	.	G	-	.	T	.	.	.	A	.	C	C	.	G	.	.	i$_6$	A	G	i$_9$	.	.	.	-	.	.	-	.	.	A	.	.	.	.	.	C	.	A	A	.	-	.	.	.	T	A	A
OF14	.	.	.	(ACTGGC)$_1$	.	.	.	.	.	.	.	C	.	.	A	A	.	.	.	.	.	-	G	.	.	G	.	.	.	.	.	.	.	.	.	.	.	.	.	.	.	C	.	.	G	-	.	T	.	.	.	.	A	.	.	.	.	.	.	i$_6$	A	G	i$_9$	.	.	.	i$_3$	.	.	-	C	.	A	.	.	.	.	T	C	.	.	A	.	-	.	.	G	.	.	.
OF15	.	.	.	(ACTGGC)$_2$	.	.	.	.	A	.	.	C	.	.	.	.	.	A	.	.	.	-	G	.	.	G	.	.	.	.	.	.	.	.	C	T	.	.	.	.	T	C	.	.	G	-	.	T	.	.	.	.	A	.	.	.	G	.	.	-	A	G	i$_9$	.	.	.	-	.	.	i$_6$	C	C	A	.	.	.	.	T	C	.	.	A	.	-	.	.	.	.	A	.
OF18	.	.	.	(ACTGGC)$_2$	.	.	.	.	A	.	.	C	T	.	A	A	.	.	.	.	.	i$_4$	.	.	.	G	.	.	T	.	.	.	.	.	.	T	.	.	.	.	.	C	.	.	G	-	.	T	.	.	.	.	A	.	C	.	.	.	.	i$_6$	A	G	i$_9$	.	.	.	-	C	.	-	.	.	A	.	G	.	.	.	C	G	.	A	.	-	.	.	G	T	A	A
OF21	.	.	.	(ACTGGC)$_2$	.	C	.	.	.	.	.	C	.	T	A	A	.	.	.	.	.	-	.	T	T	G	d$_2$	.	T	d$_2$	C	.	A	G	.	.	.	C	.	.	T	.	G	G	G	-	.	T	.	.	.	.	.	.	.	.	.	.	.	i$_6$	A	G	i$_9$	.	.	.	-	.	.	-	C	.	A	.	.	.	.	T	C	.	.	A	.	-	.	.	G	T	A	A
OF23	.	.	.	(ACTGGC)$_2$	.	.	.	.	.	.	.	C	.	.	.	.	.	.	T	.	.	-	.	.	.	.	.	.	.	.	.	.	.	.	.	.	.	.	.	.	.	C	.	.	G	-	.	T	.	.	.	.	A	.	C	.	.	.	.	i$_6$	A	G	i$_9$	C	.	.	-	C	.	-	.	.	A	.	G	.	.	.	C	.	.	A	.	-	.	.	.	.	A	.
OF28	.	.	.	(ACTGGC)$_2$	.	.	.	.	.	.	.	C	.	.	.	.	.	A	.	.	.	-	G	.	.	G	.	.	T	.	.	.	.	.	.	.	A	.	.	.	.	.	.	.	G	-	.	T	.	.	.	.	.	.	.	.	.	.	.	i$_6$	A	G	i$_9$	.	G	.	-	.	.	-	.	.	.	.	.	.	.	.	C	.	.	A	.	-	.	.	G	.	.	.
OF31	.	.	.	(ACTGGC)$_2$	.	.	.	.	A	.	.	C	.	.	.	.	.	A	.	.	.	-	G	.	.	G	.	A	.	.	.	.	.	.	.	T	.	.	.	.	.	C	.	.	G	-	.	T	.	A	.	.	A	C	C	.	G	.	.	i$_6$	A	G	i$_9$	.	.	.	-	.	.	-	.	.	A	G	.	.	.	.	C	.	.	A	.	-	.	.	.	.	.	.
OF40	.	.	.	(ACTGGC)$_2$	.	.	.	.	.	G	.	C	.	.	.	.	.	A	.	.	T	-	G	.	.	G	.	.	T	.	.	.	.	.	.	T	.	.	.	.	.	C	.	.	G	-	.	T	.	.	.	.	.	.	.	.	.	.	.	i$_6$	A	G	i$_9$	.	.	.	-	.	G	i$_6$	C	.	.	.	.	.	.	T	C	.	.	A	.	-	.	.	G	.	.	.
OF47	.	.	A	(ACTGGC)$_2$	.	.	.	.	.	.	.	C	.	.	.	.	.	.	.	.	.	-	.	.	.	.	.	.	.	.	.	.	.	.	.	.	.	.	.	.	.	C	.	.	G	-	C	.	.	.	G	.	A	.	.	.	.	.	.	i$_6$	A	G	i$_9$	.	.	.	-	.	.	-	.	.	A	.	.	A	.	.	C	.	.	A	.	-	G	.	.	.	A	.
OF49	.	.	.	(ACTGGC)$_2$	G	.	.	A	.	.	.	C	.	.	A	A	.	.	.	.	.	-	.	.	.	.	.	.	T	.	.	C	.	.	.	.	.	.	.	.	.	C	.	.	G	-	.	T	.	.	.	.	.	.	.	.	G	.	C	i$_6$	A	G	i$_9$	.	.	.	-	.	.	i$_6$	C	.	A	.	G	.	.	.	C	.	.	A	.	-	.	.	G	.	A	A
OF74	.	.	.	(ACTGGC)$_2$	.	.	G	.	.	.	.	C	.	.	.	.	.	A	.	.	.	-	G	.	.	.	.	.	T	.	.	.	.	.	.	.	.	.	.	.	.	.	.	.	G	-	.	T	.	A	.	.	.	.	.	.	.	.	.	-	A	G	-	.	.	.	-	.	.	i$_6$	C	.	A	.	.	.	.	T	C	.	.	A	.	-	.	.	G	T	A	A
OF78	.	.	.	(ACTGGC)$_4$	.	.	.	.	.	.	d$_{14}$	C	.	.	A	A	T	.	.	T	.	i$_4$	.	.	.	G	.	.	T	.	.	.	.	.	.	T	.	.	.	.	.	.	.	.	G	-	.	T	A	.	.	.	.	.	.	d$_3$	.	.	.	i$_6$	A	G	i$_9$	.	.	.	-	.	.	-	.	.	A	.	.	.	A	T	C	.	.	A	.	i$_{12}$	.	.	.	.	A	.
Dmad	.	.	.	(ACTGGC)$_1$	.	.	.	.	.	.	.	C	.	.	.	.	.	.	.	.	.	-	.	T	T	G	.	-	-	-	.	.	.	.	.	T	.	.	C	T	.	C	.	.	G	-	.	T	.	.	.	.	A	C	C	.	G	.	.	-	A	G	i$_6$	.	.	.	-	.	.	-	C	.	A	.	.	.	.	.	C	.	.	A	.	-	.	.	.	.	A	.
Dgua	.	.	.	(ACTGGC)$_3$	.	.	.	-	.	.	.	C	-	.	.	.	.	.	.	.	.	-	.	.	.	G	.	.	T	.	.	.	.	.	G	T	.	T	.	T	C	C	.	-	G	-	.	T	.	.	.	.	A	.	.	.	G	.	.	-	A	G	-	.	.	.	-	.	.	-	C	.	A	.	.	.	.	T	C	.	.	A	.	-	.	.	.	.	.	.

	2309	2321	2336	2358	2383	2386	2416	2437	2440	2442	2448	2462	2466	2474	2487	2510	2548	2571	2586	2610	2624	2625	2637	2642	2645	2706	2780	2785	2788	2837	2910	2914	2923	2932	3016	3073	3189	3201	3221	3362	3363	3364	3507	3511	3520	3537	3577	3594	3601	3602	3615	3690	3698	3710	3762	3768	3816	3987	4159	4163	4208	4341	4366	4427	4433	4441	4514	4560	4561	4602	4717	4776	4791	4792	4845	4882	4930	4943	4958	4963	4965	4977	5014	5061
	I1																																																									E2	I2																										
Chcu	G	A	T	C	T	T	A	T	A	T	A	C	A	T	G	C	C	G	T	A	C	i$_3$	T	C	TCCC(TC)$_{13}$	G	C	T	C	G	A	T	G	A	C	T	C	C	-	-	-	A	G	-	T	C	T	T	C	C	G	A	G	T	T	G	C	T	A	A	(ATGGGA)$_3$	G	-	G	T	T	C	G	T	C	C	T	G	G	C	A	A	A	T	G	C	T	T	T
OF01	A	.	.	A	.	.	.	.	.	.	.	.	.	.	A	.	T	A	.	.	-	-	.	.	(TC)$_{12}$	A	.	C	.	.	.	.	.	.	.	.	.	.	-	T	-	-	.	-	.	.	.	.	.	.	d$_{16}$	.	.	.	.	-	.	.	.	.	(ATGGGA)$_2$	T	i$_4$	.	.	.	.	T	.	.	.	.	.	.	.	G	.	.	.	.	.	.	.	.
OF07	A	.	.	.	.	.	.	.	.	.	.	.	.	C	.	.	.	A	.	.	.	i$_9$	.	.	(TC)$_6$TT(TC)$_6$TT(TC)$_8$	.	.	C	T	.	.	.	.	.	.	.	.	.	-	T	G	.	.	-	.	.	.	.	.	.	.	.	.	.	.	.	.	.	.	.	(ATGGGA)$_3$	T	-	.	.	.	T	.	.	.	.	.	.	.	.	.	.	C	.	.	.	.	.	.
OF14	.	.	.	A	A	.	.	A	.	.	.	G	G	.	.	A	T	A	A	G	.	i$_3$	C	G	(TC)$_{11}$	.	.	C	.	.	.	.	.	.	.	.	.	.	-	-	-	.	T	-	.	.	.	.	.	T	.	.	.	.	.	-	.	.	.	.	(ATGGGA)$_3$	T	-	.	.	.	.	T	.	.	.	.	.	.	.	.	.	.	.	.	.	.	.	G
OF15	.	.	.	A	.	.	.	.	.	C	.	.	.	.	A	A	T	A	.	.	.	i$_9$	.	.	(TC)$_6$TT(TC)$_8$	.	.	C	.	.	.	.	C	T	.	.	.	.	-	T	G	.	.	-	.	T	.	A	.	.	.	.	.	.	.	-	.	.	.	.	(ATGGGA)$_2$	T	-	.	.	.	.	.	.	.	.	.	.	.	.	.	.	.	.	.	.	.	.	G
OF18	A	.	.	A	.	.	G	.	T	.	T	.	.	.	.	.	.	A	.	.	.	i$_3$	.	.	(TC)$_{18}$	.	.	C	.	.	.	.	.	.	.	.	.	G	T	T	G	.	.	-	.	.	.	.	.	.	.	.	.	.	.	-	T	.	.	.	(ATGGGA)$_2$	T	-	T	.	A	.	T	.	.	.	.	.	.	T	G	.	.	.	.	.	.	.	G
OF21	A	.	.	A	.	.	G	.	T	.	T	.	.	.	.	.	.	A	.	.	.	i$_9$	.	.	(TC)$_6$TT(TC)$_8$	.	.	C	.	T	.	.	.	.	.	.	.	.	-	T	G	.	.	-	.	.	.	.	.	.	.	G	.	.	A	-	.	.	.	.	(ATGGGA)$_4$	T	-	.	.	.	.	.	.	.	.	.	.	.	.	.	.	.	.	.	.	.	.	G
OF23	.	T	.	A	.	.	.	.	.	.	.	.	.	.	.	.	.	.	.	.	.	i$_9$	.	.	(TC)$_{21}$	.	.	C	.	.	G	C	.	.	A	.	.	.	-	T	G	.	.	-	.	.	.	.	.	.	.	.	.	.	.	.	.	.	.	.	(ATGGGA)$_3$	T	i$_4$	.	G	.	.	.	.	.	.	.	.	.	.	.	.	.	.	.	.	.	.	G
OF28	.	.	.	.	.	.	.	.	.	.	.	.	.	.	.	.	.	.	.	.	.	i$_9$	.	.	(TC)$_7$	.	.	C	.	.	.	.	.	.	.	A	.	.	T	T	G	.	.	T	C	.	.	.	.	.	d$_{16}$	.	.	.	.	-	.	.	G	.	(ATGGGA)$_3$	T	-	.	.	.	.	T	.	.	.	.	A	T	.	.	C	.	G	.	.	.	.	G
OF31	.	.	.	A	.	.	.	.	.	.	.	.	.	.	.	.	.	.	.	.	.	i$_9$	.	.	(TC)$_{30}$	.	.	C	.	.	.	.	.	.	.	A	.	.	-	T	G	.	.	-	.	.	.	.	.	.	.	.	.	.	.	.	.	.	.	.	(ATGGGA)$_3$	T	i$_4$	.	.	.	.	T	.	.	T	.	.	.	.	.	.	.	.	T	.	.	.	.
OF40	.	.	.	A	.	G	.	.	.	.	.	.	.	.	A	A	T	A	.	.	.	i$_3$	.	.	(TC)$_{14}$	.	.	C	.	.	.	.	.	.	.	.	.	.	-	-	-	.	.	-	.	.	.	.	A	T	.	.	.	C	.	-	.	.	.	.	(ATGGGA)$_2$	T	-	T	.	A	.	T	.	.	.	.	.	.	.	G	.	.	.	.	.	.	.	G
OF47	.	.	.	A	.	.	.	.	.	.	T	.	.	.	T	A	T	A	.	.	.	i$_3$	.	.	(TC)$_5$	.	T	C	.	.	.	.	.	.	.	.	.	.	-	T	G	.	.	-	.	.	G	.	.	.	.	.	.	.	.	-	.	C	.	.	(ATGGGA)$_2$	T	-	.	.	.	.	.	.	.	.	.	.	.	.	.	.	.	.	.	.	.	.	G
OF49	A	.	.	A	.	.	.	.	.	.	T	.	.	C	.	A	.	A	.	.	.	i$_9$	.	.	(TC)$_5$	.	.	C	.	.	.	.	.	.	.	.	.	.	-	T	G	.	.	-	.	.	.	.	.	.	.	.	T	.	.	.	.	.	G	T	(ATGGGA)$_3$	T	-	.	.	.	T	T	.	A	.	A	.	.	.	.	.	C	.	.	.	.	d$_5$	.
OF74	A	.	.	A	.	.	G	.	.	.	.	.	.	C	.	A	.	A	.	.	.	i$_9$	.	.	(TC)$_6$TT(TC)$_8$	.	.	C	T	.	.	.	.	.	.	.	.	.	-	T	G	.	.	-	.	.	.	.	.	.	.	.	.	.	.	-	T	.	.	.	(ATGGGA)$_2$	T	-	T	.	A	.	T	.	.	.	.	.	.	.	G	.	.	.	.	.	.	.	G
OF78	.	.	A	A	.	.	.	.	.	C	.	.	.	.	.	.	.	A	.	.	T	i$_9$	.	.	(TC)$_{11}$	.	.	C	.	.	.	.	.	.	.	.	T	.	-	T	G	.	.	-	.	.	.	.	.	.	d$_{16}$	.	.	.	.	.	.	.	.	.	(ATGGGA)$_3$	T	-	.	G	.	T	T	d$_2$	T	.	.	.	.	.	G	.	.	.	.	T	C	.	.
Dmad	.	G	.	A	.	.	.	A	.	.	.	G	.	.	A	A	T	.	.	.	.	i$_3$	.	.	(TC)$_{17}$	.	T	C	.	.	.	.	.	.	.	A	.	.	T	T	G	.	.	-	.	.	.	.	.	.	.	.	.	.	.	.	.	.	.	.	(ATGGGA)$_3$	T	-	.	G	.	.	T	.	.	.	.	.	.	.	G	.	.	.	.	.	.	.	G
Dgua	.	G	.	-	C	.	.	A	.	.	.	G	.	.	A	A	T	A	.	.	.	-	-	-	-	.	T	C	.	.	-	-	.	.	.	A	.	.	T	T	-	-	.	-	.	.	.	.	.	.	.	.	.	.	.	-	.	C	.	.	(ATGGGA)$_4$	T	-	.	G	.	.	.	.	.	.	.	.	.	.	.	.	.	G	.	.	.	.	G

continued on next page

J) *Psc*

continued from previous page

Region	I2																														
Position	5090	5140	5176	5365	5366	5416	5418	5432	5439	5479	5685	5690	5694	5720	5750	5814	5822	5831	5846	5850	5859	5868	5870	5872	5877	5889	5908	5912	5913	5914	5923	5926
Chcu	G	C	A	T	(G)$_{6}$	G	G	T	C	G	G	A	-	G	C	A	A	C	C	A	C	A	G	A	C	C	G	A	G	G	A	d$_{57}$
OF01	.	.	.	.	(G)$_{7}$	.	A	.	.	.	.	.	A	.	G	.	C	.	A	.	.	.	T	G	.	.	.	C	A	.	T	d$_{33}$
OF07	.	A	.	.	(G)$_{6}$	.	.	.	.	.	.	.	A	C	.	.	.	.	.	.	.	.	T	G	.	.	.	C	A	.	T	d$_{33}$
OF14	.	.	.	.	(G)$_{6}$	.	.	.	.	.	.	.	A	.	.	.	.	.	A	.	A	.	.	.	A	.	.	C	A	.	T	d$_{33}$
OF15	.	A	G	.	(G)$_{6}$	.	.	.	.	.	.	.	A	C	.	.	.	.	.	.	.	.	.	.	.	.	.	C	A	.	T	d$_{33}$
OF18	.	A	.	.	(G)$_{6}$	.	.	.	.	.	.	.	-	.	.	.	.	.	.	.	.	.	.	.	.	.	.	.	.	.	.	d$_{57}$
OF21	.	A	.	.	(G)$_{6}$	A	.	.	.	.	.	.	A	.	.	G	.	.	A	.	.	.	T	G	.	.	.	C	A	T	T	d$_{33}$
OF23	.	A	.	.	(G)$_{6}$	.	.	.	.	.	.	.	A	.	.	.	.	.	A	G	.	.	T	G	.	.	.	C	A	.	T	d$_{33}$
OF28	A	A	.	G	(G)$_{7}$	.	.	.	.	.	d$_{3}$	C	-	.	.	.	.	.	A	G	.	.	T	G	.	A	.	C	A	.	T	d$_{33}$
OF31	.	A	.	.	(G)$_{8}$	.	.	.	.	.	.	.	A	.	.	.	.	.	A	.	.	G	T	G	.	.	.	C	A	.	T	d$_{33}$
OF40	.	A	.	.	(G)$_{7}$	.	A	.	.	A	.	.	-	.	.	.	.	.	.	.	.	.	.	.	.	.	.	.	.	.	.	d$_{57}$
OF47	.	A	.	.	(G)$_{6}$	A	.	.	.	.	.	.	A	C	.	.	.	A	A	.	.	.	T	G	.	.	.	C	A	.	T	d$_{33}$
OF49	.	A	.	.	(G)$_{6}$	.	.	C	A	.	.	.	A	.	.	.	.	.	.	.	.	.	.	.	.	.	T	C	A	.	T	d$_{33}$
OF74	.	A	.	.	(G)$_{7}$	.	A	.	.	.	.	.	-	.	.	.	.	.	.	.	.	.	.	.	.	.	.	.	.	.	.	d$_{57}$
OF78	.	A	.	.	(G)$_{6}$	.	.	.	.	.	.	.	A	.	.	.	.	.	.	.	.	.	T	T	.	.	.	C	A	.	T	d$_{33}$
Dmad	.	A	.	.	(G)$_{6}$	.	.	.	.	.	.	.	A	.	.	.	.	T	.	.	.	.	T	G	.	.	.	.	.	.	.	d$_{33}$
Dgua	.	A	.	.	(G)$_{5}$	.	.	C	.	.	.	.	A	.	.	.	.	T	.	.	.	.	C	G	-	-	.	C	.	.	.	G

Region	I2																									
Position	5983	5988	5989	6009	6016	6021	6034	6045	6121	6137	6146	6153	6156	6198	6270	6278	6320	6468	6531	6538	6539	6540	6563	6579	6610	6611
Chcu	(T)$_{2}$	A	T	A	C	C	A	T	A	C	T	A	G	C	G	-	A	T	C	T	A	A	(AG)$_{5}$	A	T	(T)$_{7}$
OF01	(T)$_{3}$	T	.	C	.	.	.	.	.	.	.	T	.	.	.	-	.	.	T	C	.	.	(AG)$_{6}$	G	.	(T)$_{7}$
OF07	(T)$_{2}$	.	.	.	.	.	.	.	G	A	C	.	.	.	.	i$_{6}$	G	.	T	C	C	C	(AG)$_{5}$	.	.	(T)$_{8}$
OF14	(T)$_{2}$	.	C	.	.	.	.	.	.	.	.	T	.	.	.	-	.	.	T	C	.	.	(AG)$_{8}$	.	.	(T)$_{6}$
OF15	(T)$_{5}$	T	.	C	.	.	.	.	.	.	.	.	.	.	.	-	.	.	T	C	.	.	(AG)$_{5}$	.	.	(T)$_{7}$
OF18	(T)$_{2}$	.	.	.	.	.	.	.	.	.	.	.	.	.	C	i$_{6}$	.	.	T	C	.	.	(AG)$_{6}$	.	.	(T)$_{7}$
OF21	(T)$_{2}$	.	.	.	.	.	.	.	.	.	.	.	.	G	.	-	.	.	T	C	.	.	(AG)$_{6}$	.	A	(T)$_{9}$
OF23	(T)$_{2}$	.	C	.	.	.	.	.	.	.	.	.	.	.	.	-	.	.	T	C	.	.	(AG)$_{6}$	.	.	(T)$_{8}$
OF28	(T)$_{2}$	.	C	.	T	.	.	d$_{26}$	.	.	.	T	.	.	.	-	.	.	T	C	.	.	(AG)$_{7}$	.	.	(T)$_{6}$
OF31	(T)$_{2}$	.	.	.	.	A	.	.	.	.	.	T	.	.	.	-	.	G	T	C	.	.	(AG)$_{6}$	.	.	(T)$_{8}$
OF40	(T)$_{2}$	.	.	.	.	.	.	.	.	.	.	.	.	.	C	i$_{6}$	.	.	T	C	.	.	(AG)$_{6}$	.	.	(T)$_{7}$
OF47	(T)$_{2}$	.	.	.	.	.	.	.	.	.	.	T	.	.	.	-	.	.	.	.	.	.	(AG)$_{5}$	.	.	(T)$_{6}$
OF49	(T)$_{4}$	T	.	.	.	.	T	.	.	.	.	.	A	.	.	-	.	.	T	C	.	.	(AG)$_{5}$	.	.	(T)$_{7}$
OF74	(T)$_{2}$	.	.	.	.	.	.	.	.	.	.	.	.	.	C	i$_{6}$	.	.	T	C	.	.	(AG)$_{6}$	.	.	(T)$_{7}$
OF78	(T)$_{2}$	.	.	.	.	.	.	.	.	.	.	.	.	.	.	-	.	.	T	C	.	.	(AG)$_{6}$	.	.	(T)$_{7}$
Dmad	(T)$_{4}$	T	.	.	.	.	.	.	.	.	.	.	.	.	.	-	.	.	T	C	.	-	(AG)$_{1}$	.	.	(T)$_{11}$
Dgua	(T)$_{2}$	.	.	.	.	.	.	.	.	.	.	.	.	.	.	-	.	.	T	C	.	.	(AG)$_{7}$	.	.	(T)$_{11}$

Region	I2																												
Position	6643	6653	6676	6691	6703	6712	6761	6762	6832	6885	6928	6932	6986	6988	6990	7086	7089	7179	7205	7206	7217	7218	7235	7249	7295	7397	7499	7543	7581
Chcu	T	A	A	T	A	A	T	A	T	G	T	-	G	G	T	T	A	T	T	T	T	G	G	T	G	T	T	C	G
OF01	.	.	.	.	.	G	G	C	A	.	.	-	.	T	d$_{22}$	.	.	.	.	.	-	C	.	G	.	.	.	T	T
OF07	.	.	.	.	.	G	.	C	A	A	.	-	.	T	.	.	.	.	.	C	.	.	.	.	.	.	.	.	.
OF14	.	.	.	.	.	G	G	C	A	.	.	-	.	T	d$_{22}$	.	.	.	.	.	-	C	.	G	.	.	.	.	.
OF15	.	.	-	.	C	.	.	C	A	.	C	-	.	T	d$_{22}$	.	.	.	.	.	-	C	.	.	.	.	.	.	.
OF18	.	T	.	.	.	.	.	C	A	.	.	-	T	T	.	.	.	.	.	.	.	C	.	.	.	.	G	.	.
OF21	.	.	.	.	.	G	.	C	A	.	.	-	.	T	.	.	G	.	.	.	.	C	A	.	.	.	.	.	.
OF23	A	.	.	d$_{4}$	.	.	.	C	A	.	.	-	.	T	.	C	.	C	.	.	.	.	.	.	.	.	.	.	.
OF28	.	.	.	.	C	.	.	C	A	.	.	T	.	T	.	.	.	.	.	.	-	C	.	.	.	C	.	.	.
OF31	.	.	.	.	C	.	.	C	A	.	.	-	.	T	d$_{22}$	.	.	.	.	.	-	C	.	.	C	.	.	.	.
OF40	.	T	.	.	.	.	.	C	A	.	.	-	T	T	.	.	.	.	.	.	.	C	.	.	.	.	G	.	.
OF47	.	.	.	.	.	G	G	C	A	.	.	-	.	T	.	.	.	.	.	.	-	C	.	.	.	.	.	.	.
OF49	.	.	.	.	.	.	.	C	A	.	.	-	T	T	.	.	.	.	.	.	-	C	.	.	.	.	G	.	.
OF74	.	T	.	.	.	.	.	C	A	.	C	-	.	T	d$_{22}$	.	.	.	.	.	.	C	.	.	.	.	G	.	.
OF78	.	.	.	.	.	.	.	C	A	.	.	-	.	T	d$_{22}$	.	.	.	-	.	.	.	.	.	.	.	.	.	.
Dmad	.	.	.	.	.	G	.	C	A	.	.	-	.	T	.	.	.	.	.	.	.	C	.	G	.	.	.	.	.
Dgua	.	.	.	.	.	G	.	C	A	.	.	-	.	T	.	C	.	.	.	.	.	C	.	G	.	.	.	.	.

Region	I2									E3		I3									
Position	7600	7705	7722	7744	7806	7815	7859	7879	7928	7941	7977	8094	8174	8179	8191	8209	8239	8246	8247	8250	8252
Chcu	(TC)$_{7}$TT	C	C	T	d$_{6}$	T	A	i$_{15}$	C	A	C	T	A	T	T	C	T	C	C	C	T
OF01	(TC)$_{48}$TT(TC)$_{2}$	.	.	C	d$_{6}$	C	.	-	.	.	.	.	.	.	C	.	.	.	.	.	.
OF07	(TC)$_{7}$TT	.	.	.	d$_{6}$	.	.	i$_{15}$	.	.	.	.	.	C	C	.	C	-	.	.	.
OF14	(TC)$_{8}$TT	.	.	.	d$_{6}$	.	.	i$_{5}$	.	.	.	A	.	.	C	.	.	-	-	.	.
OF15	(TC)$_{11}$	.	.	C	C	.	.	i$_{5}$	.	.	.	.	.	.	.	.	.	.	.	.	.
OF18	(TC)$_{15}$	.	.	C	d$_{6}$	.	d$_{10}$	i$_{5}$	.	.	.	A	G	.	C	.	.	.	.	.	.
OF21	(TC)$_{10}$	T	.	.	d$_{6}$	.	.	-	.	.	.	.	G	.	C	T	.	-	.	.	.
OF23	(TC)$_{5}$	.	.	C	d$_{6}$	.	.	-	.	G	T	.	.	.	C	.	.	.	.	.	.
OF28	(TC)$_{8}$	.	.	C	C	.	.	i$_{5}$	.	.	.	A	.	C	C	.	.	-	.	.	.
OF31	(TC)$_{13}$	.	T	C	d$_{6}$	.	.	-	.	G	.	.	.	.	C	.	.	.	.	T	.
OF40	(TC)$_{22}$	.	.	C	d$_{6}$	.	d$_{10}$	i$_{5}$	.	.	.	A	G	.	C	.	.	.	.	.	.
OF47	(TC)$_{5}$	.	.	C	d$_{6}$	.	.	-	T	.	.	.	.	.	C	.	.	.	.	.	.
OF49	(TC)$_{16}$	.	.	C	d$_{6}$	.	d$_{10}$	i$_{5}$	.	.	.	A	G	.	C	.	.	.	.	.	.
OF74	(TC)$_{22}$	.	.	C	d$_{6}$	.	d$_{10}$	i$_{5}$	.	.	.	A	G	.	C	.	.	.	.	.	.
OF78	(TC)$_{12}$	.	.	C	C	.	.	i$_{5}$	.	.	.	.	.	.	C	.	.	.	.	.	C
Dmad	(TC)$_{8}$	.	.	.	d$_{6}$	.	.	-	.	.	.	.	.	.	C	.	.	-	.	.	.
Dgua	TCGC(TC)$_{9}$	.	.	C	C	.	.	-	.	.	T	.	.	.	C	.	.	T	.	.	.

Region	E4																
Position	8260	8293	8323	8419	8446	8548	8585	8587	8713	8725	8740	8749	8791	8804	8834	8865	9026
							*								*	*	
Chcu	G	C	C	C	C	G	G	C	T	G	A	T	G	(TCC)$_{7}$	T	T	(AGC)$_{5}$
OF01	.	.	.	.	.	.	.	.	C	A	G	G	.	(TCC)$_{5}$	G	.	(AGC)$_{5}$
OF07	.	.	.	.	.	.	.	.	.	.	G	.	A	(TCC)$_{10}$	.	.	(AGC)$_{5}$
OF14	.	.	.	.	.	.	C	G	.	.	G	G	.	(TCC)$_{5}$	.	.	(AGC)$_{6}$
OF15	.	.	.	.	.	.	.	.	.	.	G	G	.	(TCC)$_{3}$	.	.	(AGC)$_{8}$
OF18	.	.	.	.	.	T	.	.	.	.	G	.	.	(TCC)$_{3}$	.	.	(AGC)$_{5}$
OF21	A	T	T	.	.	.	.	.	.	.	G	G	.	(TCC)$_{3}$	.	.	(AGC)$_{5}$
OF23	.	.	.	T	.	.	.	.	.	.	G	.	.	(TCC)$_{3}$	.	.	(AGC)$_{6}$
OF28	A	.	.	.	.	.	.	.	C	.	G	G	.	(TCC)$_{3}$	.	C	(AGC)$_{5}$
OF31	.	.	.	.	T	.	.	.	C	.	G	G	.	(TCC)$_{5}$	.	.	(AGC)$_{6}$
OF40	.	.	.	.	.	T	.	.	.	.	G	.	.	(TCC)$_{3}$	.	.	(AGC)$_{5}$
OF47	.	.	.	.	.	.	.	.	.	.	G	G	.	(TCC)$_{5}$	.	.	(AGC)$_{6}$
OF49	.	.	.	.	.	T	.	.	.	.	G	.	.	(TCC)$_{3}$	.	.	(AGC)$_{5}$
OF74	.	.	.	.	.	T	.	.	.	.	G	.	.	(TCC)$_{3}$	.	.	(AGC)$_{5}$
OF78	.	.	.	.	.	.	.	.	.	.	.	G	.	(TCC)$_{3}$	.	.	(AGC)$_{5}$
Dmad	A	.	.	.	.	.	.	.	C	.	G	G	.	(TCC)$_{4}$	.	.	(AGC)$_{5}$
Dgua	A	.	.	.	.	.	.	.	C	.	G	G	.	(TCC)$_{7}$	.	.	(AGC)$_{4}$AGT(AGC)$_{4}$

Region	E4																	
Position	9054	9142	9260	9283	9311	9326	9352	9388	9493	9596	9626	9628	9646	9685	9703	9901	9925	9995
	*		*		*	*				*	*							*
Chcu	G	A	G	C	C	T	C	C	T	C	C	T	C	G	G	G	T	G
OF01	.	.	C	.	G	.	A	.	.	.	.	.	.	A	.	.	C	.
OF07	.	.	C	.	G	.	.	.	.	.	T	A	.	.	.	.	C	T
OF14	A	C	C	.	G	A	.	.	.	.	.	.	.	.	.	.	.	.
OF15	.	.	.	.	G	.	.	.	.	.	.	.	T	.	.	.	C	.
OF18	.	C	C	.	G	.	.	.	.	T	.	.	.	.	.	.	.	.
OF21	.	C	C	.	G	.	.	.	.	.	.	.	.	.	.	.	C	.
OF23	A	T	C	T	G	.	.	.	.	.	.	.	.	.	.	.	C	.
OF28	A	C	C	.	G	.	.	.	C	.	.	.	.	.	A	.	C	.
OF31	A	.	C	.	G	.	.	.	.	.	.	.	.	.	.	.	.	.
OF40	.	C	C	.	G	.	.	.	.	.	.	A	.	.	.	.	C	.
OF47	A	.	C	.	.	.	.	.	.	.	.	.	.	.	.	.	.	.
OF49	.	C	.	.	G	.	.	A	.	.	.	.	.	.	.	A	.	.
OF74	.	C	C	.	G	.	.	.	.	T	.	.	.	.	.	.	.	.
OF78	.	C	C	.	G	.	.	.	.	.	.	.	.	.	.	.	C	.
Dmad	.	C	C	.	G	.	.	.	.	.	.	.	.	.	.	.	.	T
Dgua	.	C	C	.	G	.	.	.	.	.	.	.	T	.	.	.	.	T

Region	E4																							
Position	10023	10030	10035	10081	10099	10169	10231	10255	10284	10285	10287	10387	10498	10525	10564	10631	10762	10841	10844	10901	10906	10930	10969	10987
	*		*			*			*	*	*					*		*	*	*				
Chcu	A	G	A	A	C	T	T	A	T	G	C	C	C	T	C	G	G	A	C	C	A	G	G	C
OF01	.	A	.	.	.	.	.	.	.	.	.	.	.	G	.	.	A	.	.	.	.	.	.	.
OF07	G	.	.	.	.	.	.	.	.	.	.	.	.	G	.	.	A	.	.	.	.	.	.	.
OF14	.	.	.	.	A	.	.	.	.	.	.	.	.	G	.	.	A	.	.	.	.	A	.	.
OF15	.	.	C	.	.	.	.	.	.	.	.	.	.	.	T	A	A	.	.	.	.	.	.	T
OF18	.	.	.	.	.	.	.	.	A	T	T	A	.	.	.	.	A	.	T	.	.	.	.	.
OF21	G	.	.	.	.	.	.	.	.	.	.	.	.	G	.	.	A	.	.	.	T	.	.	.
OF23	G	.	.	.	.	.	.	.	.	.	.	.	T	G	G	.	A	.	.	.	.	.	.	.
OF28	.	.	.	.	.	.	.	.	.	.	.	.	.	G	.	.	A	C	.	.	.	.	.	.
OF31	.	.	.	.	A	.	.	.	.	.	.	.	.	G	.	.	A	.	.	.	.	.	.	.
OF40	.	A	.	G	.	C	G	T	.	.	.	.	.	G	.	.	A	.	.	.	.	.	.	.
OF47	.	.	C	.	.	.	G	.	.	.	.	.	.	G	.	.	A	.	.	.	.	.	.	.
OF49	.	.	.	.	.	.	.	.	.	.	.	.	.	G	.	.	A	.	.	T	.	.	T	.
OF74	.	.	.	.	.	.	.	.	.	.	.	.	.	G	.	.	A	.	.	.	.	A	.	.
OF78	.	A	.	.	.	.	.	.	.	.	.	.	.	G	.	.	.	.	.	.	.	.	.	.
Dmad	.	.	.	.	.	.	A	.	.	.	.	.	.	.	.	.	A	.	.	.	.	.	.	.
Dgua	.	.	.	.	.	.	.	.	.	.	.	.	.	G	.	.	A	.	.	.	.	.	.	.

continued on next page

J) *Psc*

continued from previous page

	E4														3'																				
	10994	11020	11032	11046	11191	11251	11290	11476	11568	11581	11804	11854	11879	12013	12078	12121	12151	12190	12191	12200	12207	12209	12341	12372	12373	12393	12398	12425	12432	12532	12533	12551	12554	12595	12627
	*			*							*		*																						
Chcu	G	C	C	C	C	C	G	G	A	G	A	G	A	C	A	T	(CAG)$_4$CAACAG	G	A	A	C	C	G	C	C	T	C	A	A	A	T	A	A	A	T
OF01	.	T	.	.	T	.	.	C	.	.	G	.	.	T	.	.	(CAG)$_6$	C	.	T	.	.	.	.	.	G	.	.	.	.	.	.	.	.	.
OF07	A	T	.	.	.	.	.	C	.	.	.	.	.	T	.	.	(CAG)$_2$CAACAGCAACGG	C	.	T	.	.	.	.	.	G	.	.	d_7	.	.	.	C	.	C
OF14	.	.	.	.	.	G	.	C	.	.	.	.	.	T	.	.	(CAG)$_6$	C	.	T	.	.	-	.	.	.	G	.	d_7	.	.	.	.	.	.
OF15	.	T	.	.	.	.	.	.	.	.	.	A	.	T	.	.	(CAG)$_3$CAACAGCAACAT	C	.	T	.	.	.	.	.	G	.	G	d_7	.	.	.	.	T	.
OF18	.	T	.	.	.	.	.	C	.	.	.	.	.	T	G	.	(CAG)$_4$CAACAG	C	.	T	.	.	.	.	.	.	.	.	.	.	.	.	C	.	.
OF21	.	T	.	.	.	.	.	T	.	.	.	.	.	T	.	.	(CAG)$_4$CAA(CAG)$_2$CAACAG	C	.	T	T	T	.	.	.	.	.	.	d_7	.	.	.	G	.	.
OF23	A	T	.	.	.	.	.	C	.	.	.	.	.	T	.	.	(CAG)$_4$CAACAG	C	G	T	.	.	.	.	.	.	.	.	.	.	.	.	G	.	.
OF28	.	T	.	.	.	.	.	.	.	T	.	.	.	T	.	.	(CAG)$_4$CAACAG	C	.	T	.	.	.	.	.	.	.	.	d_7	.	.	.	G	.	.
OF31	.	T	.	.	.	.	.	.	.	.	.	.	G	T	.	.	(CAG)$_6$	C	.	T	.	.	.	.	.	.	.	.	.	.	.	.	C	.	.
OF40	.	T	T	G	.	.	.	.	.	.	.	.	.	T	.	.	(CAG)$_4$CAACAG	C	.	T	.	.	.	A	T	.	.	.	d_{14}	.	.	.	.	.	.
OF47	.	T	.	.	.	.	.	.	d_9	.	.	.	.	T	.	.	(CAG)$_3$(CAACAG)$_2$	C	.	T	.	.	.	.	.	.	.	.	d_7	.	.	C	.	.	.
OF49	.	T	.	.	.	.	.	.	.	.	.	.	.	T	.	C	(CAG)$_2$CAACAG	C	.	T	.	.	.	.	.	.	.	.	.	T	A	.	.	.	.
OF74	.	.	.	.	.	.	A	C	.	.	.	.	.	T	.	.	(CAG)$_7$	C	.	T	.	.	.	.	.	.	.	.	.	.	.	.	C	.	.
OF78	.	T	.	.	.	.	.	C	.	.	.	.	.	T	.	.	(CAG)$_6$	C	.	T	.	.	.	.	.	G	.	.	d_7	.	.	.	C	.	.
Dmad	.	.	.	.	.	.	.	.	.	.	.	.	.	T	.	.	CAGCAT(CAG)$_2$CAACAG	C	.	T	.	.	.	.	.	.	.	.	d_7	.	.	G	.	.	.
Dgua	.	.	.	.	.	.	.	.	d_9	.	.	.	.	T	.	.	CAGCAC(CAG)$_3$	C	G	T	.	.	.	.	.	.	A	.	.	.	.	.	.	.	.

K) *Sce*

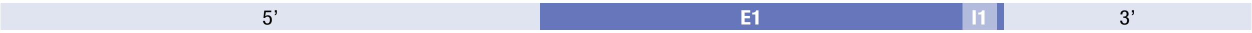

	10	111	126	157	432	509	573	768	782	783	810	836	870	1116	1125	1192	1205	1236	1384	1401	1423	1430	1431	1448	1459	1482	1694	1763	1799	2024	2057	2060
OF07	A	A	G	T	T	A	-	T	G	G	C	T	C	A	T	A	C	A	A	T	A	A	A	C	T	A	A	A	G	T	A	C
OF16	.	.	.	.	.	.	i_{149}	G	.	.	G	.	T	-	d_{19}	.	T	.	.	.	.	G	T	T	.	T	C	.	.	.	C	T
OF18	.	.	.	.	C	.	i_{176}	G	.	.	G	.	T	.	d_{19}	G	.	G	G	.	.	G	.	T	.	T	.	G	.	C	C	.
OF21	.	.	.	.	C	.	i_{176}	G	.	.	G	.	T	-	d_{19}	.	T	.	G	.	.	.	.	T	A	T	.	G	.	.	C	.
OF28	.	.	.	.	.	.	-	.	.	.	.	.	T	.	.	.	.	.	.	.	.	.	.	.	.	T	.	G	.	.	C	.
OF44	.	.	.	.	.	.	i_{168}	G	.	.	G	A	T	.	d_{19}	G	.	G	G	.	.	G	.	T	.	T	.	G	.	.	C	.
OF47	.	.	A	A	.	.	-	.	.	.	.	.	T	.	.	.	.	.	.	.	.	.	.	T	.	T	.	G	A	C	C	.
OF58	G	C	.	.	.	.	i_{177}	G	.	.	G	.	T	-	d_{19}	G	.	G	G	.	-	G	.	T	.	T	.	G	.	.	C	.
OF78	.	.	A	.	.	d_6	-	.	C	T	.	.	T	.	d_{19}	G	.	.	.	G	.	.	.	.	.	T	.	.	.	.	.	.
Dmad	.	.	.	.	.	.	-	.	.	.	.	.	T	.	.	.	.	.	.	.	C	G	.	T	.	T	.	G	.	.	C	.
Dgua	.	.	.	.	.	.	-	G	.	.	.	.	-	.	.	.	.	.	.	.	C	G	.	T	.	T	C	G	.	.	C	.

	2073	2081	2127	2171	2277	2278	2432	2531	2540	2684	2777	2914	2915	3003	3006	3106	3130	3156	3158	3212	3233	3236	3252	3308	3347	3369
			*		*	*																				
OF07	$(GGA)_5$	A	G	G	G	G	G	T	A	G	A	G	C	A	A	-	A	T	G	T	C	A	i_3	G	A	C
OF16	$(GGA)_5$	T	.	C	.	.	.	.	T	T	.	.	.	.	.	-	.	.	.	.	.	.	i_3	.	.	.
OF18	$GGAGGT(GGA)_3$	T	.	C	T	T	.	.	.	.	.	.	.	.	.	-	.	C	T	G	.	T	-	A	T	.
OF21	$(GGA)_6$	.	.	.	.	.	.	.	.	.	.	.	.	.	.	-	.	.	T	G	.	T	-	A	T	.
OF28	$(GGA)_6$	.	A	.	.	.	.	.	.	.	.	.	.	.	T	-	.	.	T	G	.	T	-	A	G	.
OF44	$(GGA)_6$	.	.	.	.	.	T	C	.	.	.	.	.	.	.	-	C	.	.	G	T	T	-	A	G	.
OF47	$(GGA)_5$	.	.	.	.	.	.	.	T	T	.	.	.	G	.	-	.	.	.	.	.	.	i_3	.	.	.
OF58	$GGAGGT(GGA)_3$	T	.	C	.	.	.	.	.	.	G	A	A	.	.	i_6	.	.	T	G	.	T	-	A	.	G
OF78	$(GGA)_5$	.	.	.	.	.	T	C	.	.	.	.	.	.	.	-	.	.	T	G	.	T	-	A	.	.
Dmad	$(GGA)_4$	.	.	.	.	.	.	.	.	.	.	A	.	.	.	-	.	.	T	G	.	.	-	.	.	.
Dgua	$(GGA)_5$	.	.	.	.	.	.	.	.	.	.	A	.	.	.	i_3	.	.	T	G	.	.	-	.	.	T

L) *Pc*

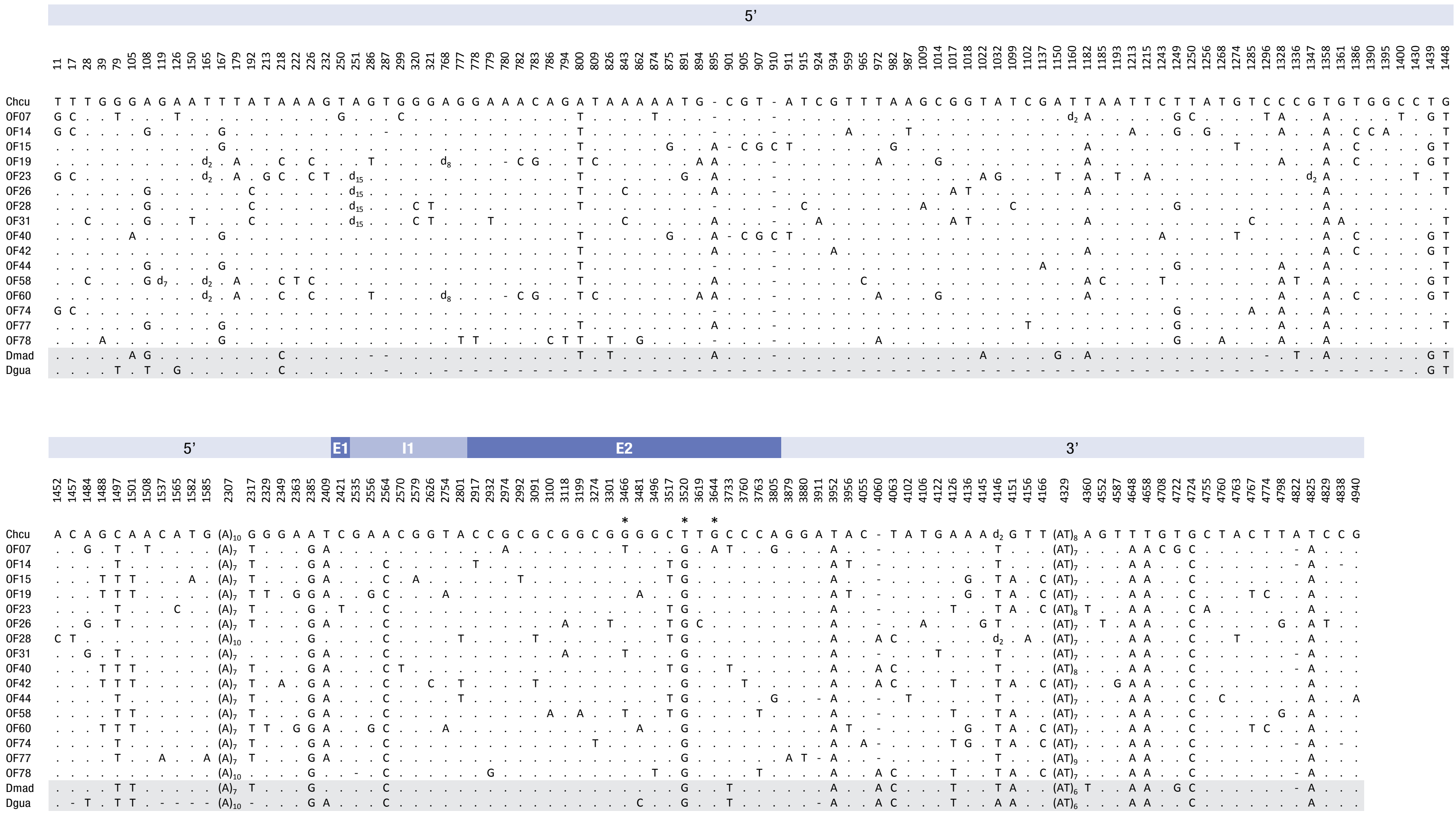

5'
Chcu
OF07
OF14
OF15
OF19
OF23
OF26
OF28
OF31
OF40
OF42
OF44
OF58
OF60
OF74
OF77
OF78
Dmad
Dgua
5'
E1
I1
E2
3'

M) *Ph-p*

5’ I1

	28	56	80	95	96	124	218	225	348	350	401	439	478	508	554	634	651	673	730	859	966	967	977	981	983	1000	1128	1133	1145	1165	1178	1210	1215	1243	1252	1349	1390	1430	1477	1493	1516	1534	1544	1547	1568	1632	1637
Chcu	T	T	G	C	G	A	T	T	C	T	A	G	C	A	T	G	T	A	T	A	T	G	-	A	A	T	A	G	C	A	T	G	T	T	T	C	A	T	C	C	G	G	T	C	T	C	G
OF07	G	.	.	.	.	.	.	.	.	.	.	.	.	.	-	.	.	G	.	.	C	.	-	.	.	.	C	A	.	C	.	.	.	.	.	.	G	.	.	.	A	.	.	.	.	.	.
OF14	G	.	.	.	.	.	.	.	.	.	.	.	.	.	-	.	C	G	.	.	C	.	-	.	.	.	C	A	.	.	.	A	.	.	.	.	G	.	.	.	.	.	.	.	.	.	.
OF15	G	.	.	.	.	.	.	.	.	.	.	.	.	.	-	.	.	G	.	.	C	.	i_3	G	G	.	C	A	.	.	.	.	.	.	.	.	G	.	.	.	.	.	.	.	.	.	.
OF16	G	.	.	.	.	.	.	.	.	.	T	.	.	.	-	T	.	G	.	G	.	.	-	.	.	.	C	A	.	.	.	.	.	.	.	-	G	.	.	.	.	.	.	.	.	.	A
OF18	G	.	.	.	.	.	.	.	.	.	.	A	.	.	.	.	.	.	.	.	.	.	-	.	.	.	C	A	.	.	.	.	.	.	.	.	G	.	.	.	.	.	.	.	.	.	.
OF19	G	.	.	.	.	.	.	.	.	.	.	.	.	.	-	.	.	G	.	.	C	.	-	.	.	.	C	A	.	.	.	.	.	.	.	.	G	.	.	.	A	.	.	.	.	.	.
OF23	G	.	.	T	.	T	.	.	.	.	.	.	.	.	.	.	.	G	.	.	.	.	-	.	.	.	C	A	.	.	.	.	.	.	A	.	G	.	.	.	.	.	.	.	.	.	.
OF28	G	.	.	.	.	.	.	.	A	-	.	.	.	.	.	.	.	.	.	.	.	.	-	.	.	.	.	A	.	.	A	.	.	.	.	.	G	.	.	.	.	A	.	A	.	T	.
OF40	G	.	T	.	.	.	.	.	.	.	.	.	.	.	.	.	.	.	.	.	.	.	-	.	.	.	.	A	.	C	.	.	.	.	.	.	G	.	T	.	.	.	.	.	d_{10}	.	.
OF42	G	.	.	.	.	.	.	.	.	.	.	.	.	.	-	.	.	G	.	.	C	.	-	.	.	.	C	A	.	.	.	.	.	.	.	.	G	.	.	.	.	.	.	.	.	.	.
OF44	G	.	T	.	A	.	.	.	.	.	.	.	.	.	-	.	.	G	.	.	.	.	-	.	.	.	C	A	.	.	.	.	.	.	.	.	G	.	.	T	.	A	G	.	.	.	.
OF47	G	.	.	.	.	.	.	.	.	.	T	.	.	.	-	T	.	G	.	G	.	T	-	.	.	.	C	A	.	.	.	.	.	.	.	-	G	.	.	.	.	.	.	.	.	.	A
OF49	G	A	.	.	.	.	.	.	.	.	.	.	.	.	.	.	.	G	.	.	.	.	-	.	.	-	C	A	A	.	.	.	C	.	.	.	G	.	.	.	.	A	.	.	.	.	.
OF58	G	.	.	.	.	.	.	A	.	.	.	.	.	.	-	.	.	G	.	.	.	.	-	.	.	.	C	A	.	.	.	.	.	.	.	.	G	C	.	.	.	.	.	.	.	.	.
OF60	G	.	.	.	.	.	.	.	.	.	.	.	.	.	-	.	.	G	.	.	C	.	-	.	.	.	C	A	.	.	.	.	.	.	.	.	G	.	.	.	A	.	.	.	.	.	.
OF74	G	A	.	.	.	.	A	.	.	.	.	.	A	G	.	.	.	G	G	.	.	.	-	.	.	.	C	A	.	.	.	.	.	C	.	.	G	.	.	.	.	A	.	.	.	.	.
Dmad	G	.	.	.	.	.	.	.	.	.	.	.	.	.	-	.	.	G	.	.	.	.	-	.	.	.	-	A	A	.	.	.	.	.	.	.	G	.	.	.	.	.	.	.	.	.	.
Dgua	?	?	.	.	.	.	.	.	.	-	.	.	.	.	.	.	.	G	.	.	.	.	-	.	.	.	-	A	A	C	.	.	.	C	.	T	G	.	.	.	.	.	.	.	.	.	.

	1687	1695	1767	1883	1887	1892	1899	1920	1935	1946	1987	1998	2070	2101	2105	2114	2141	2163	2165	2167	2168	2170	2181	2196	2271	2346	2436	2499	2518	2593	2598	2629	2638	2644	2648	2652	2689	2719	2732	2778	2810	2817	2821	2826	2839	2925
Chcu	C	A	A	T	T	C	G	C	A	C	G	A	G	A	-	C	T	C	G	A	G	T	G	C	T	G	C	A	A	T	G	G	C	A	A	G	A	A	G	G	T	C	-	-	G	T
OF07	.	G	.	.	.	.	.	A	T	G	.	G	A	.	-	.	.	.	.	.	A	G	.	.	.	.	T	.	.	.	.	.	.	.	.	.	T	.	.	.	.	T	-	A	.	.
OF14	.	G	.	A	.	.	.	A	.	G	.	.	A	.	-	.	A	.	.	.	.	G	.	.	.	.	T	.	.	.	.	.	A	.	.	.	T	.	.	.	.	T	-	A	.	.
OF15	.	G	.	.	.	.	.	A	.	G	.	.	A	.	-	T	.	.	.	.	.	G	.	.	.	.	T	.	.	.	.	.	.	.	.	.	T	.	.	.	.	T	-	A	.	.
OF16	T	G	.	.	A	.	.	.	.	.	A	.	A	.	-	.	.	.	.	.	.	G	.	.	A	.	T	.	.	.	.	.	.	.	.	.	.	.	d_4	A	.	T	i_4	A	.	.
OF18	.	.	.	.	.	.	.	.	.	.	.	.	.	.	-	.	.	T	.	.	.	G	.	.	.	T	T	.	.	.	.	.	.	.	.	.	.	.	.	.	.	T	-	-	.	.
OF19	.	G	.	.	.	.	.	A	T	G	.	G	A	.	-	.	.	.	.	.	.	G	C	.	.	.	T	.	.	.	.	.	.	.	.	.	T	.	.	.	.	T	-	-	.	.
OF23	.	G	.	.	A	.	.	.	.	.	.	.	A	.	-	.	.	.	.	.	.	G	.	.	.	.	T	.	C	.	.	.	.	.	T	.	T	.	.	.	.	T	-	-	.	.
OF28	.	G	d_2	.	A	G	.	.	.	.	.	.	A	.	-	.	.	.	.	.	.	G	.	.	.	.	T	.	.	.	.	T	.	G	.	.	.	.	.	.	.	T	-	A	.	.
OF40	.	.	.	.	.	.	.	A	.	G	.	.	A	.	-	.	.	.	.	.	.	G	.	.	.	.	T	.	.	.	.	.	.	.	.	T	T	.	.	.	.	T	-	A	.	A
OF42	.	G	.	.	.	.	.	A	.	.	.	.	A	-	-	.	.	.	.	.	.	G	.	A	.	.	T	.	.	.	.	.	.	.	.	.	.	.	.	.	.	T	-	-	.	.
OF44	.	G	.	.	.	.	.	.	.	.	.	.	A	.	i_4	.	.	.	.	.	.	G	.	.	.	.	T	G	.	.	.	.	.	.	.	.	.	G	.	.	.	T	-	A	.	.
OF47	.	G	.	.	A	.	.	.	.	.	A	.	A	.	-	.	.	.	.	C	.	G	.	.	A	.	T	.	.	.	.	.	.	.	.	.	.	.	d_4	A	.	T	i_4	A	.	.
OF49	.	G	.	.	A	.	C	.	.	.	.	.	A	.	-	.	.	.	.	.	.	G	.	.	.	.	T	.	.	.	.	.	.	.	.	.	.	.	.	.	.	T	-	A	.	.
OF58	.	.	.	.	A	G	.	.	.	.	.	.	A	.	-	.	.	.	.	.	.	G	.	.	.	.	T	.	.	C	.	.	.	.	.	.	.	.	.	.	.	T	-	-	C	.
OF60	.	G	.	.	.	.	.	A	T	G	.	G	A	.	-	.	.	.	.	.	.	G	C	.	.	.	T	.	.	.	.	.	.	.	.	.	T	.	.	.	.	T	-	-	.	.
OF74	.	G	.	.	A	G	.	.	.	.	.	.	A	.	-	.	.	.	C	.	T	G	.	.	.	.	T	.	.	.	A	.	.	.	.	.	.	.	.	.	G	T	-	-	.	.
Dmad	.	G	.	.	-	.	.	.	.	.	.	.	A	.	-	.	.	.	.	.	.	G	.	.	.	.	T	.	.	.	.	.	.	.	.	.	.	.	.	.	.	T	-	-	.	.
Dgua	.	G	.	.	A	.	T	.	.	.	.	.	A	.	-	.	.	.	A	.	.	G	.	.	.	.	T	.	.	.	.	.	.	.	.	.	.	.	A	.	.	T	-	A	-	.

I1 E2 I2 E3 I3 E4

	2960	3136	3196	3233	3258	3266	3302	3410	3482	3489	3498	3518	3523	3533	3549	3563	3575	3577	3586	3668	3753	3774	3794	3805	3818	3827	3863	3945	4074	4290	4337	4374	4434	4440	4466	4512	4538	4586	4601	4635	4649	4736	4754	4766	4808	4838	4898
																						*		*				*	*											*							
Chcu	$(CT)_8$	T	G	G	C	C	G	$(TC)_5$	T	C	G	T	-	C	T	A	A	G	C	C	-	A	G	G	G	G	C	A	A	A	C	C	G	G	C	C	C	G	A	A	C	C	G	C	G	C	G
OF07	$(CT)_8$	G	.	.	.	A	.	$(TC)_5$	.	.	.	.	C	.	.	T	.	.	.	.	-	.	.	.	.	.	.	T	.	.	.	.	.	.	.	d_6	.	.	.	.	T	.	.	.	.	T	.
OF14	$(CT)_7$	G	.	.	.	.	.	$(TC)_6$	.	.	.	.	C	.	.	T	.	.	.	.	i_3	.	.	.	.	.	.	.	.	.	.	.	d_6	.	.	.	.	A	C	.	.	.	.	A	.	.	.
OF15	$(CT)_8$	G	.	A	.	.	.	$(TC)_5$	G	.	.	.	C	.	.	T	.	.	.	.	-	.	.	.	.	.	.	.	.	.	.	.	.	d_6	.	.	T	.	.	.	T	.	.	.	.	.	.
OF16	$(CT)_9$	G	.	.	.	.	.	$(TC)_5$	.	.	.	C	C	.	.	T	G	.	.	.	-	.	.	A	.	.	.	.	.	.	.	T	.	.	.	.	.	.	.	.	T	.	.	.	T	.	.
OF18	$(CT)_7$	.	.	.	d_8	.	.	$(TC)_5$	.	.	.	C	C	.	.	T	G	.	.	.	-	C	.	A	.	.	.	.	.	.	.	.	.	.	.	.	T	.	.	.	T	.	.	A	.	.	A
OF19	$(CT)_8$	.	.	.	.	.	.	$(TC)_5$	.	.	.	.	C	.	.	T	.	.	.	.	i_{15}	.	.	.	.	A	.	.	.	d_3	.	.	.	.	.	.	.	.	.	.	.	.	.	.	.	.	.
OF23	$(CT)_8$	.	.	.	.	.	C	$(TC)_5$	.	.	.	.	C	.	.	T	.	.	.	.	-	.	.	.	.	.	.	.	.	.	.	T	.	.	.	.	.	.	C	C	.	.	.	A	.	.	.
OF28	$(CT)_9$	.	.	.	.	.	.	$(TC)_5$	.	.	.	.	C	.	.	T	.	.	.	.	-	.	.	.	.	.	.	.	T	.	.	.	.	.	.	.	.	.	.	.	T	.	.	.	T	.	.
OF40	$(CT)_8$	G	.	.	.	.	.	$(TC)_5$	.	.	.	.	C	.	.	T	.	A	.	.	-	.	.	.	.	.	.	.	.	.	.	.	.	.	.	.	.	.	.	.	T	.	A	.	.	.	.
OF42	$(CT)_8$	.	T	.	.	.	.	$(TC)_5$	.	.	.	C	C	.	.	T	G	.	.	.	-	.	.	.	.	.	.	.	.	.	.	.	.	.	.	.	.	.	.	.	T	T	.	.	.	T	.
OF44	$(CT)_7$	.	.	.	.	.	.	$(TC)_5$	.	T	.	.	C	A	A	T	.	.	A	.	-	.	A	.	.	.	.	.	.	.	T	.	.	.	G	.	.	A	C	.	.	.	.	.	.	.	.
OF47	$(CT)_{10}$	G	.	.	.	.	.	$(TC)_5$	.	.	.	C	C	.	.	T	G	.	.	.	-	.	.	A	.	.	.	.	.	.	.	T	.	.	.	.	.	.	C	.	T	.	.	A	.	.	.
OF49	$(CT)_{10}$	.	.	.	.	.	.	$(TC)_5$	.	.	A	C	C	.	.	T	.	.	.	.	-	.	.	.	A	.	T	.	.	.	.	.	.	.	.	.	.	.	.	.	T	T	.	.	.	T	.
OF58	$(CT)_7$	.	.	.	.	.	.	$(TC)_5$	.	T	.	.	C	A	.	T	.	.	A	.	-	.	A	.	.	.	.	.	.	.	T	.	.	.	G	.	.	A	C	.	.	.	.	A	.	.	.
OF60	$(CT)_8$	.	.	.	.	.	.	$(TC)_5$	.	.	.	.	C	.	.	T	.	.	.	.	i_{15}	.	.	.	.	A	.	.	.	d_3	.	.	.	.	.	.	.	.	.	.	.	.	.	.	.	.	.
OF74	$(CT)_8$	.	.	.	.	.	.	$(TC)_6$	.	.	.	.	C	.	.	T	.	.	.	T	-	.	.	.	.	.	.	.	.	.	.	.	.	.	.	.	.	.	.	.	T	.	A	.	.	.	.
Dmad	$(CT)_8$	A	.	.	T	.	.	$(TC)_5$	.	.	.	.	C	.	.	T	.	.	.	.	-	.	.	.	.	.	A	.	.	.	.	.	.	.	.	.	.	.	.	.	.	.	.	.	.	.	.
Dgua	$(CT)_{11}$	.	.	.	.	.	.	$(TC)_{10}$	-	.	.	.	C	.	.	T	.	.	.	.	-	.	.	.	.	A	.	.	.	.	.	.	.	.	.	.	.	.	.	.	.	.	.	.	.	.	T

	4977	5030	5198	5213	5238	5488	5489	5610	5691	5889	5987	6044	6151	6259	6297	6311	6322	6532	6727	6870	6931	7103	7114	7245	7392	7417	7432
													*									*					
Chcu	$(CAG)_7$	G	C	A	G	C	i_6	$(CAR)_{16}$	$(CAR)_{13}$	$(CAR)_8$	G	G	G	A	A	C	C	$(CAR)_{27}$	G	G	$(CAR)_{14}$	G	$(CAR)_9$	C	C	-	C
OF07	$(CAG)_7$	.	.	.	.	T	i_6	$(CAR)_{16}$	$(CAR)_{17}$	$(CAR)_{10}$	.	.	.	G	T	.	.	$(CAR)_{25}$	.	.	$(CAR)_{21}$	T	$(CAR)_8$	.	.	i_6	.
OF14	$(CAG)_5$	.	.	.	.	.	i_6	$(CAR)_{16}$	$(CAR)_{25}$	$(CAR)_8$	.	.	.	.	.	.	.	$(CAR)_{24}$	.	.	$(CAR)_{21}$	.	$(CAR)_8$	.	.	-	.
OF15	$(CAG)_7$	.	A	.	.	T	i_{12}	$(CAR)_{16}$	$(CAR)_{21}$	$(CAR)_{10}$	.	.	.	G	.	.	.	$(CAR)_{24}$	.	.	$(CAR)_{21}$	.	$(CAR)_8$	.	.	-	.
OF16	$(CAG)_5$	A	.	.	.	T	i_{12}	$(CAR)_{16}$	$(CAR)_{25}$	$(CAR)_8$	A	A	.	G	T	.	A	$(CAR)_{22}$	d_6	.	$(CAR)_{21}$	.	$(CAR)_8$	.	.	-	.
OF18	$(CAG)_7$	.	.	G	.	.	i_6	$(CAR)_{18}$	$(CAR)_{17}$	$(CAR)_{10}$	.	.	.	.	.	.	.	$(CAR)_{23}$	.	.	$(CAR)_{21}$	.	$(CAR)_{10}$	.	.	i_6	.
OF19	$(CAG)_5$	.	.	.	.	T	i_{12}	$(CAR)_{17}$	$(CAR)_{25}$	$(CAR)_{10}$	.	.	C	.	.	.	.	$(CAR)_{28}$	.	.	$(CAR)_{21}$	.	$(CAR)_8$	.	.	-	.
OF23	$(CAG)_7$	.	.	.	.	T	i_6	$(CAR)_{17}$	$(CAR)_{29}$	$(CAR)_{10}$	.	.	.	G	T	.	.	$(CAR)_{22}$	.	.	$(CAR)_{21}$	.	$(CAR)_9$	.	.	-	.
OF28	$(CAG)_7$	.	.	.	.	.	i_{12}	$(CAR)_{18}$	$(CAR)_{21}$	$(CAR)_8$	.	.	.	G	T	.	.	$(CAR)_{22}$	d_6	A	$(CAR)_{14}$	.	$(CAR)_6$	A	.	i_6	.
OF40	$(CAG)_7$	.	.	.	.	.	i_6	$(CAR)_{19}$	$(CAR)_{21}$	$(CAR)_7$	.	.	.	G	T	.	.	$(CAR)_{25}$	.	.	$(CAR)_{20}$	.	$(CAR)_8$	.	.	-	.
OF42	$(CAG)_6$	.	.	.	.	.	i_6	$(CAR)_{17}$	$(CAR)_{21}$	$(CAR)_{10}$	.	.	.	G	T	.	.	$(CAR)_{23}$	.	.	$(CAR)_{18}$	.	$(CAR)_8$	.	.	-	.
OF44	$(CAG)_6$	.	.	.	.	.	i_6	$(CAR)_{16}$	$(CAR)_{21}$	$(CAR)_{10}$	.	.	.	.	.	.	.	$(CAR)_{25}$	.	.	$(CAR)_{21}$	.	$(CAR)_8$	.	.	-	.
OF47	$(CAG)_7$	.	.	.	d_6	T	i_{12}	$(CAR)_{16}$	$(CAR)_{13}$	$(CAR)_{10}$	.	.	.	.	.	.	.	$(CAR)_{25}$	.	.	$(CAR)_{21}$	.	$(CAR)_9$	.	T	-	.
OF49	$(CAG)_7$	.	.	G	.	.	i_6	$(CAR)_{18}$	$(CAR)_{21}$	$(CAR)_{10}$	.	.	.	.	.	T	.	$(CAR)_{25}$	.	.	$(CAR)_{21}$	.	$(CAR)_8$	.	.	-	.
OF58	$(CAG)_6$	.	.	.	.	.	i_6	$(CAR)_{16}$	$(CAR)_{21}$	$(CAR)_{10}$	.	.	.	.	.	.	.	$(CAR)_{24}$	.	.	$(CAR)_{21}$	T	$(CAR)_8$	.	.	-	d_6
OF60	$(CAG)_5$	.	.	.	.	T	i_{12}	$(CAR)_{17}$	$(CAR)_{25}$	$(CAR)_{10}$	.	.	C	.	.	.	.	$(CAR)_{28}$	.	.	$(CAR)_{21}$	.	$(CAR)_8$	.	.	-	.
OF74	$(CAG)_7$	.	.	.	.	.	i_6	$(CAR)_{18}$	$(CAR)_{17}$	$(CAR)_{14}$	.	.	.	.	.	.	.	$(CAR)_{25}$	.	.	$(CAR)_{21}$	.	$(CAR)_8$	.	.	-	.
Dmad	$(CAG)_8$	.	.	G	d_6	.	i_6	$(CAR)_{15}$	$(CAR)_{29}$	$(CAR)_5$	.	.	.	.	.	.	.	$(CAR)_{26}$	.	.	$(CAR)_{17}$	.	$(CAR)_7$	.	.	-	.
Dgua	$(CAG)_7$	.	.	G	.	.	-	$(CAR)_{13}$	$(CAR)_{25}$	$(CAR)_8$	.	.	.	.	.	.	.	$(CAR)_{18}$	.	A	$(CAR)_{24}$	.	$(CAR)_5CCA(CAR)_4$	.	.	-	.

continued on next page

M) *Ph-p*

continued from previous page

	7438	7444	7521	7537	7599	7626	7659	7758	7910	8026	8029	8047	8059	8081	8093	8146	8151	8190
Region	E4																	
									*					*	*	*	*	
Chcu	-	(CAR)$_{13}$	A	(CAR)$_{9}$	A	G	C	G	G	A	(AGC)$_{3}$	A	A	C	A	T	C	C
OF07	-	(CAR)$_{13}$	.	(CAR)$_{9}$	.	.	.	.	.	.	(AGC)$_{3}$	.	.	.	.	A	.	T
OF14	-	(CAR)$_{13}$	.	(CAR)$_{13}$	C	T	.	.	.	.	(AGC)$_{3}$	.	.	.	.	A	G	.
OF15	-	(CAR)$_{13}$	.	(CAR)$_{12}$	.	.	.	.	.	.	(AGC)$_{3}$	.	.	.	.	A	.	.
OF16	-	(CAR)$_{13}$	.	(CAR)$_{9}$	.	.	.	.	.	.	(AGC)$_{3}$	.	.	.	.	A	.	.
OF18	-	(CAR)$_{13}$	.	(CAR)$_{9}$	.	.	.	.	.	d$_{3}$	(AGC)$_{4}$	.	.	.	G	A	.	T
OF19	-	(CAR)$_{14}$	.	(CAR)$_{12}$	.	.	.	.	.	.	(AGC)$_{3}$	d$_{6}$	.	T	.	A	.	T
OF23	-	(CAR)$_{13}$	.	(CAR)$_{12}$	.	.	.	.	.	.	(AGC)$_{3}$	.	.	.	.	.	.	.
OF28	-	(CAR)$_{13}$	.	(CAR)$_{9}$	.	.	.	.	.	.	(AGC)$_{3}$	.	.	.	.	A	.	T
OF40	-	(CAR)$_{13}$	.	(CAR)$_{6}$	.	.	.	A	C	d$_{3}$	(AGC)$_{4}$	.	.	.	.	A	.	.
OF42	-	(CAR)$_{13}$	T	(CAR)$_{6}$	C	.	.	.	.	.	(AGC)$_{1}$	.	d$_{9}$	.	.	A	.	.
OF44	-	(CAR)$_{13}$	.	(CAR)$_{12}$	.	.	.	.	.	.	(AGC)$_{5}$	.	.	.	.	A	.	T
OF47	i$_{6}$	(CAR)$_{13}$	.	(CAR)$_{9}$	.	.	.	.	.	.	(AGC)$_{3}$	.	.	.	.	A	G	.
OF49	-	(CAR)$_{13}$	.	(CAR)$_{9}$	.	.	.	.	.	d$_{3}$	(AGC)$_{2}$	.	.	.	.	A	.	T
OF58	-	(CAR)$_{13}$	.	(CAR)$_{12}$	.	.	T	.	.	.	(AGC)$_{3}$	.	.	.	.	A	.	.
OF60	-	(CAR)$_{14}$	.	(CAR)$_{12}$	.	.	.	.	.	.	(AGC)$_{3}$	d$_{6}$	.	T	.	A	.	T
OF74	-	(CAR)$_{14}$	.	(CAR)$_{9}$	.	.	.	.	.	d$_{3}$	(AGC)$_{4}$	.	.	.	.	A	.	.
Dmad	-	(CAR)$_{14}$	.	(CAR)$_{9}$	.	.	.	.	.	.	(AGC)$_{5}$	.	.	.	.	A	.	.
Dgua	i$_{6}$	(CAR)$_{7}$	.	(CAR)$_{14}$	.	A	.	.	.	d$_{3}$	(AGC)$_{4}$	.	.	.	.	A	.	.

	8225	8227	8241	8274	8283	8328	8342	8386	8580	8678	8743	8860	8862	8876
Region	E4								I4	E5				
	*	*				*	*				*		*	
Chcu	T	A	G	G	A	C	C	d$_{9}$	C	A	A	C	C	(GTGGGA)$_{3}$
OF07	C	.	.	T	.	.	.	d$_{9}$	.	.	C	A	.	(GTGGGA)$_{3}$
OF14	C	G	A	.	.	.	.	A	.	.	C	.	.	(GTGGGA)$_{2}$
OF15	C	.	.	.	.	.	.	A	.	.	C	A	.	(GTGGGA)$_{3}$
OF16	C	.	.	.	.	.	.	d$_{9}$	.	.	C	A	.	(GTGGGA)$_{3}$
OF18	C	.	.	.	.	.	.	d$_{9}$	.	.	C	A	.	(GTGGGA)$_{4}$
OF19	C	.	.	T	.	.	.	A	.	.	C	A	.	(GTGGGA)$_{3}$
OF23	C	.	A	.	.	.	.	d$_{9}$	.	.	C	A	.	(GTGGGA)$_{2}$
OF28	C	.	.	.	.	.	T	A	.	.	C	A	.	(GTGGGA)$_{2}$
OF40	C	.	.	.	.	.	.	d$_{9}$	.	.	C	A	.	(GTGGGA)$_{2}$
OF42	C	.	.	.	.	.	.	A	.	.	C	A	.	(GTGGGA)$_{2}$
OF44	C	.	A	.	.	.	.	d$_{9}$	.	.	C	A	T	(GTGGGA)$_{3}$
OF47	C	.	.	.	.	.	.	d$_{9}$	.	.	C	A	.	(GTGGGA)$_{2}$
OF49	C	.	.	T	.	A	.	d$_{9}$	.	.	C	A	.	(GTGGGA)$_{2}$
OF58	C	.	A	.	.	.	.	d$_{9}$	.	.	C	A	.	(GTGGGA)$_{2}$
OF60	C	.	.	T	.	.	.	A	.	.	C	A	.	(GTGGGA)$_{3}$
OF74	C	.	.	.	G	.	.	A	T	d$_{6}$	C	A	.	(GTGGGA)$_{3}$
Dmad	C	.	.	.	.	.	.	A	.	.	C	A	.	(GTGGGA)$_{1}$
Dgua	C	.	.	.	.	.	.	A	.	.	C	A	.	(GTGGGA)$_{1}$

	9092	9101	9107	9134	9217	9236	9280	9305	9317	9632	9665	9677	9746	9750	9771	9774	9808	9837	9842	9855	9960	9961	9981	10000	10010
Region	E5									3’															
		*	*	*		*																			
Chcu	i$_{6}$	C	G	G	T	T	T	T	T	-	G	A	T	A	G	G	T	G	C	G	C	C	T	A	T
OF07	i$_{6}$	.	.	.	G	.	A	.	.	-	A	d$_{6}$	.	G	.	.	.	T	.	A	.	.	.	.	.
OF14	i$_{6}$	.	.	.	G	.	A	.	.	-	A	d$_{6}$	.	G	.	.	.	.	.	.	.	.	.	.	.
OF15	-	.	C	.	G	.	.	.	.	-	A	d$_{6}$	.	G	.	C	.	T	.	.	.	.	.	.	.
OF16	i$_{6}$	G	C	.	G	.	.	d$_{6}$	.	-	A	d$_{6}$	.	G	.	.	.	T	.	A	.	.	C	.	.
OF18	i$_{6}$	.	C	.	G	.	A	.	.	-	A	d$_{6}$	.	G	.	.	.	T	.	A	.	.	.	.	.
OF19	i$_{6}$	.	.	.	G	.	A	.	.	-	A	d$_{6}$	.	G	.	.	.	.	.	.	.	.	.	.	C
OF23	i$_{6}$	.	.	C	G	.	.	.	.	-	.	.	.	G	.	.	.	.	.	.	.	.	.	.	.
OF28	i$_{6}$	.	C	.	G	.	A	d$_{6}$	.	-	A	d$_{6}$	G	G	.	.	A	.	T	.	.	.	.	.	.
OF40	i$_{6}$	.	.	.	G	.	.	.	.	-	A	d$_{6}$	G	G	.	.	A	.	T	.	.	.	.	.	C
OF42	i$_{6}$	.	.	C	G	C	.	d$_{6}$	d$_{6}$	-	A	d$_{6}$	.	G	.	.	.	T	.	A	.	.	.	.	.
OF44	i$_{6}$	.	.	.	G	.	.	.	.	-	A	d$_{6}$	.	G	.	.	.	.	.	.	.	.	.	.	.
OF47	-	.	C	.	G	.	A	d$_{6}$	.	-	A	d$_{6}$	.	G	.	.	.	.	.	.	.	.	.	T	.
OF49	i$_{6}$	.	.	.	G	.	.	.	.	i$_{12}$	A	.	.	G	.	.	.	.	.	.	A	T	.	.	.
OF58	i$_{6}$	.	.	.	G	.	.	.	.	-	.	.	.	.	.	.	.	.	.	.	.	.	.	.	.
OF60	i$_{6}$	.	.	.	G	.	A	.	.	-	A	d$_{6}$	.	G	.	.	.	.	.	.	.	.	.	.	C
OF74	i$_{6}$	.	.	.	G	.	.	.	.	-	A	d$_{6}$	.	G	A	.	.	.	.	.	.	.	.	.	.
Dmad	-	-	C	.	G	.	A	.	.	-	.	.	.	G	.	.	.	.	.	.	.	.	.	.	.
Dgua	-	A	C	.	G	.	A	.	.	-	.	.	.	G	A	T	.	.	.	.	.	.	.	.	.

N) *Scm*

	5'											E1										I1							E2				
	5	61	113	121	144	253	283	313	338	372	413	528	572	678	741	768	825	889	897	957	963	1020	1042	1049	1052	1053	1060	1066	1136	1226	1271	1286	1307
													*					*															
Chcu	G	T	T	T	C	A	-	C	A	A	A	T	A	G	T	A	C	G	G	C	A	A	T	C	C	T	A	G	C	A	T	A	G
OF07	.	.	.	.	.	G	A	.	G	.	d_2	C	.	.	.	.	.	.	.	.	.	T	.	.	T	.	.	.	.	.	C	.	A
OF14	.	.	.	.	.	G	-	.	G	.	.	.	.	.	.	.	.	.	.	.	.	T	.	.	T	A	G	.	.	.	.	.	A
OF16	.	.	.	.	A	G	-	.	G	.	.	C	.	.	.	.	.	.	.	.	.	.	.	.	T	A	.	.	.	.	.	.	A
OF18	.	.	G	G	.	G	A	.	G	.	.	C	.	.	A	.	.	.	A	.	.	.	.	.	T	A	.	.	A	.	.	.	.
OF19	.	G	.	.	.	G	-	T	G	C	.	C	.	.	.	.	.	.	.	.	.	T	.	.	T	A	.	.	.	.	.	.	A
OF21	.	.	.	.	A	G	-	.	G	.	.	.	.	.	.	G	.	.	.	.	C	T	.	.	T	A	.	A	.	.	.	.	.
OF28	C	.	.	.	.	G	-	.	G	.	.	C	.	.	.	.	.	.	.	.	.	T	.	.	T	A	G	.	.	.	.	.	A
OF31	.	.	.	.	.	G	-	.	G	.	.	C	.	.	.	.	.	.	.	.	.	T	.	.	T	A	G	.	.	.	.	.	A
OF40	.	.	.	.	.	G	A	.	.	.	.	C	.	.	.	.	.	.	.	.	.	T	.	.	T	A	.	.	.	.	.	.	A
OF44	C	.	.	.	.	G	-	.	G	.	.	.	.	.	.	.	.	.	.	.	.	T	.	.	T	A	.	.	.	.	.	G	A
OF47	.	.	.	.	.	G	-	.	G	.	.	.	.	C	.	.	.	.	.	.	.	T	.	d_3	T	A	.	.	.	.	.	.	A
OF54	C	.	.	.	.	G	-	.	G	.	.	.	.	.	.	.	T	.	.	.	.	T	.	.	T	A	.	.	.	T	.	.	.
OF58	.	.	.	.	.	G	-	.	G	.	.	C	G	.	.	G	.	.	.	.	.	T	.	.	T	A	.	.	.	.	.	.	A
OF60	.	G	.	.	.	G	-	T	G	C	.	C	.	.	.	.	.	.	.	.	.	T	.	.	T	A	.	.	.	.	.	.	A
OF78	C	.	.	.	.	G	-	.	G	.	.	C	.	.	.	G	.	A	.	A	.	T	C	.	T	A	G	.	.	.	.	.	.
Dmad	.	.	G	G	.	G	A	.	G	.	.	.	.	.	C	.	.	.	.	.	.	.	.	.	T	A	.	.	.	.	.	.	.
Dgua	.	.	.	.	.	G	-	.	G	.	.	.	.	.	.	.	.	.	.	.	.	.	.	.	T	A	.	.	.	.	.	.	.

	E2														I2															
	1310	1331	1338	1346	1370	1466	1589	1610	1625	1661	1841	1907	1946	1973	2007	2014	2069	2102	2195	2222	2246	2255	2258	2284	2427	2453	2458	2467	2510	2520
																							*	*	*		*	*		
Chcu	C	T	C	A	C	T	G	C	C	C	G	A	C	T	C	T	C	T	C	G	C	G	C	G	G	A	C	G	G	$(AAC)_7$
OF07	.	C	T	.	.	C	.	.	.	.	A	.	.	.	T	.	.	G	.	.	.	.	.	A	.	T	.	.	.	$(AAC)_4$
OF14	.	.	T	.	.	.	.	.	T	.	.	.	.	.	.	.	.	.	.	.	.	.	.	.	C	.	.	.	.	$(AAC)_6$
OF16	.	.	T	.	.	.	.	.	.	.	.	.	.	.	.	.	.	.	A	.	.	A	.	A	.	T	G	.	.	$(AAC)_8$
OF18	.	.	T	.	.	.	T	T	.	.	.	.	.	.	.	.	.	.	.	.	A	.	A	.	.	.	.	.	A	$(AAC)_7$
OF19	.	.	T	.	.	.	T	T	.	.	.	.	.	.	.	.	.	.	.	.	.	.	.	A	.	T	.	.	.	$(AAC)_7$
OF21	.	.	T	.	.	C	.	.	.	.	.	C	.	.	.	.	.	.	.	.	.	.	.	.	.	T	.	.	.	$(AAC)_5$
OF28	.	.	T	.	.	.	T	T	.	T	.	.	.	.	.	C	.	.	.	.	.	.	.	A	.	T	.	.	.	$(AAC)_5$
OF31	.	.	T	.	.	.	.	.	.	.	.	.	.	.	.	.	G	.	.	A	.	.	.	.	.	T	.	.	.	$(AAC)_7$
OF40	.	.	T	.	.	.	T	T	.	.	.	.	T	A	.	.	.	.	.	.	.	.	.	A	.	T	.	C	.	$(AAC)_6$
OF44	.	.	T	.	A	.	.	.	.	.	.	.	.	.	.	.	.	.	.	.	.	.	.	.	C	.	.	.	.	$(AAC)_9$
OF47	.	.	T	.	.	.	T	T	.	.	.	.	.	.	.	.	.	.	.	.	.	.	.	.	.	T	.	.	.	$(AAC)_5$
OF54	T	.	.	C	.	.	.	.	.	.	A	.	.	.	.	.	.	.	.	.	.	.	.	A	C	T	.	.	.	$(AAC)_6$
OF58	.	.	T	.	.	.	T	T	.	.	.	.	.	.	.	.	.	.	.	.	.	.	A	.	.	T	.	.	.	$(AAC)_7$
OF60	.	.	T	.	.	.	T	T	.	.	.	.	.	.	.	.	.	.	.	.	.	.	.	A	.	T	.	.	.	$(AAC)_7$
OF78	.	.	T	.	.	.	T	T	.	.	.	.	.	.	.	.	.	.	.	.	.	A	.	A	.	T	.	.	.	$(AAC)_7$
Dmad	.	.	T	.	.	C	T	T	.	.	.	.	.	.	.	.	G	.	.	.	.	.	.	A	.	T	.	.	.	$(AAC)_4$
Dgua	.	G	T	.	.	C	.	.	.	.	.	.	.	.	.	.	A	.	.	.	.	.	.	A	.	T	.	.	.	$(AAC)_5$

									I3						E4	3'												
	2589	2595	2720	2738	2810	2852	2915	2967	3089	3090	3097	3098	3099	3100	3174	3281	3293	3308	3311	3326	3334	3342	3345	3346	3349	3357	3379	3419
	*	*						*																				
Chcu	A	G	A	C	T	C	T	C	C	G	A	T	T	C	G	G	A	T	G	C	C	T	G	T	C	C	G	A
OF07	.	.	C	.	.	A	.	A	.	.	.	C	A	G	A	.	.	.	.	.	T	.	A	.	.	.	.	.
OF14	.	.	.	.	.	A	.	.	.	.	.	C	A	G	A	.	.	.	T	.	.	.	A	.	.	.	A	.
OF16	.	.	.	.	.	A	C	.	.	.	.	C	A	G	.	.	.	.	.	.	.	.	A	.	.	.	A	.
OF18	.	.	.	.	.	A	.	.	.	.	.	C	A	G	.	.	T	.	.	.	.	.	A	.	.	.	A	.
OF19	.	.	.	.	.	A	.	.	.	.	.	C	A	G	A	.	.	.	.	.	.	.	.	.	.	.	.	.
OF21	.	.	.	.	.	A	.	.	.	.	.	C	A	G	A	.	.	.	.	.	.	.	A	.	.	T	A	.
OF28	.	A	.	.	.	A	.	.	T	T	G	C	A	G	A	.	.	.	.	.	.	C	A	.	.	.	.	.
OF31	.	.	C	.	.	A	.	.	.	.	.	C	A	G	A	.	.	.	.	.	.	.	A	.	.	.	A	.
OF40	.	.	.	.	.	A	.	.	T	T	.	C	A	G	.	.	.	C	.	.	.	.	A	.	.	.	A	.
OF44	.	.	C	.	.	A	.	.	.	.	.	C	A	G	A	.	.	.	.	A	.	.	A	C	.	.	.	.
OF47	.	.	.	T	.	A	.	.	.	.	.	C	A	G	.	.	.	.	.	.	.	C	A	.	.	.	A	.
OF54	.	.	.	.	G	A	.	.	.	.	.	C	A	G	.	C	.	.	.	.	.	.	.	.	A	.	A	T
OF58	.	.	.	.	.	.	.	.	.	.	.	C	A	G	A	.	.	.	.	.	.	.	A	.	.	.	A	.
OF60	.	.	.	.	.	A	.	.	.	.	.	C	A	G	A	.	.	.	.	.	.	.	.	.	.	.	.	.
OF78	T	.	.	.	.	A	.	.	.	.	.	C	A	G	A	.	.	.	.	.	.	.	.	.	.	.	A	.
Dmad	.	.	.	.	.	A	.	.	.	.	.	C	A	G	A	.	.	.	.	.	.	.	A	.	.	.	.	.
Dgua	.	.	.	.	.	A	.	.	.	.	.	A	A	G	.	.	.	.	.	.	.	.	A	.	.	.	.	.

	3'																																									
	3420	3426	3450	3465	3492	3500	3503	3506	3552	3607	3613	3628	3632	3689	3700	3701	3702	3703	3720	3724	3731	3732	3742	3849	3949	3963	4009	4023	4025	4032	4033	4039	4043	4091	4150	4152	4203	4205	4212	4213	4214	4227
Chcu	A	G	A	C	T	T	T	T	G	C	G	A	T	G	A	T	T	C	C	A	A	A	G	A	A	G	C	T	C	T	A	A	A	A	G	T	T	C	A	C	-	T
OF07	.	.	.	.	.	.	.	.	.	.	.	.	.	.	.	.	.	.	.	.	.	.	.	.	.	.	.	.	.	.	.	d_2	.	.	.	.	.	.	.	.	-	.
OF14	G	.	.	.	.	.	.	.	.	.	.	.	.	.	.	.	.	.	.	.	.	.	.	.	C	.	.	.	.	.	.	.	.	.	.	.	.	.	.	.	-	.
OF16	G	.	.	.	.	.	.	.	d_3	.	.	.	.	.	.	.	.	.	.	.	.	.	.	.	.	.	.	.	.	.	.	.	.	.	.	.	.	.	.	.	-	.
OF18	.	.	.	.	.	.	.	.	d_3	.	.	.	d_{57}	C	.	.	.	.	.	.	.	.	.	-	.	.	.	A	.	.	.	.	G	.	.	.	.	.	.	.	-	.
OF19	G	.	.	.	.	.	.	.	d_3	.	.	C	.	.	.	.	.	.	T	.	T	.	T	.	.	.	.	.	.	.	.	.	.	.	.	.	.	.	G	T	i_3	.
OF21	G	.	.	.	.	.	.	.	.	.	.	C	.	.	.	A	A	d_3	T	.	T	.	.	.	.	.	G	.	.	.	.	.	G	.	.	.	.	.	G	T	i_3	.
OF28	G	.	G	.	.	.	A	C	d_3	A	C	.	.	.	.	.	.	.	.	.	T	.	.	-	.	.	.	.	.	.	.	.	.	.	.	.	.	.	G	T	i_3	.
OF31	G	.	.	.	.	.	.	.	.	.	.	.	.	.	.	C	.	.	T	.	T	C	.	.	.	C	.	.	.	.	.	d_1	.	.	.	.	.	.	.	.	-	.
OF40	.	.	G	.	.	.	A	C	d_3	.	.	.	.	.	T	.	.	.	T	T	T	.	.	-	.	.	.	.	.	.	.	.	.	.	.	.	A	.	.	.	-	A
OF44	.	.	.	.	.	.	.	.	.	.	.	.	.	.	.	.	.	.	.	.	.	.	.	.	.	C	.	.	A	.	.	.	.	.	.	.	C	.	.	.	-	.
OF47	.	.	.	.	.	G	.	.	.	.	.	.	.	.	.	.	.	.	T	.	T	.	.	.	.	.	.	.	.	A	C	.	.	.	.	.	.	.	.	.	-	.
OF54	.	A	.	.	.	.	.	.	d_3	.	.	.	.	.	.	.	.	.	.	.	T	.	.	.	.	C	.	.	.	.	.	.	G	.	.	.	.	.	.	.	-	.
OF58	G	.	.	.	C	.	.	.	d_3	.	A	.	.	.	.	.	.	.	.	.	.	.	.	-	C	.	.	.	.	.	.	d_2	.	T	.	.	.	.	.	.	-	.
OF60	G	.	.	.	.	.	.	.	d_3	.	.	C	.	.	.	.	.	.	T	.	T	.	T	.	.	.	.	.	.	.	.	.	.	.	.	.	.	.	G	T	i_3	.
OF78	.	.	.	A	.	A	.	.	d_3	.	.	.	.	.	.	.	.	.	.	.	.	.	.	-	C	.	.	.	.	.	.	.	.	.	T	C	.	A	.	.	-	.
Dmad	.	.	.	.	.	A	.	.	.	A	.	.	.	.	.	.	.	.	T	.	T	.	.	.	.	.	.	.	.	.	.	.	.	.	T	.	.	.	.	.	-	.
Dgua	.	.	.	.	.	A	.	.	.	A	.	.	.	.	.	.	.	.	T	.	T	.	.	.	.	-	.	.	.	A	.	.	G	.	T	C	.	A	.	.	-	A

O) *Ph-d*

5’ I1 E2 I2 E3 I3 E5

	8	172	185	251	322	379	385	528	562	673	813	879	916	970	1054	1105	1137	1143	1177	1278	1308	1519	1569	1668	1752	2013	2017	2110
					*			*		*				*					*									*
Chcu	T	-	G	C	C	-	C	C	G	G	C	C	C	G	C	C	C	C	C	C	A	d_6	G	G	A	T	-	T
OF07	.	-	.	.	A	-	.	.	.	.	.	.	T	.	.	.	.	.	.	.	C	d_6	A	.	.	.	-	A
OF14	C	-	.	.	.	-	.	.	.	.	.	.	T	.	.	.	.	.	.	.	.	d_6	.	.	.	.	-	A
OF15	C	-	.	.	.	-	.	.	.	A	.	.	T	.	.	.	.	.	.	.	.	d_6	.	.	.	.	i_6	A
OF16	.	-	.	.	.	-	d_6	.	.	.	.	.	T	.	.	.	.	.	.	.	.	d_6	.	.	.	.	i_6	A
OF18	.	i_6	.	.	.	-	.	.	.	.	.	.	.	.	.	.	.	.	.	.	C	d_6	A	T	.	.	-	A
OF19	.	-	A	A	.	-	.	.	d_6	.	.	.	T	.	.	.	.	.	.	.	C	d_6	A	.	G	.	-	A
OF23	.	-	.	.	.	-	d_6	.	.	.	.	.	T	.	.	.	.	.	.	.	.	d_6	.	.	.	C	-	A
OF28	.	-	.	.	.	i_6	.	.	.	.	.	.	T	.	.	.	.	.	.	.	.	d_6	.	.	.	.	i_6	A
OF42	C	-	.	.	.	-	.	.	.	.	.	.	T	.	.	.	T	A	A	.	.	d_6	.	.	.	.	i_6	A
OF44	C	-	.	.	.	-	.	.	.	.	.	T	T	.	.	.	.	.	.	.	.	d_6	.	.	.	.	i_6	A
OF47	.	-	.	.	.	-	.	.	.	.	.	.	.	T	.	.	.	.	.	.	C	C	.	.	.	.	-	A
OF49	.	-	.	.	.	-	.	.	.	.	.	.	.	.	.	.	.	.	.	T	C	d_6	.	.	.	.	i_6	A
OF58	C	-	.	.	.	-	.	.	.	.	.	T	T	.	.	T	.	.	.	.	.	d_6	.	.	.	.	i_6	A
OF60	.	-	A	A	.	-	.	.	d_6	.	.	.	T	.	.	.	.	.	.	.	C	d_6	A	.	G	.	-	A
OF74	.	-	.	.	.	-	.	A	.	.	T	.	.	.	d_6	.	.	.	.	.	.	d_6	.	.	.	.	i_6	A
Dmad	.	-	.	.	.	-	.	.	.	.	.	.	.	.	.	.	.	A	A	.	C	C	.	.	G	.	-	A
Dgua	.	-	.	.	.	-	.	.	.	A	.	.	.	.	.	.	.	A	A	.	C	C	.	.	G	.	-	A

	2149	2227	2356	2690	2731	2732	2738	2876	2967	3425	3524	3545	3561	3818	3845	3851	3947
													*				
Chcu	$(CAR)_{18}$	$(CAR)_{17}$	C	A	A	T	T	C	$(CAR)_{24}$	C	$(CAG)_4$	d_3	A	$(CAG)_3$	C	$(CAR)_{13}$	$(CAR)_{12}$
OF07	$(CAR)_{18}$	$(CAR)_{17}$	.	G	T	.	.	.	$(CAR)_{24}$	.	$(CAG)_7$	C	.	$(CAG)_3$	d_6	$(CAR)_{13}$	$(CAR)_{12}$
OF14	$(CAR)_{17}$	$(CAR)_{15}$	.	G	.	C	.	.	$(CAR)_{26}$	.	$(CAG)_5$	C	.	$(CAG)_6$	.	$(CAR)_{13}$	$(CAR)_{12}$
OF15	$(CAR)_{16}$	$(CAR)_{19}$	.	G	T	.	.	.	$(CAR)_{25}$	.	$(CAG)_5$	C	.	$(CAG)_3$	.	$(CAR)_{13}$	$(CAR)_{12}$
OF16	$(CAR)_{18}$	$(CAR)_{17}$	.	G	.	.	.	.	$(CAR)_{25}$	.	$(CAG)_5$	C	.	$(CAG)_3$	.	$(CAR)_{13}$	$(CAR)_{12}$
OF18	$(CAR)_{17}$	$(CAR)_{17}$	.	.	.	.	.	.	$(CAR)_{26}$	.	$(CAG)_6$	C	.	$(CAG)_3$	.	$(CAR)_{13}$	$(CAR)_9$
OF19	$(CAR)_{17}$	$(CAR)_{17}$	.	G	.	C	G	.	$(CAR)_{25}$	.	$(CAG)_5$	C	.	$(CAG)_3$	.	$(CAR)_{13}$	$(CAR)_{12}$
OF23	$(CAR)_{18}$	$(CAR)_{17}$	.	.	.	.	.	.	$(CAR)_{22}$	.	$(CAG)_6$	C	.	$(CAG)_3$	d_6	$(CAR)_{13}$	$(CAR)_9$
OF28	$(CAR)_{16}$	$(CAR)_{19}$	.	G	T	.	.	.	$(CAR)_{26}$	.	$(CAG)_5$	C	.	$(CAG)_3$	.	$(CAR)_{13}$	$(CAR)_{15}$
OF42	$(CAR)_{18}$	$(CAR)_{19}$	.	G	T	.	.	.	$(CAR)_{25}$	.	$(CAG)_5$	C	.	$(CAG)_3$	d_6	$(CAR)_{13}$	$(CAR)_{15}$
OF44	$(CAR)_{15}$	$(CAR)_{17}$	.	G	T	.	.	.	$(CAR)_{25}$	.	$(CAG)_5$	C	T	$(CAG)_3$	.	$(CAR)_{14}$	$(CAR)_{12}$
OF47	$(CAR)_{17}$	$(CAR)_{19}$	.	.	.	.	.	T	$(CAR)_{20}$	.	$(CAG)_5$	C	.	$(CAG)_5$	d_6	$(CAR)_{13}$	$(CAR)_9$
OF49	$(CAR)_{18}$	$(CAR)_{17}$	.	.	.	.	.	.	$(CAR)_{25}$	.	$(CAG)_5$	C	.	$(CAG)_3$	.	$(CAR)_{13}$	$(CAR)_9$
OF58	$(CAR)_{18}$	$(CAR)_{17}$	.	G	.	.	.	.	$(CAR)_{25}$	d_{12}	$(CAG)_5$	C	.	$(CAG)_3$	.	$(CAR)_{13}$	$(CAR)_{12}$
OF60	$(CAR)_{17}$	$(CAR)_{17}$	.	G	.	C	G	.	$(CAR)_{25}$	.	$(CAG)_5$	C	.	$(CAG)_3$	.	$(CAR)_{13}$	$(CAR)_{12}$
OF74	$(CAR)_{17}$	$(CAR)_{19}$	d_6	G	.	.	.	.	$(CAR)_{25}$	.	$(CAG)_5$	C	.	$(CAG)_3$	.	$(CAR)_{15}$	$(CAR)_9$
Dmad	$(CAR)_{15}$	$(CAR)_{15}$	.	.	.	.	.	.	$(CAR)_{26}$		$(CAR)_5$	C	.	$(CAG)_3$	.	$(CAR)_{14}$	$(CAR)_6$
Dgua	$(CAR)_{18}$	$(CAR)_{15}$	.	.	.	.	.	.	$(CAR)_{18}$		$(CAR)_3$	C	.	$(CAG)_3$	.	$(CAR)_7$	$(CAR)_{14}$

	4057	4147	4183	4328	4384	4421	4451	4466	4472	4487	4496	4554	4612	4651	4711	4744	4753	4761	4804	4816	4842	4865	4903	5012	5174	5231	5237	5330	5343	5418	5426	5513	5543	5568
				*		*						*	*					*			*		*	*						*				
Chcu	C	G	G	G	C	G	-	i_6	d_9	d_9	-	A	C	C	G	G	G	C	G	C	G	-	G	G	A	G	C	A	-	G	G	G	C	i_6
OF07	.	.	.	.	.	.	-	-	A	A	-	.	.	.	A	.	A	.	.	.	.	-	.	.	.	.	.	.	i_6	.	.	.	.	i_6
OF14	.	.	.	.	.	.	-	-	A	A	-	.	.	.	.	.	A	.	.	.	.	i_{18}	A	.	.	.	.	.	i_6	.	.	.	.	i_6
OF15	.	.	.	.	.	.	-	-	A	A	-	.	.	T	.	T	A	.	.	.	.	-	.	.	T	.	.	C	-	.	.	.	.	i_6
OF16	.	.	.	.	.	.	-	-	A	A	-	.	.	T	.	T	A	.	.	.	.	-	.	.	.	.	.	.	-	.	.	.	.	i_6
OF18	.	.	.	.	.	A	-	-	A	A	-	.	G	.	.	.	A	.	.	.	.	i_9	.	.	.	.	.	.	-	.	C	.	.	i_6
OF19	.	.	.	.	.	.	-	-	A	A	-	.	.	T	.	T	A	.	.	.	.	i_9	.	.	.	.	.	.	i_6	.	.	.	.	i_6
OF23	.	.	.	.	.	.	-	-	A	A	-	.	.	T	.	T	A	T	.	.	.	-	.	.	.	.	.	.	-	.	.	.	.	i_6
OF28	.	.	.	.	.	.	-	-	A	A	-	.	.	T	.	.	A	.	.	.	.	-	.	.	.	.	.	.	-	.	.	.	.	i_6
OF42	.	.	.	.	.	.	-	-	A	A	-	.	.	.	.	.	A	.	T	T	.	i_9	.	.	.	.	.	.	i_6	.	.	T	T	i_6
OF44	.	.	.	.	.	.	-	-	A	A	-	.	.	T	.	T	A	.	.	.	.	-	.	.	.	A	T	.	-	.	.	.	.	i_6
OF47	T	.	.	.	G	.	-	-	d_6	d_6	i_{24}	.	.	.	.	.	A	.	.	.	.	i_9	.	.	.	.	.	C	-	.	.	.	.	i_6
OF49	.	A	.	A	.	.	-	-	A	A	-	.	G	.	.	.	A	.	.	.	A	-	.	C	.	.	.	.	-	T	.	.	.	-
OF58	.	A	.	.	.	.	i_{12}	i_6	d_9	d_9	-	.	G	.	.	.	A	.	.	.	.	i_{18}	.	.	.	.	.	.	i_6	.	.	.	.	i_6
OF60	.	.	.	.	.	.	-	-	A	A	-	.	.	T	.	T	A	.	.	.	.	i_9	.	.	.	.	.	.	i_6	.	.	.	.	i_6
OF74	.	.	A	.	.	.	-	-	A	A	-	G	.	T	.	T	A	.	.	.	.	-	.	.	.	.	.	.	-	.	.	.	.	i_6
Dmad	.	.	.	.	.	.	-	-	A	A	-	.	.	.	.	.	A	.	.	.	.	i_9	.	.	.	.	.	.	-	.	.	.	.	-
Dgua	.	.	.	.	.	.	i_{12}	i_6	d_9	d_9	-	.	.	.	T	.	A	.	.	.	.	i_9	.	.	.	.	.	.	-	.	.	.	.	-

E5 3’

	5574	5706	5741	5750	5769	5777	5781	5903	5987	6154	6179	6272	6312	6323	6324
		*													
Chcu	-	T	G	T	T	C	T	G	C	C	G	G	-	G	G
OF07	-	.	.	A	.	.	.	.	.	.	.	.	-	A	.
OF14	-	.	A	A	.	.	.	.	.	.	.	.	-	A	.
OF15	-	.	.	A	.	T	A	.	.	.	.	.	-	.	.
OF16	-	.	.	.	.	.	.	.	.	.	.	.	-	.	.
OF18	-	.	.	.	.	.	.	.	.	.	.	.	i_6	A	d_6
OF19	-	.	.	.	.	.	.	.	.	.	.	.	-	A	.
OF23	i_6	.	.	.	.	.	.	.	.	.	.	.	-	A	.
OF28	-	.	.	.	.	.	.	.	.	.	.	.	-	A	.
OF42	-	.	.	.	.	.	.	.	.	.	A	A	-	.	.
OF44	-	.	.	.	.	.	.	A	.	.	.	.	-	A	.
OF47	-	C	.	.	d_6	.	d_6	.	.	.	.	.	-	.	.
OF49	i_6	.	.	.	d_6	.	.	.	.	.	.	.	-	.	.
OF58	-	.	.	.	.	.	.	.	T	.	.	.	-	.	.
OF60	-	.	.	.	.	.	.	.	.	.	.	.	-	A	.
OF74	-	.	.	.	.	.	.	.	.	T	.	.	i_6	.	.
Dmad	i_6	.	.	A	.	.	d_6	.	.	.	A	.	-	A	.
Dgua	-	.	.	A	.	.	.	.	.	.	.	.	-	.	d_6

P) *Kdm2*

5' | E1 | I1 | I2 | E3

	27	129	182	191	193	288	299	518	602	724	800	842	894	996	1100	1114	1245	1268	1373	1384	1402	1412	1415	1447	1455	1493	1556	1557	1574	1576	1584	1588	1597	1632	1633	1647	1703	1754	1836	2007	2033	2034	2058	2080	2089	2103	2154	2160	2165	2229	2303	2331	2361	2392	2430	2481	2496	2520	2521	2559	2612	2657	2669	2711	2803	2811	2819	2836	2852	2934	3015	3057	3161	3197	3237	3271	3339	3345	3432	3434	3472	3474	3490	3507	3687	3690	3741	3901
Chcu	G	A	G	A	A	A	T	C	-	-	G	G	A	A	T	C	C	C	T	A	T	C	A	T	T	G	T	C	C	A	T	A	T	A	T	T	A	C	T	C	T	-	T	T	G	G	G	A	C	C	G	C	C	C	A	A	A	G	$(AC)_3$	C	T	C	C	G	-	T	i_4	$(GT)_5$	G	T	C	C	A	T	G	C	A	C	C	T	T	-	T	T	d_2	A	T	A
OF01	.	.	.	.	.	.	.	.	C	-	-	.	G	.	.	.	.	.	.	.	.	.	.	C	.	.	.	.	G	G	.	T	G	.	.	.	G	.	.	.	.	-	.	.	.	.	.	.	A	.	.	.	.	G	C	.	.	.	$(AC)_4$	.	.	.	.	.	-	.	i_4	$(GT)_5$	.	.	.	.	.	.	.	.	.	.	.	.	A	-	.	.	T	.	.	.
OF07	.	.	.	.	.	.	.	.	C	-	.	.	G	.	.	.	.	.	G	.	.	T	.	.	.	.	.	.	.	.	.	.	.	.	.	.	G	.	.	T	.	-	.	.	.	.	.	.	A	.	.	.	.	.	.	.	.	.	$(AC)_8$	.	.	.	.	T	i_4	.	-	$(GT)_8$	.	C	.	A	.	G	.	T	.	.	.	.	A	-	.	A	T	.	.	.
OF14	.	.	.	G	.	.	.	.	C	-	.	.	G	.	.	.	.	.	.	.	.	.	.	.	.	T	.	.	.	.	.	.	.	.	.	.	G	.	.	.	.	-	.	.	.	.	A	.	A	.	.	.	.	.	.	.	.	.	$(AC)_9$	.	.	.	.	T	i_4	.	-	$(GT)_3$	.	C	.	.	.	G	.	.	.	T	.	.	A	-	.	A	T	.	.	T
OF16	.	.	.	.	G	.	.	.	C	-	.	.	G	T	.	.	.	.	.	.	.	.	.	.	.	.	C	A	.	.	C	.	.	.	.	.	G	.	G	.	A	-	.	.	.	.	.	.	A	.	.	.	.	.	.	.	.	A	$(AC)_5$	T	.	.	.	T	i_4	.	-	$(GT)_6$	A	C	.	.	.	G	.	.	.	.	.	.	A	-	.	A	T	.	.	T
OF18	.	.	.	G	.	G	.	.	C	-	.	.	G	.	C	.	.	.	.	.	d_3	.	.	.	.	A	.	.	.	.	.	.	.	.	.	.	G	A	.	.	.	-	.	.	.	.	.	.	A	G	.	.	.	.	.	.	.	.	$(AC)_4$	.	.	.	.	.	-	.	i_4	$(GT)_5$	.	.	.	.	.	.	.	.	.	.	A	.	A	-	.	.	T	.	.	.
OF19	.	.	.	G	.	.	.	.	C	-	.	.	G	.	.	.	.	.	.	.	.	.	.	.	.	.	.	A	.	C	.	.	.	T	A	.	G	.	.	.	A	-	.	.	.	C	.	.	A	.	.	.	.	.	.	.	.	.	$(AC)_9$	.	.	.	.	T	i_4	.	-	$(GT)_6$	.	C	.	.	.	G	.	.	.	.	.	.	A	-	.	A	T	.	.	T
OF21	.	.	.	G	.	G	.	.	C	-	.	.	G	.	.	.	.	A	.	.	.	.	.	.	.	.	.	A	.	.	C	.	.	.	.	.	G	.	.	.	.	-	.	C	.	.	.	.	A	.	.	.	A	.	.	.	.	.	$(AC)_7$	.	.	.	.	T	i_4	.	i_4	$(GT)_6$	.	C	T	.	.	G	.	.	.	.	.	.	A	-	C	.	T	.	A	T
OF28	T	.	.	.	.	.	.	.	C	-	.	.	G	.	.	.	.	.	.	.	.	.	.	C	.	.	.	.	G	G	.	T	G	.	.	.	G	.	.	.	.	-	.	.	.	.	.	.	A	.	.	.	.	.	.	.	.	A	$C(AC)_{18}$	.	.	.	.	T	i_4	.	-	$(GT)_6$	A	C	.	.	.	G	.	.	.	.	.	.	A	-	.	A	T	T	.	T
OF31	.	.	.	.	.	.	.	T	C	-	.	T	G	.	.	.	.	.	.	.	.	.	.	.	.	.	.	.	.	.	.	.	.	.	.	.	G	.	.	.	.	-	.	.	.	.	.	.	A	.	.	.	.	.	.	T	T	.	$(AC)_4$	.	.	.	.	.	i_4	.	-	$(GT)_6$	.	C	.	.	.	.	.	.	.	.	.	A	A	C	.	A	T	.	.	T
OF40	.	.	.	.	.	.	.	.	C	-	.	.	G	.	.	.	.	.	.	.	.	.	.	.	.	.	.	A	.	.	C	.	.	.	.	.	G	.	G	.	A	-	.	.	.	.	.	.	A	.	.	.	.	.	.	.	.	.	$(AC)_5$	.	.	.	d_9	T	i_8	.	-	$(GT)_6$	.	C	T	.	.	G	.	.	.	.	.	.	A	-	.	A	T	.	A	T
OF47	.	.	.	.	.	.	.	.	C	-	.	.	G	.	.	.	.	.	.	.	.	.	.	.	.	.	.	.	.	.	.	.	.	.	.	.	G	.	.	.	.	i_6	.	.	.	.	.	.	A	.	.	.	.	.	.	.	.	.	$(AC)_7$	.	.	.	.	T	i_4	.	-	$(GT)_7$	.	C	.	.	.	G	A	.	.	.	.	.	A	-	.	A	T	.	.	T
OF54	.	.	.	.	.	.	.	.	C	-	.	.	G	.	.	.	.	.	G	G	.	T	.	.	.	.	.	.	.	.	.	.	.	.	.	.	G	.	.	T	.	-	.	.	.	.	.	.	A	.	T	.	.	.	.	.	.	.	$(AC)_8$	.	.	.	.	T	i_4	A	-	$(GT)_6$	.	C	.	.	.	G	.	T	.	.	.	.	A	-	.	A	T	.	A	T
OF58	.	G	C	.	.	.	A	.	C	i_4	.	.	G	.	.	A	G	.	.	.	.	.	G	C	A	.	.	.	.	.	.	T	G	.	.	d_6	G	.	.	.	.	-	A	.	T	.	.	T	A	.	.	.	.	.	.	.	.	.	$(AC)_4$	.	G	A	.	.	-	.	-	$(GT)_5$	.	C	.	.	.	G	A	.	C	.	.	.	A	-	.	A	T	.	.	T
OF60	.	.	.	G	.	.	.	.	C	-	.	.	G	.	.	.	.	.	.	.	.	.	.	.	.	.	.	A	.	C	.	.	.	T	A	.	G	.	.	.	A	-	.	.	.	C	.	.	A	.	.	.	.	.	.	.	.	.	$(AC)_9$	.	.	.	.	T	i_4	.	-	$(GT)_6$	.	C	.	.	.	G	.	.	.	.	.	.	A	-	.	A	T	.	.	T
OF78	.	.	.	.	.	.	.	.	C	-	.	.	G	.	.	.	.	.	.	.	.	.	.	C	.	.	.	.	G	G	.	T	G	.	.	.	G	.	.	.	.	-	.	.	.	.	.	.	A	.	.	A	.	.	.	.	.	.	$(AC)_3$	.	.	.	.	.	-	.	i_4	$(GT)_5$	.	.	.	.	C	.	.	.	.	.	.	.	A	-	.	.	T	.	A	T
Dmad	.	.	.	.	.	.	.	.	C	-	.	.	G	.	.	.	.	.	.	.	.	.	.	C	.	.	.	.	.	.	.	.	.	.	A	.	G	.	.	.	.	-	.	.	.	.	.	.	A	.	.	.	.	.	.	.	.	.	$ACTC(AC)_3$	.	.	.	.	.	-	-	-	$(GT)_7$	.	C	.	.	.	G	.	.	.	.	.	.	A	-	A	.	T	.	.	.
Dgua	.	.	.	.	.	.	.	.	C	-	.	.	G	.	.	.	.	.	.	.	.	.	C	C	.	.	.	.	.	.	.	.	.	.	.	.	G	.	.	.	.	-	.	.	.	.	.	.	A	.	.	.	A	.	.	.	.	.	$(AC)_4$	.	.	.	.	T	-	-	-	$T(GT)_2$	.	C	.	.	.	G	.	T	.	.	.	.	A	-	.	.	T	.	.	T

E3 | I3 | E4 | I4 | E5 | 3'

	4030	4264	4306	4381	4441	4636	4639	4723	5002	5062	5173	5215	5229	5259	5301	5348	5375	5385	5388	5409	5508	5592	5757	5788	5791	5796	5805	5958	5990	6009	6014	6016	6037	6077	6083	6125	6137	6164	6179	6212	6248	6287	6428	6452	6482	6519	6528	6534	6547	6617	6653	6686	6772	6799	6815	6941	7109	7185	7283	7298	7301	7364	7409	7436	7628	7656	7678	7705	7707	7717	7721	7732	7773	7789	7797	7805	7830	7831	7833	7961	7991	8013	8024	8030	8034	8035	8046	8049	8056	8127	8129	8157	8166
																*	*							*	*																					*			*				*	*												*																												
Chcu	A	C	C	C	C	T	T	G	A	C	G	T	G	C	A	C	A	G	G	T	T	C	C	A	G	C	C	A	G	A	A	C	G	C	G	T	C	C	C	T	C	T	G	A	C	G	G	i_6	C	G	C	T	G	G	T	G	C	C	C	A	C	C	C	G	G	G	A	G	A	-	-	A	G	C	C	G	G	C	A	G	T	G	C	T	G	T	T	G	C	A	G	A	A
OF01	.	.	.	A	.	.	.	.	.	.	A	.	A	.	.	.	.	.	T	.	.	.	.	.	.	.	G	.	.	.	.	.	.	.	.	.	A	.	.	A	.	.	C	.	.	A	.	-	G	.	A	C	.	.	C	.	.	.	.	.	.	T	.	C	.	.	.	T	.	-	-	T	.	.	.	.	.	.	.	.	.	.	.	.	.	.	A	T	.	.	.	.	.
OF07	C	.	T	.	.	.	.	.	.	T	.	.	A	.	G	G	.	.	.	.	.	.	.	.	.	.	.	.	.	.	.	T	.	.	.	.	.	.	.	.	.	.	C	.	T	.	.	-	G	.	A	C	.	.	C	.	.	.	.	.	.	.	.	.	A	.	.	.	.	-	A	.	.	.	.	.	.	.	.	.	.	.	.	.	.	.	A	.	.	.	.	.	.
OF14	.	.	T	.	.	.	.	.	.	.	.	.	.	T	.	.	.	.	.	.	.	T	.	.	.	.	.	.	T	.	.	.	.	.	.	.	.	T	.	.	.	.	C	.	.	.	.	-	G	.	A	C	.	.	C	A	.	.	.	.	.	T	.	.	.	.	.	.	.	-	-	.	.	.	.	.	.	.	G	.	.	.	A	.	A	G	A	T	.	G	.	.	.
OF16	.	.	.	.	.	.	.	.	.	.	.	.	.	.	.	.	.	A	.	.	C	T	.	.	.	.	.	.	.	.	.	.	.	.	.	.	.	.	.	.	.	.	C	.	.	.	.	-	.	.	A	C	C	A	C	.	T	.	.	.	.	T	.	.	.	.	.	.	.	A	-	.	.	.	.	.	.	.	.	.	.	.	.	.	.	.	A	.	.	.	.	.	.
OF18	.	.	.	.	.	.	.	.	.	.	.	.	.	.	.	.	.	.	.	.	.	.	.	.	.	.	.	.	.	.	.	.	.	.	.	.	.	.	.	.	.	.	.	.	T	.	.	-	G	.	A	C	.	.	.	.	.	.	.	.	.	.	.	.	.	.	.	.	.	-	-	.	.	.	.	.	.	.	.	.	.	.	.	.	.	.	.	.	.	.	.	.	.
OF19	.	.	.	.	.	C	C	.	T	.	.	.	A	.	.	.	.	.	T	.	.	.	.	.	.	.	G	.	.	.	.	.	A	.	.	C	.	.	.	A	.	C	C	.	.	.	.	-	G	.	A	C	.	.	C	.	.	.	.	.	.	.	.	.	.	.	.	.	.	-	-	.	T	.	.	.	.	.	.	.	.	.	.	.	.	.	.	.	.	.	.	.	.
OF21	.	.	.	.	.	.	.	.	.	.	.	.	A	.	.	.	.	.	T	A	.	.	.	.	.	.	.	.	.	.	.	.	.	A	.	.	.	.	.	A	T	.	C	.	.	.	.	-	G	.	A	C	.	.	C	.	.	.	.	T	.	T	.	.	.	.	.	.	.	-	-	.	.	.	.	.	.	.	.	.	.	.	.	.	.	.	A	T	.	.	.	.	.
OF28	.	.	.	.	T	.	.	.	.	.	.	.	A	.	.	.	.	.	T	.	.	.	.	.	.	.	.	.	.	.	.	.	.	A	.	.	.	.	.	A	.	.	.	T	.	.	.	-	G	.	A	C	.	.	C	.	.	.	.	.	.	.	.	.	.	.	T	.	.	-	-	.	.	.	.	A	.	.	.	.	.	d_{11}	.	.	.	.	A	.	.	.	.	G	T
OF31	.	.	.	.	.	.	.	.	.	T	.	.	A	.	.	.	T	.	T	.	.	.	.	.	.	.	.	.	.	G	.	.	.	.	.	.	.	.	T	.	.	.	C	.	.	.	d_6	-	G	.	A	C	C	A	C	.	.	T	.	.	T	.	.	.	.	.	.	.	.	-	-	.	.	.	.	.	.	.	.	.	.	.	A	.	A	G	A	T	.	G	.	G	T
OF40	.	.	T	.	.	.	.	.	.	T	.	.	A	.	G	.	.	.	T	.	.	.	A	.	.	.	.	.	.	.	.	.	.	.	.	C	.	.	.	A	.	.	T	.	.	.	.	-	G	.	A	C	C	A	C	.	.	.	.	T	.	.	.	.	.	.	.	.	T	-	-	.	.	.	.	.	.	.	.	.	.	.	.	.	.	.	A	.	A	.	.	.	.
OF47	.	T	T	.	T	.	.	.	.	T	.	.	A	.	.	.	.	.	T	.	.	.	.	.	.	.	.	.	.	.	.	.	.	.	.	.	.	.	.	A	.	.	C	.	.	.	.	-	G	T	A	C	.	A	C	.	.	.	.	.	.	.	T	.	.	.	.	.	.	-	-	.	.	.	.	.	.	.	G	-	.	.	.	.	.	.	A	T	.	.	C	.	.
OF54	.	.	T	.	.	C	C	T	.	T	.	.	A	.	G	.	.	.	T	.	.	.	A	.	.	.	.	.	.	.	.	.	.	.	.	C	.	.	.	A	.	.	T	.	.	.	.	-	G	.	A	C	C	A	C	.	.	.	.	.	.	.	.	.	A	.	.	.	.	-	-	.	.	.	.	.	.	.	.	.	.	.	.	.	.	.	A	.	A	.	.	.	.
OF58	.	.	.	.	T	C	C	.	.	.	.	G	.	.	.	.	.	.	T	.	.	.	.	.	T	T	T	C	.	.	C	.	.	.	A	.	.	.	.	.	.	.	C	.	.	.	.	-	G	.	A	C	.	.	C	.	.	.	T	.	.	.	.	.	.	T	.	.	.	-	-	.	.	A	.	.	A	A	.	.	.	.	.	C	.	.	.	.	.	.	.	.	.
OF60	.	.	.	.	.	C	C	.	T	.	.	.	A	.	.	.	.	.	T	.	.	.	.	.	.	.	G	.	.	.	.	.	A	.	.	C	.	.	.	A	.	C	C	.	.	.	.	-	G	.	A	C	.	.	C	.	.	.	.	.	.	.	.	.	.	.	.	.	.	-	-	.	T	.	.	.	.	.	.	.	.	.	.	.	.	.	.	.	.	.	.	.	.
OF78	.	.	T	.	.	C	C	T	.	T	.	.	A	.	.	.	.	.	T	.	.	.	.	G	.	.	.	.	.	.	.	.	.	.	.	C	.	.	.	A	.	.	C	.	.	.	.	-	G	.	A	C	.	.	C	.	.	.	.	.	.	.	.	.	.	.	.	.	.	-	-	.	.	.	T	.	.	.	.	.	G	.	.	.	.	.	A	.	.	.	.	G	T
Dmad	.	.	.	.	.	C	C	.	.	.	.	.	C	.	.	.	.	T	T	A	.	.	.	.	.	.	G	C	.	.	.	.	.	.	.	.	.	.	.	.	.	.	C	.	.	.	.	-	G	.	A	C	.	.	C	.	.	.	.	.	.	A	.	.	.	.	-	.	.	-	-	.	.	.	.	.	.	.	.	.	.	.	.	.	A	G	A	.	.	.	.	G	.
Dgua	.	.	.	A	.	.	C	.	.	.	.	.	.	.	G	.	.	.	T	A	.	.	.	.	.	.	G	C	T	.	.	.	.	.	.	.	.	.	.	.	.	.	C	.	.	.	.	-	G	.	A	C	.	.	C	.	.	.	.	.	.	.	.	.	.	.	-	.	.	-	-	.	.	.	.	.	.	.	.	.	.	.	.	.	A	G	A	.	.	.	.	G	.

continued on next page

P) *Kdm2*

continued from previous page

3’

	8188	8189	8195	8206	8215	8220	8224	8243	8296	8338	8382	8390	8436	8441	8442	8457	8462	8464	8486	8494	8495	8525	8534	8542	8568	8572	8687	8700
Chcu	G	A	A	T	C	A	A	T	C	-	A	T	T	T	G	G	C	G	C	-	T	A	C	T	T	C	T	T
OF01	.	.	.	.	.	.	.	.	.	-	.	.	.	C	.	C	A	.	.	-	.	T	.	.	.	.	.	.
OF07	.	.	.	.	.	.	.	.	.	-	.	A	.	.	.	.	.	A	.	-	.	.	.	.	.	.	.	.
OF14	.	.	.	A	.	.	.	.	T	-	.	.	.	.	T	.	.	.	.	-	.	T	T	.	.	.	.	C
OF16	.	.	.	.	.	C	.	-	.	-	.	.	.	.	.	.	.	.	.	-	.	.	.	G	.	T	.	.
OF18	.	.	.	.	.	.	.	.	.	-	.	.	.	.	.	.	.	.	.	-	.	.	.	.	.	.	.	.
OF19	.	.	.	.	.	.	.	.	.	-	.	.	C	.	.	.	.	.	.	-	.	T	.	.	.	.	.	.
OF21	.	.	.	.	.	.	.	.	.	-	.	.	.	.	.	.	.	.	.	-	.	T	.	.	C	.	.	C
OF28	T	.	.	.	.	.	.	.	.	-	.	.	.	.	.	.	.	.	A	-	.	T	.	.	.	T	.	.
OF31	.	d_2	.	.	.	.	.	.	.	-	.	.	.	C	.	C	A	.	.	-	.	.	.	G	.	T	.	.
OF40	.	.	.	.	.	.	.	.	.	-	.	.	.	.	.	.	.	.	.	T	.	T	.	.	C	.	.	C
OF47	.	.	.	.	T	.	.	.	.	G	T	.	.	.	.	C	A	.	.	-	A	T	.	.	C	.	C	.
OF54	.	.	.	.	.	.	.	.	.	-	.	.	.	.	.	.	.	.	A	-	.	T	.	.	.	T	.	.
OF58	.	.	G	.	.	.	.	.	.	-	.	.	.	.	.	.	.	.	.	-	.	.	.	.	.	.	.	.
OF60	.	.	.	.	.	.	.	.	.	-	.	.	C	.	.	.	.	.	.	-	.	T	.	.	.	.	.	.
OF78	.	d_2	.	.	.	.	C	.	.	-	.	.	.	.	.	.	.	.	.	T	.	T	.	.	C	.	.	C
Dmad	.	.	.	.	.	.	.	.	T	-	.	.	.	.	.	.	.	.	.	-	.	T	.	.	.	.	.	.
Dgua	.	.	.	.	.	.	.	.	.	-	.	.	.	.	.	-	-	-	.	-	.	T	.	.	.	.	.	.
