# Supplementary material for: Molecular population genetics of the Polycomb genes in Drosophila subobscura
Source: PLoS One. 2017 Sep 14;12(9):e0185005. doi: 10.1371/journal.pone.0185005 (PMC5599051; doi:10.1371/journal.pone.0185005)
Supplement: S2 Fig — Gray bars indicate the contribution to the overall χ2-like test statistic due to silent divergence between D. subobscura and D. guanche. Blue bars indicate the corresponding contribution due to silent polymorphism. The highest contribution of Pho both to divergence and polymorphism is significant by the maximum cell value test (see text). (PDF) [file pone.0185005.s004.pdf]

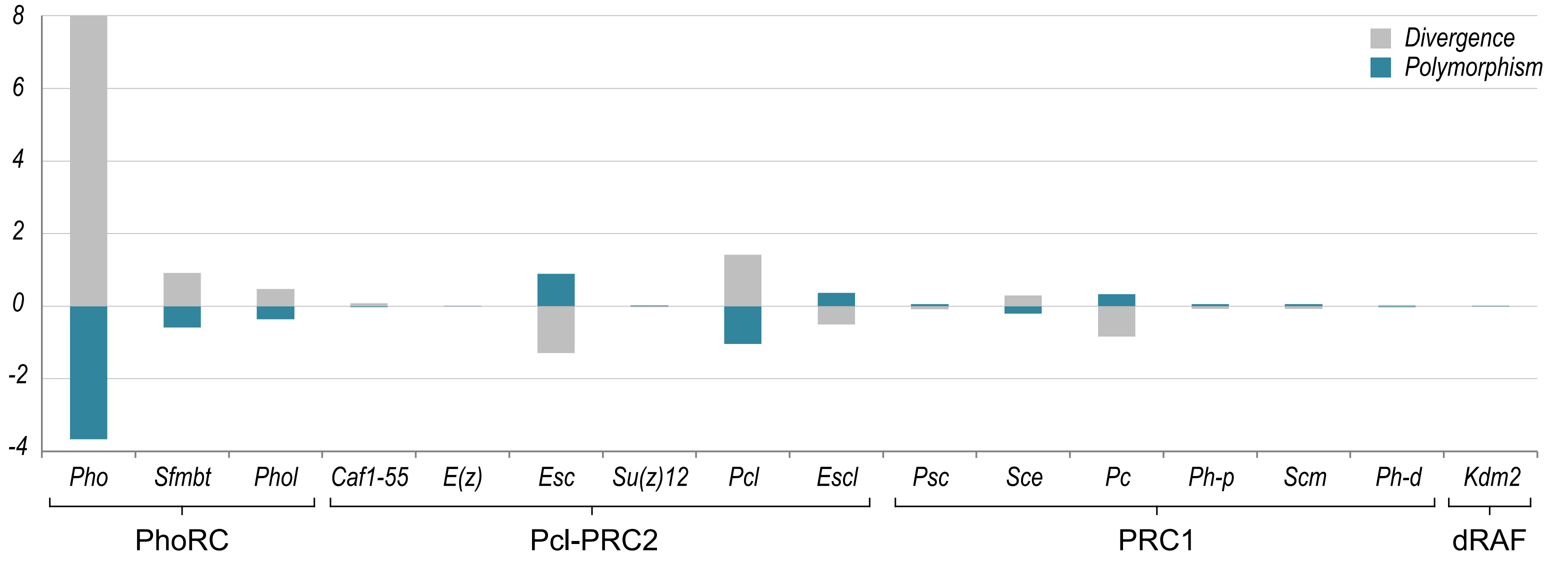

S2 Fig. Contribution of each PcG gene to the multilocus HKA test. Gray bars indicate the contribution to the overall χ^2-like test statistic due to silent divergence between *D. subobscura* and *D. guanche*. Blue bars indicate the corresponding contribution due to silent polymorphism. The highest contribution of *Pho* both to divergence and polymorphism is significant by the maximum cell value test (see text).
