# Supplementary material for: Molecular population genetics of the Polycomb genes in Drosophila subobscura
Source: PLoS One. 2017 Sep 14;12(9):e0185005. doi: 10.1371/journal.pone.0185005 (PMC5599051; doi:10.1371/journal.pone.0185005)
Supplement: S3 Fig — Sliding window plots of the distribution of nucleotide diversity in D. subobscura (π, black line) and of nucleotide divergence between D. subobscura and D. guanche (K, gray line). Windows include 50 sites with successive displacements of 5 sites. The x-axis indicates nucleotide sites across the gene region and the y-axis indicates nucleotide diversity or divergence. Solid boxes in the lower part of the figure indicate the coding exons and thin lines show flanking regions and introns. Pho and Su(z)12 plots are shown in Fig 3. (PDF) [file pone.0185005.s005.pdf]

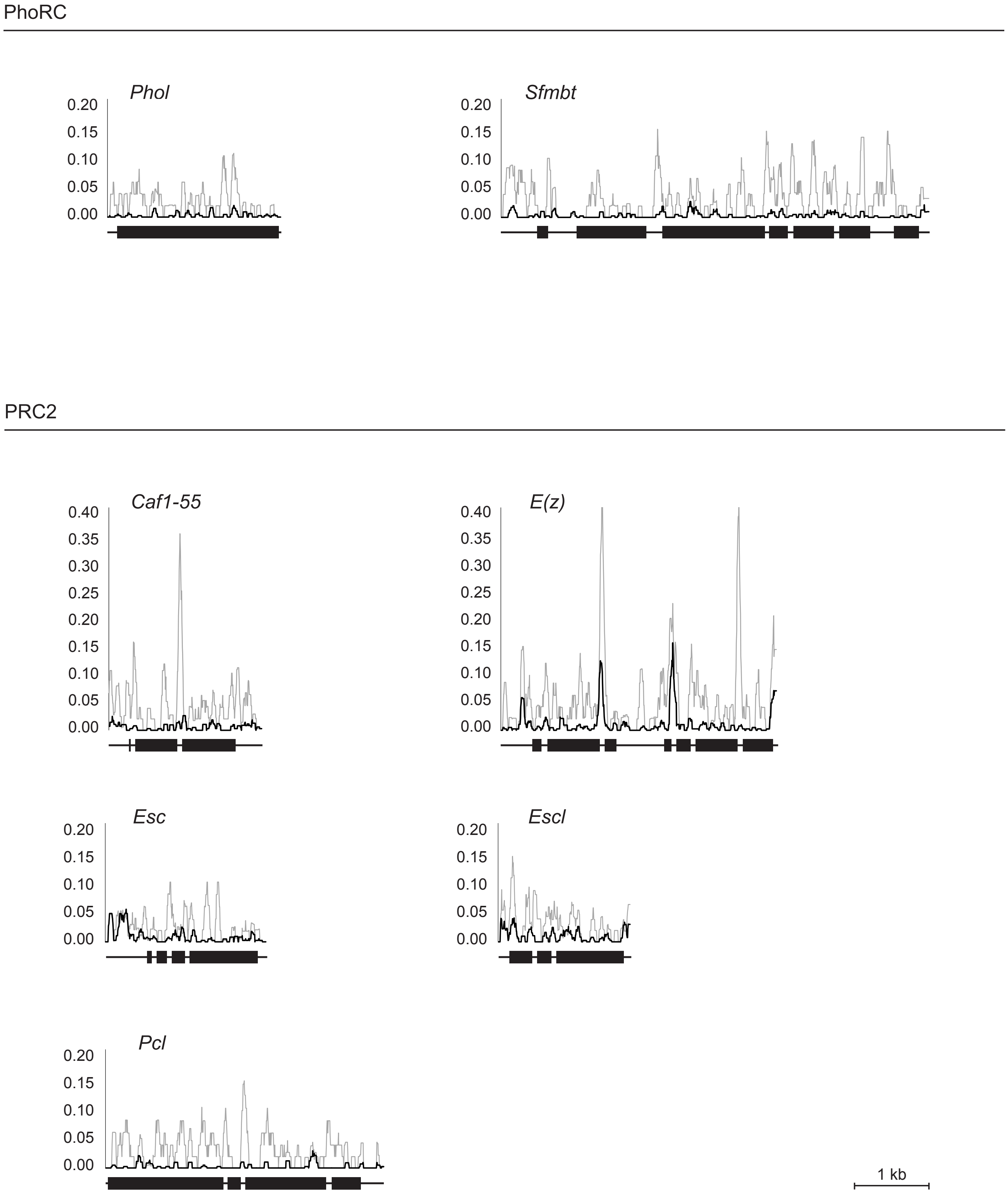

S3 Fig. Nucleotide diversity and divergence along PcG genes. Sliding window plots of the distribution of nucleotide diversity in *D. subobscura* (π, black line) and of nucleotide divergence between *D. subobscura* and *D. guanche* (*K*, gray line). Windows include 50 sites with successive displacements of 5 sites. The *x*-axis indicates nucleotide sites across the gene region and the *y*-axis indicates nucleotide diversity or divergence. Solid boxes in the lower part of the figure indicate the coding exons and thin lines show flanking regions and introns. *Pho* and *Su(z)12* plots are shown in Fig 3.

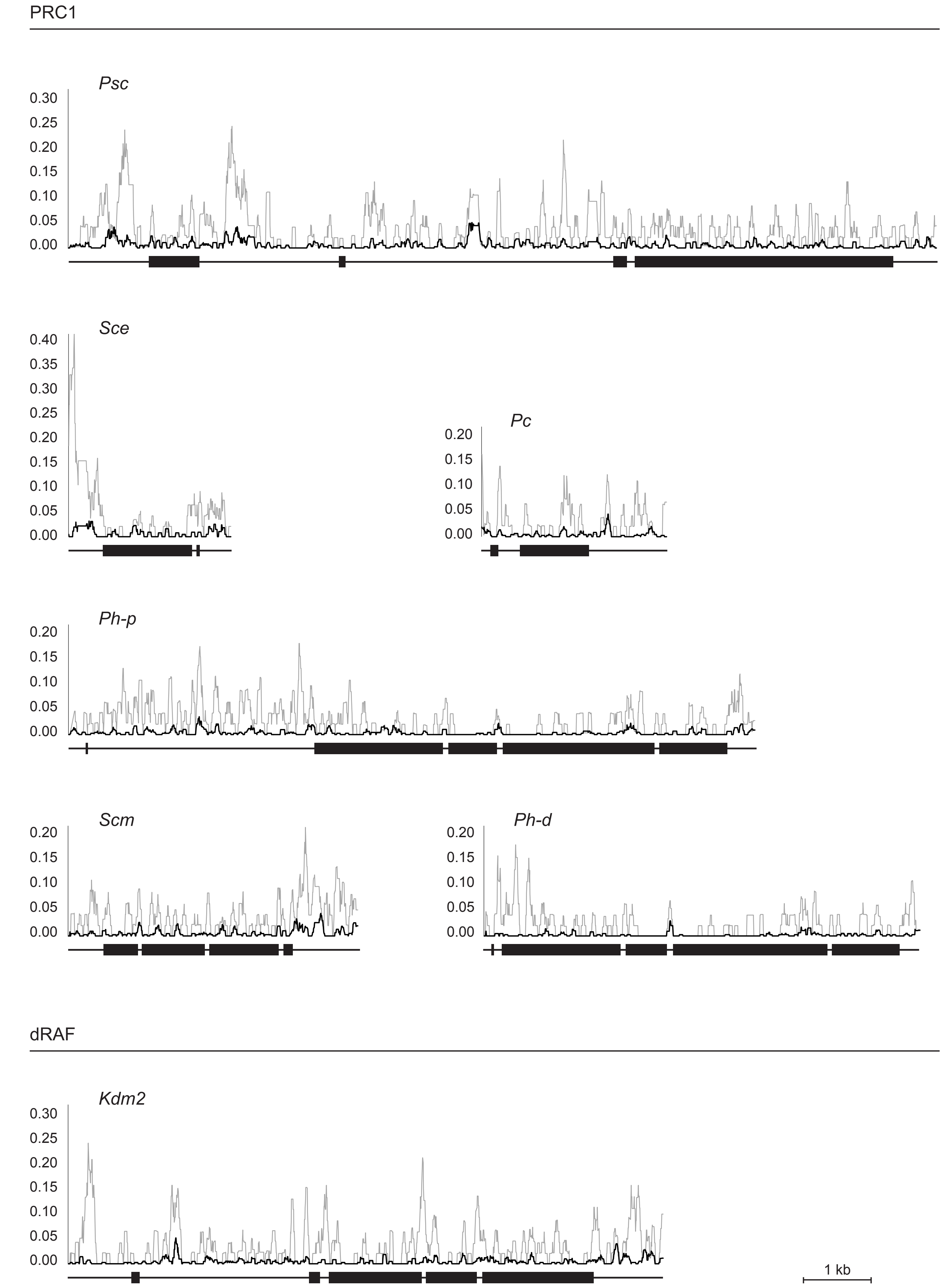

S3 Fig. Continued from previous page.
